# Regulation of Hippo/YAP axis in colon cancer progression by the deubiquitinase JOSD1

**DOI:** 10.1038/s41420-024-02136-7

**Published:** 2024-08-14

**Authors:** Yanan Sun, Dongyi Liu, Xiaobo Zhang, Peng Su, Xin Li, Zhongbo Li, Yingwen Gai, Jingying Li, Zhiyong Yang, Yinlu Ding, Jian Zhu, Xiaodong Tan

**Affiliations:** 1 Department of General Surgery, The Second Hospital, Cheeloo College of Medicine, Shandong University, Jinan, 250033 Shandong Province P.R. China; 2 Department of Anesthesiology, The Second Hospital, Cheeloo College of Medicine, Shandong University, Jinan, 250033 Shandong Province P.R. China; 3 Department of General surgery, Shengjing Hospital of China Medical University, Shenyang, 110000 Liaoning Province P.R. China; 4 Department of Pathology, Qilu Hospital, Cheeloo College of Medicine, Shandong University, Jinan, 250012 Shandong Province P.R. China; 5 Xinxiang Key Laboratory of Tumor Migration and Invasion Precision Medicine, School of Medical Technology, Xinxiang Medical University, Xinxiang, 453003 Henan Province P.R. China; 6 Department of Health Management, Shengjing Hospital of China Medical University, Shenyang, 110000 Liaoning Province P.R. China; 7 Deartment of Cardiology, Shengjing Hospital of China Medical University, Shenyang, 110000 Liaoning Province P.R. China; 8 Department of Gastrointestinal Surgery, The Second Hospital, Cheeloo College of Medicine, Shandong University, Jinan, 250033 Shandong Province P.R. China

**Keywords:** Oncogenes, Cell signalling

## Abstract

Colon cancer is a prevalent malignancy, while recent studies revealed the dys-regulation of Hippo signaling as the important driver for colon cancer progression. Several studies have indicated that post-translational modifications on YAP play crucial roles in both Hippo signaling activity and cancer progression. This raises a puzzling question about why YAP/TAZ, an auto-inhibitory pathway, is frequently over-activated in colon cancer, despite the suppressive cascade of Hippo signaling remaining operational. The protein stability of YAP is subject to a tiny balance between ubiquitination and deubiquitination processes. Through correlation analysis of DUBs (deubiquitinases) expression and Hippo target gene signature in colon cancer samples, we found JOSD1 as a critical deubiquitinase for Hippo signaling and colon cancer progression. JOSD1 could facilitate colon cancer progression and in colon cancer, inhibition of JOSD1 via shRNA has been demonstrated to impede tumorigenesis. Furthermore, molecular mechanism studies have elucidated that JOSD1 enhances the formation of the Hippo/YAP transcriptome by impeding K48-linked polyubiquitination on YAP. ChIP assays have shown that YAP binds to JOSD1’s promoter region, promoting its gene transcription. These results suggest that JOSD1 is involved in both activating and being targeted by the Hippo signaling pathway in colon cancer. Consequently, a positive regulatory loop between JOSD1 and Hippo signaling has been identified, underscoring their interdependence during colon cancer progression. Thus, targeting JOSD1 may represent a promising therapeutic approach for managing colon cancer.

## Introduction

Colon cancer ranks NO.3 in cancer incidence and NO.2 in cancer mortality, while the overall survival is still unsatisfying [1]. Several risk factors, such as obesity and high fat diet, are recognized to contribute to colon carcinogenesis [2]. Although regional colon cancer could be well treated by surgery, advanced colon cancer always accompanies with lymph node invasion and metastasis, while the 5-year survival is less than 40% [3, 4]. With the application of new target therapy and immune therapy, a few of the advanced colon cancer patients could benefit with prolonged survival time [5]. However, the effective therapeutic targets are still limited in colon cancer, it is urgent and necessary for cancer biologists to elucidate the driver pathways in colon cancer progression and identify novel therapeutic targets [6].

The Hippo pathway was originally identified from Drosophila, which played important role in the development of eye disc and wing [7, 8]. The Hippo signaling pathway involves a series of phosphorylated kinases such as MST1/2, LATS1/2, MAP4K, and NF2 When an upstream stimulus triggers Hippo signaling, the phosphorylated kinase MST1/2 induces phosphorylation of LATS1/2, which in turn phosphorylates YAP at multiple serine/threonine sites, preventing its nuclear localization [9–11]. This phosphorylates YAP at multiple serine/threonine sites and prevents its nuclear localization. The phosphorylated YAP protein interacts with certain E3 ubiquitin ligases (e.g. β-TrCP), leading to protein degradation [12, 13]. In contrast, when the Hippo pathway is inactive, unphosphorylated YAP proteins migrate to the nucleus, where they interact with transcription factors such as TEADs, inducing the expression of Hippo target genes and promoting carcinogenesis [14, 15]. Numerous studies have revealed abnormalities in the Hippo pathway in human malignancies, including colon cancer. For example, increased YAP protein expression was found in human colon cancer samples, and its expression was observed to be associated with local invasion and distant metastasis [16, 17]. Clinical survival statistics have shown that YAP expression is associated with poor survival in colon cancer [18, 19]. Various mechanistic studies have shown that YAP plays a crucial role in the development of colon cancer, and the growth and invasion of colon cancer cells can be inhibited by inhibiting YAP [20–22]. To date, targeting YAP is considered a valuable cancer treatment strategy based on human knowledge of Hippo signaling in colon cancer.

The uncontrolled activity of Hippo/YAP axis is a prominent character for colon cancer, while the inhibition phosphorylation cascade is still functional [23]. Recent studies pointed that Among them, the ubiquitination and deubiquitination process plays vital roles in regulation YAP protein stability and function. The human genome contains about one hundred deubiquitinating enzymes. It remains unclear which of these deubiquitinating enzymes can affect Hippo signaling and colon cancer growth. In our study, we aim to identify the key deubiquitinating enzymes that can regulate the Hippo pathway in colon cancer progression, which has important implications for colon cancer treatment.

## Results

### JOSD1 correlates with Hippo/YAP gene signature in colon cancer samples

**Fig. 1:**
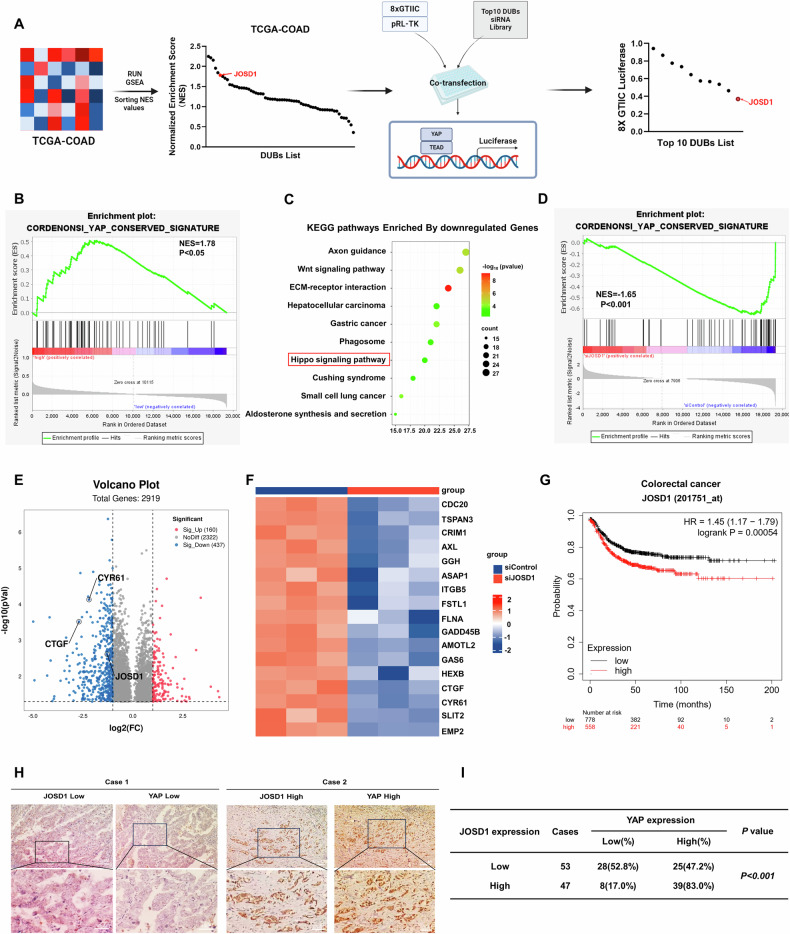
JOSD1 correlates with Hippo/YAP gene signature in colon cancer samples. **A** Ten DUBs strongly associated with the Hippo pathway were screened by GSEA, which were tested for 8×GTIIC luciferase reporter activity. **B** The data input in TCGA for Gene Set Enrichment Analysis (GSEA) indicated a highly significant positive correlation between JOSD1 and YAP target genes. **C** The top 10 KEGG pathways enriched by JOSD1 depletion in HCT116 cells were identified by RNA-seq data analysis, with a threshold of *P* < 0.05. **D** GSEA analysis revealed that the YAP target gene signature was downregulated upon JOSD1 depletion in HCT116 cells. **E** Volcano plots showed that JOSD1 depletion suppressed the expression of Hippo signaling genes (highlighted in red) in HCT116 cells. The threshold criteria for this analysis were set at *P* < 0.05 and fold change >1.5. **F** A heat map was generated to show the differentially expressed genes related to the Hippo signaling pathway whose expression was significantly decreased by siJOSD1 in HCT116 cells. **G** Kaplan-Meier analysis indicated a correlation between JOSD1 expression and lower progression-free survival among patients with colorectal cancer. **H**, **I** A positive correlation between JOSD1 and YAP was observed in immunohistochemical analysis of colorectal cancer samples (*P* < 0.001).

We firstly analyzed the whole genomic expression profiling of colon cancer samples from TCGA database. The correlation of expression of each DUB (deubiquitinase) with Hippo gene signature was analyzed. The GSEA (Gene signature enrichment analysis) harvested ten DUBs with highest NES (Normalized enrichment score). We further validated these 10 genes via siRNA on TEAD luciferase activity, in which JOSD1 depletion showed lowest TEAD luciferase activity in colon cancer cells (Fig. 1A). From the TCGA colon cancer samples, JOSD1 expression positively correlated with YAP target gene signature (NES = 1.75; *P* < 0.05; Fig. 1B). We further depleted JOSD1 in HCT116 cells and carried out RNA sequence analysis. The RNA sequencing data demonstrated that JOSD1 silencing induced dramatic change of Hippo signature (Fig. 1C), while JOSD1 depletion decreased the global YAP target gene expression from GSEA analysis (NES = 1.65; *P* < 0.001; Fig. 1D). Volcano map and heat-maps showed that a classical group of YAP target genes was reduced (Fig. 1E, F). Further survival analysis from KMPLOT database showed JOSD1 expression correlated with poor survival in colon cancer patients (Fig. 1G). In contrast, there was no correlation between the prognosis of other gastrointestinal tumors and JOSD1 expression, such as gastric cancer, esophageal cancer, and cholangiocarcinoma (Fig. S1A–D). However, we found no significant difference in JOSD1 expression between tumor tissues and adjacent normal tissues (Fig. S1E, F). We carried out IHC (Immuno-histochemistry) to investigate the expression of JOSD1 with clinical characteristics in colon cancer sample, in which JOSD1 expression correlated with tumor invasion, lymph node invasion and distant metastasis (Supplementary Table 1). Besides, the immunohistochemistry data show a positive relation between YAP expression and JOSD1 (*P* < 0.001; Fig. 1H, I).

### JOSD1 is an important modulator for colon cancer cell progression

**Fig. 2:**
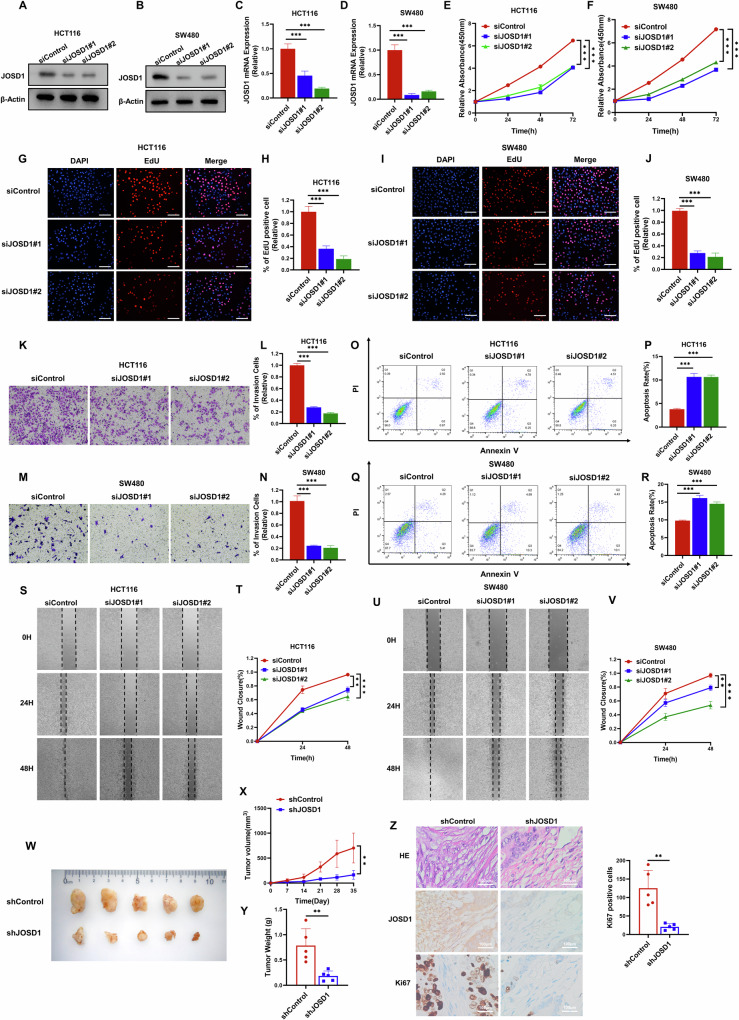
JOSD1 is an important modulator for colon cancer cell progression. **A**–**D** Immunoblot analysis and qRT–PCR were used to determine the expression level of JOSD1 in HCT116 and SW480 cells transfected with siControl or two independent siJOSD1. β-Actin was used as internal control. **E**, **F** The results showed that JOSD1 depletion inhibits the proliferation of colorectal cancer cells. Experiments were performed in triplicate. Statistical significance was observed for cell growth comparisons. **G**–**J** JOSD1 depletion resulted in a reduction in the number of EdU-positive colorectal cancer cells. **K**–**N** Depletion of endogenous JOSD1 significantly reduced cell invasion. **O**–**R** The depletion of JOSD1 promoted apoptosis in both HCT116 and SW480 cells. A quantitative summary of the apoptosis analysis was performed using FACS. **S**–**V** Depletion of JOSD1 inhibited the growth of colorectal tumors in vivo as demonstrated by the wound healing assay performed on HCT116 and SW480 cells with JOSD1 depletion or siControl transfection. **W**–**Y** The suppression of JOSD1 impacts xenograft tumor growth. **Z** The analysis of xenograft tumors revealed that depletion of JOSD1 led to reduced expression of Ki67, a marker of cell proliferation, in the tumors. The quantification of Ki67 positive cells showed a notable decrease in the right panel. All data are shown as mean ± SEM. *P < 0.05, **P < 0.01, ***P < 0.001 by one-way ANOVA.

We selected two colon cancer cell lines, HCT116 and SW480, and investigated the phenotype of JOSD1 in these cell lines. Endogenous JOSD1 was removed by transfecting siRNA, and knockdown efficiency was verified by western blot and qRT–PCR (Fig. 2A–D). The CCK8 assay demonstrated significant inhibition of colon cancer cell growth with JOSD1 deletion (Fig. 2E, F), while the EdU doping assay revealed a marked decrease in colon cancer cell proliferation due to JOSD1 deletion (Fig. 2G–J). The transwell experiments demonstrated that the invasive ability of colon cancer cells was significantly reduced upon removal of JOSD1 (Fig. 2K–N). The apoptosis assay revealed an increase in the number of apoptotic cells in HCT116 and SW480 cells upon JOSD1 knockdown (Fig. 2O–R). Due to the high proportion of early apoptotic cells indicated by the flow cytometry results, we chose to verify this using cleaved caspase-3 protein expression. As expected, when JOSD1 was knocked down, the level of cleaved caspase-3 increased (Fig. S2A, B). Furthermore, wound healing assays indicated that silencing JOSD1 impeded the migration of colon cancer cells (Fig. 2S–V). The function of JOSD1 was further assessed through an in vivo experiment using a xenograft tumor model. The results showed that silencing JOSD1 inhibited the tumorigenic potential of colon cancer cells (Fig. 2W–Y). Given the widespread use of 5-FU in the treatment of colon cancer, we knocked down JOSD1 and observed that HCT116 cells exhibited decreased drug resistance (Fig. S2C). In addition, subsequent immunohistochemical analysis of the xenograft tumors revealed reduced expression of Ki67 (Fig. 2Z). Furthermore, when JOSD1 was overexpressed, the extent of liver metastasis in mice was exacerbated, indicating that the metastatic potential of HCT116 cells was significantly increased in vivo (Fig. S2D, E). Therefore, we conclude that JOSD1 plays a crucial role in the progression of colorectal cancer cells.

### JOSD1 activates Hippo/YAP axis in colon cancer cells

**Fig. 3:**
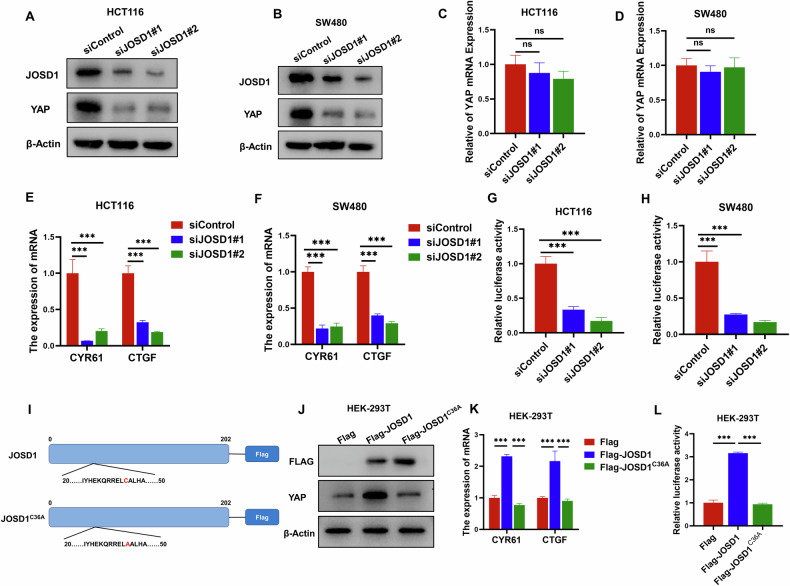
JOSD1 activates Hippo/YAP axis in colon cancer cells. **A** HCT116 cells were transfected with either siControl or siJOSD1 targeting sequences. Subsequently, after 48 h post-transfection, the cells were harvested for western blot analysis. Western blotting was then performed to assess the protein levels of JOSD1 and YAP, with actin utilized as the internal control throughout the experimental procedure. The results of these analyses revealed a reduction in YAP protein levels following depletion of JOSD1 in HCT116 cells. **B** Depletion of JOSD1 was observed to result in a reduction of YAP protein levels in SW480 cells. To determine protein levels, western blot analysis was performed on SW480 cells that were transfected with either siControl or siJOSD1 and harvested after 48 h. The levels of JOSD1 and YAP proteins were determined using western blotting, with β-Actin serving as the internal control. **C**, **D** The qRT–PCR analysis revealed that the depletion of JOSD1 reduces the stability of YAP protein, but not the expression of YAP mRNA. HCT116 and SW480 cells were transfected with 50 nM siControl or 50 nM JOSD1. **E** Depletion of JOSD1 reduced the expression of Hippo target genes in HCT116 cells. To analyze gene expression, HCT116 cells were transfected with siControl or siJOSD1 and total RNA was extracted after 48 h. Each group was tested in triplicate. **F** Depletion of JOSD1 reduced the expression of Hippo target genes in SW480 cells. To analyze gene expression, SW480 cells were transfected with siControl or siJOSD1 and total RNA was extracted after 48 h. Each group was tested in triplicate. **G** TEAD luciferase activity decreased in HCT116 cells when JOSD1 was depleted through transfection with siControl or siJOSD1 followed by TEAD luciferase reporter plasmid transfection. **H** TEAD luciferase activity in SW480 cells decreased following JOSD1 depletion. **I** Schematic diagram of the JOSD1 plasmid and its mutant JOSD1^C36A^ plasmid. **J** The overexpression of JOSD1 led to a marked increase in the expression of Hippo target genes, whereas the overexpression of JOSD1^C36A^ did not have the same effect. **K** The overexpression of JOSD1 resulted in an increase in the YAP protein level, whereas JOSD1^C36A^ overexpression did not exhibit the same effect. **L** In HEK293T cells, overexpression of JOSD1 increased TEAD luciferase activity, while JOSD1^C36A^ did not. All data are shown as mean ± SEM. *P < 0.05, **P < 0.01, ***P < 0.001 by one-way ANOVA.

YAP protein is known to play a crucial role in Hippo signaling activities [24], and our investigation into the impact of JOSD1 on YAP protein confirms this. We observed a decrease in the level of YAP protein upon deletion of JOSD1 in HCT116 and SW480 cells, while the mRNA level of YAP remained unaffected (Fig. 3A–D). The study clearly demonstrates that JOSD1 plays a crucial role in regulating YAP post-translationally. The qRT–PCR data strongly supports this finding, as silencing JOSD1 resulted in a significant reduction in the expression of YAP target genes, including CTGF and CYR61, in both HCT116 and SW480 cells (Fig. 3E, F). The TEAD-responsive element luciferase assay clearly shows that YAP activity decreases in HCT116 and SW480 cells when JOSD1 is depleted (Fig. 3G, H). Since the Hippo pathway is characterized by phosphorylation, we tested YAP phosphorylation and found that with JOSD1 knockdown, YAP protein expression decreased, and YAP phosphorylation levels also declined. We believe the reduction in YAP phosphorylation is due to the overall decrease in YAP expression, which is independent of JOSD1 knockdown (Fig. S3A, B). Considering the association between YAP and TAZ in the Hippo pathway, we also tested the TAZ protein and found that it was not affected (Fig. S3C, D). Since JOSD1 is a putative deubiquitinase, we wonder if its effect on YAP protein is dependent on the ubiquitin peptidase activity of JOSD1. We constructed the enzyme dead form of JOSD1 (JOSD1^C36A^). In western blot analysis, JOSD1 wild type form could enhance the expression of YAP target genes, such as CTGF and CYR61, while the JOSD1^C36A^ could not (Fig. 3I, J). In the gene expression assay, JOSD1 wild type form could increase YAP protein level, while the JOSD1^C36A^ could not (Fig. 3K). In the luciferase assay, the wild type form of JOSD1 could increase the TEAD luciferase activity, while the JOSD1^C36A^ could not (Fig. 3L). Furthermore, the transfection of either JOSD1 or JOSD1^C36A^ does not impact YAP phosphorylation (Fig. S3E). Thus, the JOSD1 could facilitate YAP function in colon cancer cells, which depended on its ubiquitin peptidase activity.

### JOSD1 facilitates colorectal cancer cell progression via Hippo/YAP axis

**Fig. 4:**
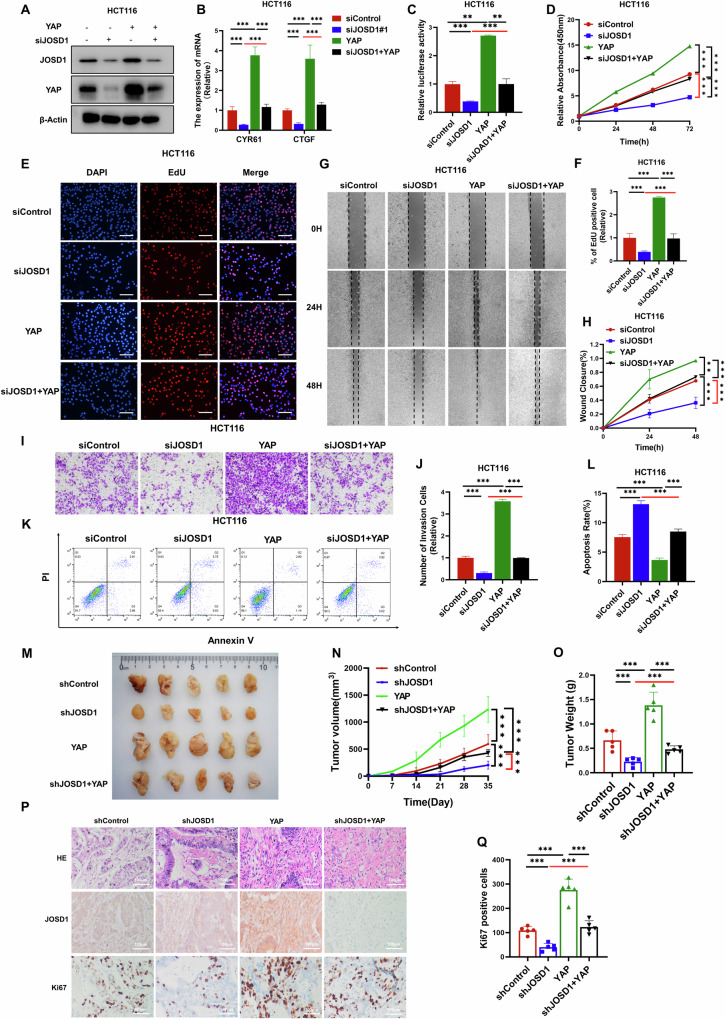
JOSD1 facilitates colon cell progression via Hippo/YAP axis. **A** Depletion of JOSD1 reduced the level of YAP protein, which was then restored by overexpressing YAP. HCT116 cells were transfected with either siControl or siJOSD1. They were then subjected to another round of transfection with Flag-YAP or Flag vector after 24 h. Subsequently, the cells were harvested for western blot analysis 48 h later to assess the expression levels of JOSD1 and YAP proteins, with actin serving as the internal control. **B** Depletion of JOSD1 suppressed the expression of Hippo target genes, which was reversed by YAP overexpression. HCT116 cells were transfected with either siControl or siJOSD1. Subsequently, they underwent a secondary transfection with Flag-YAP or Flag vector after 24 h. RNA extraction from experimental groups was performed using Trizol to assess the impact on the expression of YAP target genes. **C** Depletion of JOSD1 reduced TEAD luciferase activity in HCT116 cells. YAP overexpression reversed this effect. **D** The CCK-8 assay measured the cell growth of HCT116 cells. JOSD1 depletion inhibited their ability to proliferate. However, this effect could be reversed by overexpressing YAP. **E**, **F** Depletion of JOSD1 reduced the number of colorectal cancer cells that were positive for EdU. YAP overexpression further rescued this effect. Cell proliferation activity was indicated by the absolute cell number. **G**, **H** Depleting JOSD1 reduced the migratory ability of colorectal cancer cells. YAP overexpression reversed this effect. **I**, **J** Depleting JOSD1 reduced the invasive ability of colorectal cancer cells. YAP overexpression reversed this effect. The mean cell number was calculated and presented with standard deviations. **K**, **L** FACs were used to measure the apoptosis of HCT116 cells as indicated. Depletion of JOSD1 promoted apoptosis in HCT116 cells, and YAP overexpression was found to attenuate this effect. The right panel shows a quantitative summary of the apoptosis analysis using FACS. **M**–**O** In vivo, YAP overexpression rescued the growth of xenograft tumors from colon cancer cells transfected with siJOSD1. Panels (**M**), (**N**), and (**O**) show the tumor growth curves, weights, and photographs, respectively. **P**, **Q** The IHC staining of xenografts revealed the levels of JOSD1, YAP, and Ki67. The quantification of Ki67 positive cells results is shown in the right panel. All data are shown as mean ± SEM. *P < 0.05, **P < 0.01, ***P < 0.001 by one-way ANOVA.

To further investigate the relationship between the Hippo pathway in JOSD1 function and colon cancer, several rescue experiments were conducted following the aforementioned experiments. The efficiency of JOSD1 silencing and YAP overexpression was verified using western blot analysis (Figs. 4A and S4A). Subsequent qRT–PCR analysis revealed that depletion of JOSD1 led to the suppression of YAP target genes expression, which was rescued upon further YAP overexpression (Figs. 4B and S4B). The luciferase assay revealed that deleting JOSD1 decreased TEAD luciferase activity, but this effect was reversed by overexpressing YAP (Figs. 4C and S4C). This trend was also seen in the proliferation of colon cancer cells in the CCK8 assay (Figs. 4D and S4D). In the EdU incorporation assay, JOSD1 depletion led to a reduction in the number of proliferating cells. Notably, this decrease could be partially rescued by the overexpression of YAP (Figs. 4E, F and S4E, F). Likewise, findings from wound healing experiments suggested that the migration ability of colon cancer cells was significantly impeded upon JOSD1 deprivation. However, this inhibitory effect on cell migration could be partly restored by further overexpressing YAP (Figs. 4G, H and S4G, H). Moreover, results from the transwell experiment indicated that the invasive capacity of colon cancer cells was compromised upon JOSD1 exhaustion. Interestingly, the invasive ability of these cells could be partially rescued by additional YAP overexpression (Figs. 4I, J and S4I, J). In addition, in the FACS analysis, JOSD1 depletion was found to elevate cell apoptosis in colon cancer cells. It is noteworthy that the augmented apoptosis could be partially alleviated by further overexpression of YAP (Figs. 4K, L and S4K, L). This was further corroborated by the increased levels of cleaved caspase-3 (Fig. S5A). In addition, long-term clonogenesis experiments demonstrated that JOSD1 depletion inhibits colon cancer growth, and this inhibitory effect can be reversed by YAP overexpression (Fig. S5B, C). The xenograft mouse model further demonstrated that the inhibition of JOSD1 could effectively suppress colon tumor growth in vivo. Interestingly, the growth arrest induced by JOSD1 silencing could be partially rescued by the additional overexpression of YAP (Fig. 4M–O). Finally, IHC analysis revealed that JOSD1 depletion was associated with a decrease in the number of Ki67 positive cells in vivo. Notably, this effect could be reversed by further overexpressing YAP (Fig. 4P, Q). Therefore, JOSD1 controls the advancement of colorectal cancer cells via the Hippo/YAP pathway.

### JOSD1 is associated with YAP and regulates YAP protein stability

**Fig. 5:**
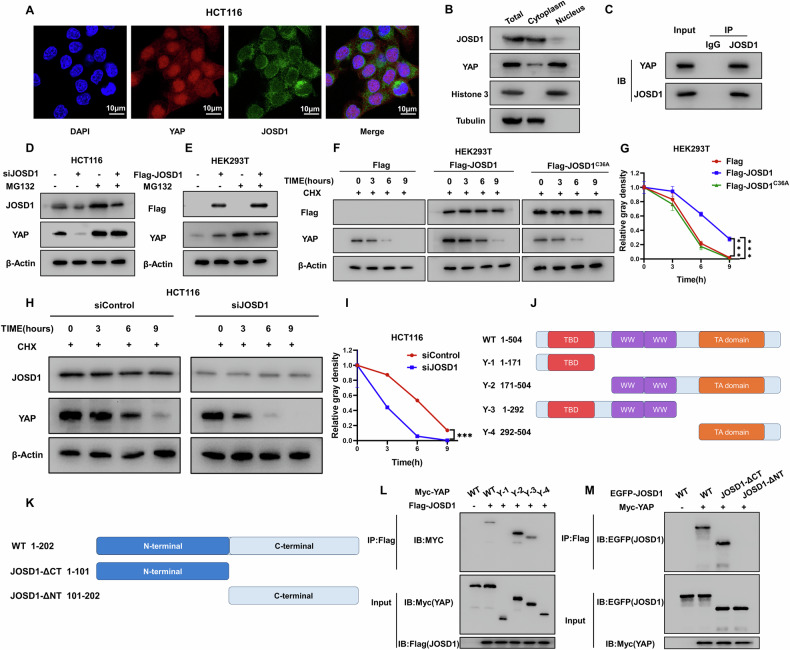
JOSD1 is associated with YAP and regulates YAP protein stability. **A** Immunofluorescence staining was used to analyze the intracellular localization of JOSD1 and YAP in HCT116 cells grown in standard medium. The findings show the localization of JOSD1 (green) and YAP (red), while nuclei are stained blue with DAPI. **B** In HCT116 cells, JOSD1 and YAP were primarily located in the nucleus. **C** In HCT116 cells, the Co-IP assay showed that endogenous JOSD1 and YAP are associated. An antibody was used for Co-IP. **D** Knockdown of JOSD1 does not result in further degradation of YAP in the presence of the proteasome inhibitor MG132. **E** When MG132, a proteasome inhibitor, was present, JOSD1’s stabilizing effect on YAP did not cause an additional increase in the YAP protein level. **F**, **G** In HEK293T cells, overexpressing JOSD1 extended the half-life of YAP, whereas overexpressing JOSD1^C36A^ did not. **H**, **I** Depletion of JOSD1 reduced the half-life of YAP protein in HCT116 cells. The halftime of YAP protein in (**I**) was quantitatively analyzed. **J**, **K** Schematic diagrams showing the wild-type and truncated YAP and JOSD1 constructs. **L**, **M** Immunoblots display the JOSD1 interaction with WT or truncated YAP through immunoprecipitation with JOSD1 (anti-Flag), and YAP interaction with WT or truncated JOSD1 via immunoprecipitation with YAP (anti-Myc). All data are shown as mean ± SEM. *P < 0.05, **P < 0.01, ***P < 0.001 by one-way ANOVA.

The study examined the localization of JOSD1 and YAP in colon cancer cells. Immunostaining assays indicated that JOSD1 mainly resided in the cytoplasm, whereas YAP was found in both the cytoplasm and the nucleus (Fig. 5A). This was also confirmed in the nucleus and cytoplasm separation assay (Fig. 5B). At the same time, assays confirmed that the knockout of JOSD1 did not affect the nucleocytoplasmic localization of YAP (Fig. S6A–F). Notably, the transfection of either JOSD1 or JOSD1^C36A^ also did not impact YAP nuclear localization (Fig. S6G–I). In addition, endogenous immunoprecipitation experiments demonstrated that JOSD1 could interact with YAP in colon cancer cells (Fig. 5C). After demonstrating the association between JOSD1 and YAP, we investigated its biological effect on YAP protein. We inhibited YAP degradation using the proteasome inhibitor MG132 and found that removal of JOSD1 decreased YAP protein levels in colon cancer cells. MG132 reduced the effect of JOSD1 removal on YAP protein levels (Fig. 5D, E). This effect was further confirmed by overexpression of JOSD1 in HEK293T cells. Furthermore, protein stability assays were conducted using the protein synthesis inhibitor cycloheximide. The results indicated that wild-type JOSD1 increased the stability of YAP, while catalytically defective JOSD1 did not (Fig. 5F, G). Deletion of JOSD1 in colon cancer cells resulted in a decrease in the half-life of YAP protein (Fig. 5H, I). YAP protein comprises of three functional domains: the TEAD-binding domain (TBD), the WW structural domain, and the transactivation structural domain (TA) (Fig. 5J). The N-terminal end of the JOSD1 protein contains a putative deubiquitinating enzyme, Josephine, in the structural domain (Fig. 5K). We conducted deletion construct experiments to study the interaction between YAP and JOSD1. Our findings show that the YAP’s WW structural domain is essential for its interaction with JOSD1. In addition, JOSD1 interacts with YAP through its N terminus (Fig. 5L, M).

### JOSD1 stabilizes YAP via inhibiting YAP K48-linked poly-ubiquitination

**Fig. 6:**
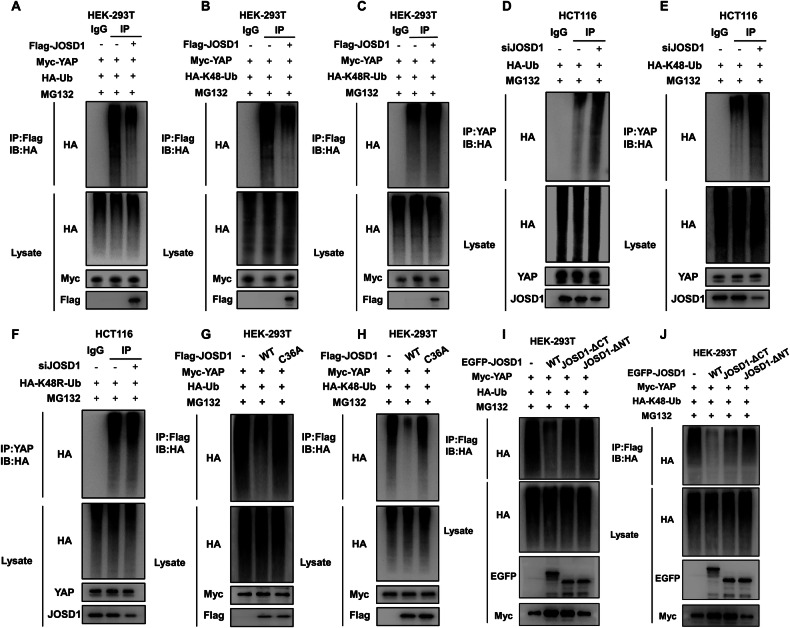
JOSD1 stabilizes YAP via inhibiting YAP K48-linked poly-ubiquitination. **A** JOSD1 decreased YAP polyubiquitination. After treating HEK-293T cells with MG132 for 6 h, they were transfected with 2 μg of YAP plasmid, 0.5 μg of HA-Ub plasmid, and either 0.5 μg of Flag-tag or Flag-JOSD1 plasmids. Subsequently, the cells were immunoblotted with specific antibodies. Polyubiquitination of YAP was reduced by JOSD1. **B**, **C** JOSD1 decreased YAP polyubiquitination. After treating HEK-293T cells with MG132 for 6 h, they were transfected with 2 μg of YAP plasmid, 0.5 μg of HA-K48-Ub or HA-K48R-Ub, and either 0.5 μg of Flag-tag or Flag-JOSD1 plasmids. Subsequently, the cells were immunoblotted with specific antibodies. YAP is deubiquitinated by JOSD1 through K48-linked polyubiquitination. **D** Reducing JOSD1 levels led to a higher amount of polyubiquitinated YAP. HCT116 cells were transfected with 0.5 μg HA-Ub plasmid and 20uM JOSD1 siRNA after treatment with MG132 for 6 h, followed by immunoblotting with specified antibodies. **E**, **F** JOSD1 deficiency increased YAP polyubiquitination related to K48, but not K48R. HCT116 cells were transfected with 0.5 μg HA-K48-Ub or HA-K48R-Ub plasmid and 20uM siJOSD1 after treatment with MG132 for 6 h, followed by immunoblotting with specified antibodies. **G**, **H** The JOSD1 mutant lacking deubiquitinating enzyme activity cannot enhance the accumulation of polyubiquitinated YAP. HEK-293T cells were transfected with 2 μg YAP plasmid, 0.5 μg HA-Ub/HA-K48 Ub plasmid and 0.5 μg Flag-tag or Flag-JOSD1 or Flag-JOSD1^C36A^ upon MG132 treatment for 6 h and then immunoblotted with the indicated antibodies. **I**, **J** JOSD1 deubiquitinates YAP via its N-terminal region. HEK-293T cells were transfected with 2 μg YAP plasmid, 0.5 μg HA-Ub/HA-K48 Ub plasmid, and 0.5 μg EGFP-tag or EGFP-JOSD1 full-length or deletion mutant plasmids after 6-h MG132 treatment. Subsequently, immunoblotting was performed using specified antibodies.

We investigated the role of JOSD1 in YAP ubiquitination in the HEK-293T model through immunoprecipitation experiments. The results from these experiments confirmed that JOSD1 inhibited the total ubiquitination level of YAP, particularly the K48-linked ubiquitination level (Fig. 6A–C). Subsequently, we delved deeper into the impact of JOSD1 on YAP polyubiquitination in HCT116 cells. Our investigation involved conducting endogenous Co-IP assays combined with ubiquitin signal transduction immunoblotting, which revealed that the total ubiquitination level of YAP, along with the K48-linked ubiquitination level, increased upon the knockdown of JOSD1 (Fig. 6D, E). Consistently, the dominant negative mutant of ubiquitin (K48R) diminished the effect of JOSD1 on YAP poly-ubiquitination (Fig. 6F). This outcome indicates that JOSD1 plays a specific role in inhibiting the K48-linked ubiquitination of YAP. To gain further insights into the underlying mechanism, we explored the regulatory effects of both wild-type JOSD1 and its enzyme-deficient mutant JOSD1^C36A^ on YAP polyubiquitination. Notably, our findings demonstrated that the mutant JOSD1^C36A^ attenuates the impact of JOSD1 on YAP ubiquitination (Fig. 6G, H). The structural domain-based ubiquitination assays showed that the N-terminal structural domain of JOSD1 is crucial for the deubiquitination of YAP by JOSD1 (Fig. 6I, J).

### YAP regulates the expression of JOSD1, forming a feedback loop between Hippo signaling and JOSD1

**Fig. 7:**
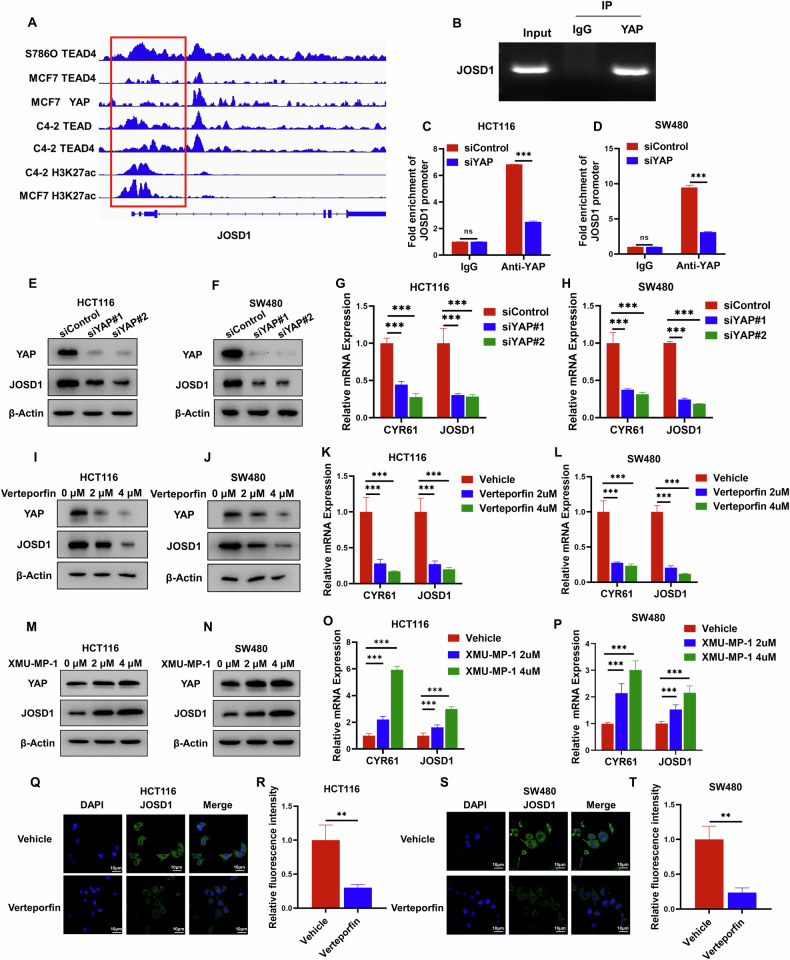
YAP regulates the expression of JOSD1, forming a forward regulatory loop in YAP signaling with JOSD1. **A** The JOSD1 genome schematic was analyzed, and the binding region of YAP to the JOSD1 promoter was studied. **B** The ChIP analysis showed that YAP binds to the JOSD1 promoter region. HCT116 cells were fixed for 30 min and lysed to purify the DNA, using rabbit IgG as a negative control. Primer sequences can be found in the Methods section. The enriched DNA fragments were then analyzed by DNA gel electrophoresis. **C**, **D** Cells from HCT116 and SW480 were treated with siRNA to delete YAP, followed by fixation, lysis, DNA enrichment, and ChIP-qPCR analysis. Results indicated a decrease in YAP binding to the JOSD1 gene. **E**, **F** Decreased YAP levels result in a reduction of JOSD1 protein. YAP was silenced with siRNA in HCT116 or SW480 cells, followed by cell lysis and protein extraction. Immunoblotting of cell lysates was performed using specified antibodies, with β-Actin serving as an internal control. **G**, **H** Depletion of YAP in HCT116 and SW480 cells inhibits JOSD1 mRNA. After transfection of HCT116 and SW480 cells with siControl or siYAP, total RNA was extracted for gene expression analysis. Relative CYR61 and JOSD1 mRNA levels were assessed by qRT–PCR in triplicate for each group. Statistical significance was determined by comparing the expression levels of the target genes. **I**, **J** The expression of JOSD1 protein was inhibited in HCT116 and SW480 cells following verteporfin treatment. Proteins were extracted from HCT116 and SW480 cells following treatment with vector and verteporfin, and immunoblotting was conducted with the specified antibodies. **K**, **L** Treating HCT116 and SW480 cells with verteporfin led to the inhibition of JOSD1 mRNA. HCT116 and SW480 cells were treated with either vehicle or VP, followed by total RNA extraction after 48 h for gene expression analysis. Each group underwent triplicate testing. **M**, **N** Treatment with XMU-MP-1 increased JOSD1 protein expression in HCT116 and SW480 cells. Proteins were extracted from HCT116 and SW480 cells following treatment with vector and XMU-MP-1, and immunoblotting was conducted with the specified antibodies. **O**, **P** Treatment with XMU-MP-1 in HCT116 and SW480 cells increased JOSD1 mRNA expression. HCT116 and SW480 cells were treated with either vehicle or XMU-MP-1, followed by total RNA extraction after 48 h for gene expression analysis. Each group underwent triplicate testing. **Q**–**T** YAP inhibition reduced JOSD1 expression in the cell membrane of HCT116 and SW480 cells treated with either vehicle or verteporfin. Endogenous JOSD1 was marked in green, and nuclei were stained with DAPI (blue); scale bar, 20 mm. The figure on the right shows the quantitative plots of the fluorescence intensities. All data are shown as mean ± SEM. *P < 0.05, **P < 0.01, ***P < 0.001 by one-way ANOVA.

As YAP plays a vital role in regulating tumor progression, we conducted multiple studies to investigate its global genomic binding in various cancer models. Our analysis of YAP-based ChIP sequencing data from several studies revealed significant binding peaks in the promoter region of JOSD1, suggesting a potential regulatory role of YAP in JOSD1 expression (Fig. 7A). To validate this regulatory interaction, Chip assays were conducted, demonstrating a clear association of YAP protein with the promoter region of the JOSD1 gene (Fig. 7B). Furthermore, subsequent experiments involving the depletion of YAP in colon cancer cells demonstrated a significant reduction in YAP binding to the JOSD1 promoter region, as evidenced by the ChIP assay results (Fig. 7C, D). Consistently, the reduction of YAP levels in colon cancer cells correlated with a notable decrease in both JOSD1 mRNA and protein levels. This sequence of events collectively highlights the potential regulatory role of YAP in modulating JOSD1 expression in colon cancer cells (Fig. 7E–H). Previous studies have shown that verteporfin inhibits YAP function. The levels of JOSD1 mRNA and protein in HCT116 and SW480 cells were reduced after verteporfin application (Fig. 7I–L). Conversely, JOSD1 mRNA and protein levels in HCT116 and SW480 cells were increased by activation of YAP by XMU-MP-1 (Fig. 7M–P). This was confirmed by immunostaining (Fig. 7Q–T). This discovery unveiled a positive feedback pathway linking JOSD1 and YAP.

## Discussion

**Fig. 8:**
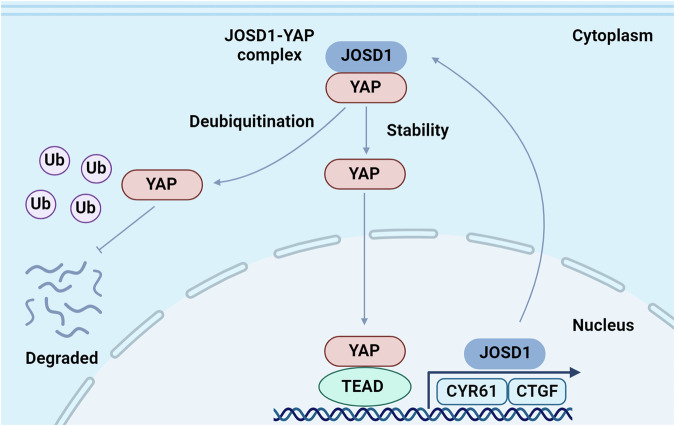
In colorectal cancer, JOSD1 forms a regulatory loop with the Hippo/YAP axis. This relationship is hypothesized to involve JOSD1 regulating YAP signaling and establishing a positive feedback loop. Activation of JOSD1 is believed to advance colorectal cancer progression through its modulation of YAP K48-linked deubiquitinating, resulting in increased YAP activity. Furthermore, YAP has been shown to stimulate the expression of JOSD1, thereby establishing a mutual reinforcement between the two factors.

Our study concludes that JOSD1 is associated with a genetic signature of Hippo signaling and correlates with poor survival in colon cancer. It was observed that JOSD1 facilitates the progression of colon cancer by modulating the deubiquitination of the YAP K48 linkage, thereby amplifying Hippo/YAP activity. Moreover, the study revealed that YAP upregulates JOSD1 expression, indicating a mutual reinforcement between JOSD1 and Hippo signaling in a positive feedback loop (Fig. 8). These findings highlight JOSD1 as a potential therapeutic target for colorectal cancer treatment, shedding light on a non-genomic regulatory mechanism of Hippo signaling through a newly discovered positive feedback loop.

JOSD1 (Josephin Domain Containing 1) is a putative deubiquitinase, which belongs to MJD family (Machado-Josephin domain-containing proteases) [25–27]. The link of Josephin family DUBs to human disease were firstly discovered in neurodegenerative diseases named Machado-Joseph disease, in which ATXN3 another MJD member decreased its expression and promoted the progression of Machado-Joseph disease [28, 29]. Although JOSD shared high similarity in amino acid sequence, there is currently no evidence to show its link to neurodegenerative diseases. However, recently studies emphasized the role of JOSD1 in human malignancies. For example, the gene amplification of JOSD1 was observed in uterine cancer and melanoma [30]. JOSD1 was reported to stabilize MCL1 and inhibit mitochondrial-dependent apoptosis, which subsequently promoted chemotherapy resistance in leukemia [31]. Further research proposed that a small molecule target JOSD1 could a promising strategy in JAK2-mutant leukemia patients [32]. In solid tumors, JOSD1 was found to stabilize Snail and promote lung cancer progression [33]. However, how JOSD1 functions, particularly in gastrointestinal malignancies, remains to be elucidated. Our study uncovers novel regulatory mechanisms by which JOSD1 regulates the Hippo pathway in colon cancer. We present key regulatory elements of JOSD1 within the Hippo pathway, shedding light on the interplay between the DUB family and the Hippo pathway.

The connection between Hippo signaling and colon cancer has been established for decades [34]. Targeting YAP function shows promise in colon cancer therapy [35]. Previous studies have shown that inhibiting the interaction of YAP with TEAD is a viable strategy for treating Hippo-driven cancers [36, 37]. However, drugs that inhibit the Hippo/YAP pathway, such as verteporfin and Super-TDU, have not been successful in clinical trials for Hippo-driven cancers [38]. One of the main reasons for this failure is the inability of the inhibitors to penetrate the cell membrane and block cell membrane protein interactions. In addition, the small peptide Super-TDU may be attacked by plasma proteases and neutralizing antibodies [39]. Due to the limitations of YAP-targeted drugs, our focus has shifted towards developing novel drugs that target the stability of the YAP protein in colon cancer. Considering the significance of JOSD1 in colon cancer, blocking JOSD1 may be a viable strategy for treating colon cancer.

In summary, this study identified forward feedback between the Hippo pathway and JOSD1 that drives colorectal cancer progression. Blocking JOSD1 and impeding the positive cycle may be a promising strategy for colon cancer treatment. Blocking YAP/TEAD interactions may be challenging in cancer therapy. Therefore, reactivating the Hippo pathway in colon cancer by targeting YAP protein stability may be feasible.

## Materials and methods

### Cell lines and cell culture

Human cell lines HCT116, SW480, and HEK-293T were procured from the American Type Culture Collection (ATCC). To validate the cell lines, we performed short tandem repeat (STR) analysis. The cells were cultured in Dulbecco’s modified Eagle’s medium (DMEM, Gibco, 21063029) supplemented with 10% Fetal Bovine Serum (FBS, Gibco, 12676-029), 1% Penicillin-Streptomycin-Gentamycin solution, 4.5 g/L glucose, and 4 mM L-Glutamine. In addition, 10% Fetal Bovine Serum (FBS), 4.5 g/L glucose, and 4 mM L-glutamine were added to the culture medium for optimal cell growth and maintenance.

### RNA isolation and quantitative real-time PCR (qRT–PCR)

Total RNA was extracted using the RNeasy Plus Mini Kit (Tiangen, DP451) following the manufacturer’s protocol, and reverse transcription was subsequently performed using HiScript II Q RT SuperMix (Vazyme, R223-01). The qRT–PCR analysis was then carried out using SYBR qRT-PCR Master Mix (Vazyme, Q511-02) on a 7500 Fast Real-Time PCR System (Applied Biosystems, Singapore). To normalize the gene expression levels, 36B4 was employed as an internal control. The primers used for qRT–PCR were as follows: 36B4 (Forward: GGC GAC CTG GAA GTC CAA CT; Reverse: CCA TCA GCA CAC AGC CTT C), CTGF (Forward: CTC GCG GCT TAC CGA CTG; Reverse: GGC TCT GCT TCT CTA GCC TG), CYR61 (Forward: GGT CAA AGT TAC CGG GCA GT; Reverse: GGA GGC ATC GAA TCC CAG C), JOSD1 (Forward: GGG ATA CGC TGC AAG AGA TTT; Reverse: CCA TGA CGT TAG TGA GGG CA), and YAP (Forward: CAA GAA AGC AGG CTC ACA GAA; Reverse: GCT GGG TGT TAG GGC TTC G).

### Plasmids and siRNA

Plasmids JOSD1 and YAP, sourced from HANBIO (https://www.Han-bio.net), were subjected to further subcloning from full-length plasmid DNA to create the deletion constructs. In addition, in this study, the well-established HA-K48 and HA-Ub plasmids were integrated. Plasmid transfection was carried out utilizing Lipofectamine 2000 (1,662,298, Invitrogen). Following this, specific genes were targeted for knockout through small interfering RNA (siRNA) technology. For JOSD1, the siRNA sequences used in the knockdown process were as follows: (1) CAA GGC CAA AUC UGA AUC and UGA UUC AGA UUU GGC CUU; and (2) CUA ACG UCA UGG GCU UCA and AUGAAGCCCAUGACGUUA. The YAP siRNA sequences utilized were: (1) GUC AGA GAU ACU UCU UAA and UUU AAG AAG UAU CUC UGA; and (2) GUC UCA GGA AUU GAG AAC and UGU UCU CAA UUC CUG AGA. Corresponding negative control siRNA sequences were employed: UUC UCC GAA CGU GUC ACG and ACG UGA CAC GUU CGG AGA. The lentivirus expressing JOSD1 shRNA was obtained by inserting shJOSD1 into the pLKO.1 vector and co-transfecting HEK293T cells with the pMD2.G envelope plasmid and the psPAX2 packaging plasmid. 48 h later, the lentiviral suspension was used to culture colon cancer cells in antibiotic-free medium for subsequent experiments.

### Reagents

The reagents used included XMU-MP-1 (MCE, Cat. No. HY-100526), CELLSAVING (New Cell & Molecular Biotech, C40100/C40050), MG132 (MCE, HY-13259), cycloheximide (MCE, Cat. No. HY-12320), and verteporfin (MCE, Cat. No. HY-B0146).

### Western blotting

After harvesting the cells, they were lysed with RIPA buffer (Beyotime, China) containing protease and phosphatase inhibitors. Following lysis, equal amounts of proteins were quantified and then subjected to separation by 10% SDS-PAGE, followed by transfer to PVDF membranes (Millipore, USA). The membrane was subsequently blocked with 5% nonfat milk for 1 h, and this was followed by incubation with the primary antibody overnight at 4 °C. After three washes with TBST, the membrane was subjected to detection using an ECL system (Bio-rad ChemiDoc), with images captured using a Tanon 5200 system (China). The internal control used in this analysis was β-actin. The primary antibodies employed were anti-Flag antibody (#F1804, Sigma, 1:1000), anti-JOSD1 antibody (ab118221, Abcam, 1:1000), anti-tubulin (11224-1-AP, Proteintech, 1:1000), anti-histone H3 (17168-1-AP, Proteintech, 1:1000), Myc antibody (#20002, Abmart, 1:1000), YAP antibody (1:500, Cell Signaling Techonology, #SC101199), Cleaved Caspase-3 (#9661, Cell Signalling, 1:2000), and anti-actin antibody (3700, Cell Signalling Technology, 1:1000). Subsequently, peroxidase-conjugated AffiniPure goat anti-mouse IgG (#A0216, Beyotime, 1:2000) or goat anti-rabbit IgG (#A0208, Beyotime, 1:2000) were employed for secondary antibody detection. The color development was achieved utilizing an ECL kit (Meilunbio, #MA0186)

### Luciferase reporter assays

Following the culturing of HCT116 or SW480 cell lines, the cells were simultaneously transfected with the luciferase reporter plasmid, Renila expression plasmid, and specific plasmids using Lipo 2000. Subsequently, after a 48-h incubation period, the cells were lysed to evaluate Hippo signaling activity.

### Wound healing and Transwell assays

The wound healing assay involved seeding of HCT116 and SW480 cells transfected with siJOSD1 or siControl into 6-well plates. Once the cells reached full growth, a wound was created by scratching the cell monolayer with a sterile tip. Following the injury, cells were imaged at a predetermined time point. Subsequently, the distance between the edges of the scratched wound was quantified using ImageJ software for analysis. To evaluate cell invasion, the transwell system with an 8 μm pore size from Corning was utilized. In invasion assays, Matrigel from BD Biocoat, USA was applied to coat the membranes in the upper chambers. Following a 24-h incubation period, the number of colorectal cancer cells that had invaded to the lower surface of the insert membrane was determined under a ×20 objective after fixing and staining with crystal violet. Notably, all experiments were conducted in triplicate to ensure the reliability of the results.

### Cycloheximide assay

After transfection with siJOSD1 or siControl for 24 h, HCT-116 cells were treated with 100 µmol/L cycloheximide. The same experimental protocol was followed with HEK293T cells, which were transfected with 2 μg of Flag-JOSD1 or Flag vector. Subsequently, cell lysates were obtained at specific time points (0, 3, 6, and 9 h post-treatment) to evaluate the effects of the interventions on both cell lines.

### Immunofluorescence (IF) staining

After inoculating colon cancer cells into 12-well plates, small molecule reagents were used for treatment. Following a 24-h incubation period, the cells were transferred to small dialysis slides. Cells were fixed by adding a 4% paraformaldehyde solution dropwise. They were then stained with primary antibodies against JOSD1 (1:100, Sigma-Aldrich, HPA001168) and YAP (1:200,14074, Santa Cruz, sc-271134) for two hours at room temperature. Subsequently, the cells were rinsed with PBS and incubated with a fluorophore-conjugated secondary antibody from Invitrogen (Carlsbad, CA), followed by another PBS rinse. Cell nuclei were stained with DAPI (Life Technology), and an anti-quenching reagent was added post-washing. Images of cell-specific proteins were captured using a laser scanning confocal microscope (Leica TCS SP8 STED) and then processed with ImageJ software for further analysis.

### Co-IP assay

To obtain a protein sample, cells are first lysed, resulting in a cell lysate. The decoy protein is then introduced into the cell lysate. Subsequently, a magnetic bead-coupled antibody is added to the sample, specifically binding to the decoy protein. By using a magnet, the bead together with the precipitated decoy protein is isolated. The next step involves separating the protein complexes through SDS-PAGE, followed by Western blotting (WB) to identify the target proteins within the sample.

### In vitro ubiquitination assays

HEK293T cells were transfected with Ub, K48 Ubi plasmid, K48R Ubi plasmid, Flag-JOSD1 plasmid, and Myc-YAP or vectors and incubated for 6 h. Subsequently, 20 µM MG132 (MCE, HY-13259) was introduced to the cells. Following this, total protein extraction was conducted, and IgG along with 30 µL protein A + G agarose (Beyotime, P2055) was added and incubated for 2 h. Anti-FLAG antibody was then introduced for immunoprecipitation. The polyubiquitination of YAP was analyzed through Western blotting by employing anti-HA antibody.

### In vivo tumorigenesis assay

For the in vivo tumorigenesis assay, 5-week-old female BALB/c nude mice were obtained from SPF (Beijing) Biotechnology Company. HCT116 cells were infected with either shControl or shJOSD1 lentivirus, followed by treatment with 1 μg/ml puromycin for 3 days after 48 h of infection. Subsequently, HCT116 cells (2 × 10^6^) were injected into the right back of the 5-week-old female BALB/c nude mice. Tumor formation in the nude mice was monitored over a period of 4 weeks, and tumor volumes were calculated using the formula tumor volume = 1/2 × length × width^2^. After five weeks, the mice were sacrificed, and the tumors were weighed and photographed.

### In vivo metastasis assays

To investigate the impact of JOSD1 on HCT116 metastatic potential in vivo, 5-week-old female BALB/c nude mice were obtained from SPF (Beijing) Biotechnology Company. HCT116 cells were infected with either Vehicle or Flag-JOSD1 lentivirus, followed by treatment with 1 μg/ml puromycin for 3 days after 48 h of infection. Subsequently, cells from each group were intravenously injected into the mice via the tail vein, with each mouse receiving 1 × 10^6^ cells. After six weeks, mice were euthanized, and the number of liver metastases was quantified.

### Cell proliferation assay

HCT116 and SW480 cells were transfected with siJOSD1 or siControl in 24-well plates and counted. 24 h after transfection, 4000 cells were seeded in 96-well plates. Relative cell viability was measured at specified time points by using the CCK8 cell proliferation reagent to determine the cell number through absorbance readings at 450 nm. EdU incorporation was used to further analyze cell proliferation. CRC cell counts were determined using a 5-ethynyl-20-deoxyuridine (EdU) assay kit (RiboBio, Guangzhou, China). A fluorescence microscope was then used to capture images.

### Quantification of cell viability

HCT116 cells were transfected with siJOSD1 or siControl. After 24 h, cells were re-digested, centrifuged, and seeded at a density of 4000 cells per well in a 96-well plate. Cells were then stimulated with varying concentrations of 5-FU. Cell viability was determined using the CCK-8 assay, measuring absorbance at 450 nm.

### Apoptosis assay

Cells transfected with siRNA or plasmids were incubated for 24 or 48 h, then stained with propidium iodide (PI) and annexin V. Subsequently, the fluorescence intensity was measured with a CytoFLEX flow cytometer.

### Immunohistochemistry (IHC)

The tissues were initially fixed in 4% paraformaldehyde, followed by paraffin embedding. Next, the tissues were sectioned with a semi-automatic paraffin microtome S700 (RWD, USA). Subsequent to sectioning, immunohistochemical (IHC) staining was carried out using an IHC kit (Zsbio, Beijing, China). The staining process comprised the incubation of primary and secondary antibodies, with the immune complexes visualized using DAB. Concurrently, the nuclei were counterstained with hematoxylin. Following staining, images were captured employing a NanoZoomer digital pathology scanner (NanoZoomer S60, HAMAMATSU, Japan). The acquired images were then evaluated based on integrated optical density (IOD) utilizing ImageJ software.

### RNA sequence and data analysis

Three biological replicates, each containing an equal amount of RNA, were prepared for the study. The samples were then sent to Novogene in Beijing, China, for the construction and analysis of RNA-seq libraries. After the analysis, a gene was considered to be significantly differentially expressed if it met two criteria: the log2 (fold change) of the gene was greater than 1 and the False Discovery Rate (FDR) was less than 0.05. Subsequently, KEGG pathway enrichment analysis was conducted using the cluster Profiler software package in R. The RNA sequence data resulting from the analysis were subsequently archived in the Gene Expression Omnibus (GEO) database under the accession number GSE256276. In addition, Genome enrichment analysis (GSEA) was performed utilizing the GSEA software available at http://www.broadinstitute.org/gsea to further explore the biological implications of the results.

### Chromatin immunoprecipitation (ChIP) assay

After 30 min of fixation, terminate with the introduction of glycine solution. Subsequently, the cells were washed with pre-cooled PBS containing PMSF, scraped, and centrifuged. Following centrifugation, the resulting cell mass was treated with SDS lysis buffer and sonicated for 10 min with on/off cycling for 30 s to break up the chromatin. The ChIP procedure was carried out using the ChIP Assay Kit (Millipore, 17-295), with a rabbit anti-YAP antibody (#SC101199) utilized in these experiments. The DNA was extracted following ChIP and analyzed by quantitative PCR using the DNA Extraction Kit (Qiagen, order #28106). Notably, the specific primers used were JOSD1 F: CTC TCG CGA TAG CTT CCT GG and R: TCC CTT TCC CTT GTG TGA CC.

### TCGA data and analysis of survival data

The TCGA database offers gene expression data on colorectal cancer patients. GEPIA online software was utilized to examine the correlation between JOSD1 expression and colorectal cancer patient survival. The results were generated using default parameters in GSEA online software. Volcano plots with a threshold of *P* < 0.05 and equivalent change >1.5 were generated using ‘ggplot2’ package in R.

### Statistical analysis

GraphPad Prism 8.0.1 (GraphPad, USA) was used for statistical analyses. To compare two groups, we used Student’s t-test, and for multiple comparisons, we used one-way analysis of variance (ANOVA). The χ^2^ test was used for categorical variables. Pearson’s correlation analysis was used to determine the correlation between measurements. The mean ± standard deviation (SD) of the measurement data is presented. A significant difference was considered when *P* < 0.05.

### Supplementary information

Supplementary Table 1

Supplementary Figure Legend

Supplementary Figure 1

Supplementary Figure 2

Supplementary Figure 3

Supplementary Figure 4

Supplementary Figure 5

Supplementary Figure 6

origin data

## Data Availability

RNA sequence data can be found in the GEO database (GSE256276). The original digital data and WB data are provided in supplementary materials.
