# Supplementary material for: Regulation of Hippo/YAP axis in colon cancer progression by the deubiquitinase JOSD1
Source: Cell Death Discov. 2024 Aug 14;10:365. doi: 10.1038/s41420-024-02136-7 (PMC11325045; doi:10.1038/s41420-024-02136-7)
Supplement: Supplementary file 1 — Supplementary Table 1 [file 41420_2024_2136_MOESM1_ESM.docx]

| **Clinical and molecular characteristics** | **Cases** | **JOSD1 expression** | | ***P* value** |
| --- | --- | --- | --- | --- |
|  |  | **Low group (%)** | **High group (%)** |  |
| **Age (years old)** |  |  |  |  |
| **≤60** | **26** | **17(65.4%)** | **9(34.6%)** | **0.4145** |
| **>60** | **59** | **33(55.9%)** | **26(44.1%)** |  |
| **Gender** |  |  |  |  |
| **Male** | **52** | **31(59.6%)** | **21(40.4%)** | **0.8523** |
| **Female** | **33** | **19(57.6%)** | **14(42.4%)** |  |
| **Tumor invasion** |  |  |  |  |
| **T1** | **3** | **3(100.0%)** | **0(0.0%)** | **0.006** |
| **T2** | **14** | **11(78.6%)** | **3(27.3%)** |  |
| **T3** | **41** | **27(65.9%)** | **14(34.1%)** |  |
| **T4** | **27** | **9(33.3%)** | **18(66.7%)** |  |
| **Distant metastasis** |  |  |  |  |
| **M0** | **74** | **49(66.2%)** | **25(33.8%)** | **0.0003** |
| **M1** | **11** | **1(9.1%)** | **10(90.9%)** |  |
| **Lymph node metastasis** |  |  |  |  |
| **N0** | **42** | **28(66.7%)** | **14(33.3%)** | **0.0391** |
| **N1** | **29** | **18(62.1%)** | **11(37.9%)** |  |
| **N2** | **14** | **4(28.6%)** | **10(71.4%)** |  |
| **Clinical stage** |  |  |  |  |
| **Early(Ⅰ-Ⅱ)** | **39** | **28(71.8%)** | **11(28.2%)** | **0.0253** |
| **Advanced(Ⅲ -Ⅳ)** | **46** | **22(47.8%)** | **24(25.2%)** |  |

**Statistical significance(*P*<0.05) is shown in bold**
