# Supplementary material for: Regulation of Hippo/YAP axis in colon cancer progression by the deubiquitinase JOSD1
Source: Cell Death Discov. 2024 Aug 14;10:365. doi: 10.1038/s41420-024-02136-7 (PMC11325045; doi:10.1038/s41420-024-02136-7)
Supplement: Supplementary file 2 — Supplementary Figure Legend [file 41420_2024_2136_MOESM2_ESM.docx]

**Supplementary Table 1:** **Clinicopathological correlation of JOSD1 expression in CRC.**

**Supplementary Figure Legends**

**Supplementary Figure 1. JOSD1 is not associated with the prognosis of other gastrointestinal tumors.**

**A-D**: The Kaplan-Meier analysis revealed no correlation between JOSD1 expression and reduced progression-free survival in patients diagnosed with esophageal cancer, gastric cancer, and cholangiocarcinoma.

**E**: We analyzed the differential expression of JOSD1 between tumor and normal tissues using data from the TCGA database and the GEPIA website. [GEPIA (Gene Expression Profiling Interactive Analysis) (cancer-pku.cn)](http://gepia.cancer-pku.cn/index.html)

All data are shown as mean ± SEM. **P* < 0.05, ***P* < 0.01, ****P* < 0.001 by one-way ANOVA

**Supplementary Figure 2. Role of JOSD1 in Colon Cancer Progression**

**A-B:** HCT116 and SW480 cells were transfected with siJOSD1 for 24 hours, lysed with IP lysate, centrifuged, and proteins were extracted for Western blotting experiments.

**C:** Downregulation of JOSD1 reduces resistance in HCT116 cells. Cells were treated with 50 nM siJOSD1 or siControl for 24 hours, followed by exposure to specified concentrations of 5-Fluorouracil. Cell viability was assessed using the CCK8 assay.

**D-E:** Overexpression of JOSD1 promotes in vivo metastatic potential of HCT116 cells. Stable clones overexpressing JOSD1 and empty vector controls were intravenously injected into nude mice. After six weeks, liver metastases were quantified.

All data are shown as mean ± SEM. **P* < 0.05, ***P* < 0.01, ****P* < 0.001 by one-way ANOVA

**Supplementary Figure 3. Impact of JOSD1 on YAP/TAZ Phosphorylation and Nuclear Localization**

**A-B:** Knockdown of JOSD1 affects YAP stability without altering YAP phosphorylation. HCT116 and SW480 cells were transfected with siJOSD1 or siControl for 24 hours, followed by sonication of lysates and analysis by immunoblotting.

**C-D:** HCT116 and SW480 cells were transfected with siJOSD1 or siControl for 24 hours, and cell lysates were prepared using cell lysis buffer. Immunoblotting was performed to assess the impact of JOSD1 knockdown on TAZ expression.

**E:** Overexpression of JOSD1 does not affect YAP phosphorylation, while JOSD1 mutants show distinct patterns. HEK293T cells were transfected with Flag, Flag-JOSD1, or Flag-JOSD1C36A, followed by immunoblotting with β-Actin as the internal reference protein.

All data are shown as mean ± SEM. **P* < 0.05, ***P* < 0.01, ****P* < 0.001 by one-way ANOVA

**Supplementary Fig. 4** **JOSD1 Facilitates Colon Cell Progression via the Hippo/YAP Axis**

**A:** Depletion of JOSD1 reduced YAP protein levels, which were restored by overexpressing YAP. SW480 cells were transfected with either siControl or siJOSD1, followed by transfection with Flag-YAP or Flag vector after 24 hours. Cells were harvested 48 hours later for Western blot analysis of JOSD1 and YAP protein expression, with actin as the internal control.

**B:** Depletion of JOSD1 suppressed the expression of Hippo target genes, which was reversed by YAP overexpression. SW480 cells were transfected with either siControl or siJOSD1. Subsequently, they underwent a secondary transfection with Flag-YAP or Flag vector after 24 hours. RNA extraction from experimental groups was performed using Trizol to assess the impact on the expression of YAP target genes.

**C:** Depletion of JOSD1 reduced TEAD luciferase activity in SW480 cells, which was restored by YAP overexpression.

**D:** The CCK-8 assay measured SW480 cell growth. Depletion of JOSD1 inhibited proliferation, which was rescued by YAP overexpression.

**E-F:** Depletion of JOSD1 reduced the number of EdU-positive colorectal cancer cells. YAP overexpression further rescued this effect, indicating cell proliferation activity.

**G-H:** Depletion of JOSD1 reduced the migratory ability of colorectal cancer cells, which was reversed by YAP overexpression.

**I-J:** Depletion of JOSD1 reduced the invasive ability of colorectal cancer cells, which was rescued by YAP overexpression. Mean cell numbers were calculated and presented with standard deviations.

**K-L:** FACS analysis measured apoptosis in SW480 cells. Depletion of JOSD1 promoted apoptosis, which was attenuated by YAP overexpression. The right panel shows quantitative summary of apoptosis analysis using FACS.

All data are shown as mean ± SEM. **P* < 0.05, ***P* < 0.01, ****P* < 0.001 by one-way ANOVA.

**Supplementary Fig. 5 JOSD1 Facilitates Colon Cell Progression via the Hippo/YAP Axis**

**A:** Depletion of JOSD1 promoted cell apoptosis, which was reversed by YAP overexpression. HCT116 cells were transfected with siJOSD1 for 24 hours, followed by IP lysate preparation, centrifugation, protein extraction, Then, immunoblotting analysis was conducted to assess whether Cleaved-Caspase 3 was affected.

**B-C:** Depletion of JOSD1 decreased clonogenesis in HCT116 cells, which was rescued by YAP plasmid transfection. shJOSD1 or shControl and YAP were introduced into HCT116 cells, and cell clones were counted after two weeks of culture.

**Supplementary Fig. 6 JOSD1 expression does not affect the nuclear localization of YAP**

**A-B:** Following transfection with siJOSD1 or siControl for 24 hours, nuclear and cytosolic proteins were isolated using the Nucleoplasm Isolation Kit. Immunoblotting confirmed that JOSD1 knockdown did not affect YAP nuclear localization.

**C-F:** Cells were transfected with siJOSD1 or siControl for 24 hours, followed by immunofluorescence analysis to assess YAP nuclear localization. Red fluorescence indicates YAP and blue fluorescence indicates DAPI staining. JOSD1 knockdown did not alter YAP nuclear localization.

**G:** Overexpression of JOSD1 did not alter its nuclear localization. HEK293T cells were transfected with Flag, Flag-JOSD1, or Flag-JOSD1C36A for 24 hours, and immunoblotting confirmed the localization of YAP.

**H-I:** HEK293T cells transfected with Flag, Flag-JOSD1, or Flag-JOSD1C36A were subjected to immunofluorescence analysis after 24 hours to assess YAP nuclear localization. The analysis showed that JOSD1 overexpression did not affect YAP nuclear localization. YAP was visualized in red fluorescence, while DAPI stained nuclei in blue fluorescence.
