# Supplementary figures and images for: Regulation of Hippo/YAP axis in colon cancer progression by the deubiquitinase JOSD1
Source: Cell Death Discov. 2024 Aug 14;10:365. doi: 10.1038/s41420-024-02136-7 (PMC11325045; doi:10.1038/s41420-024-02136-7)

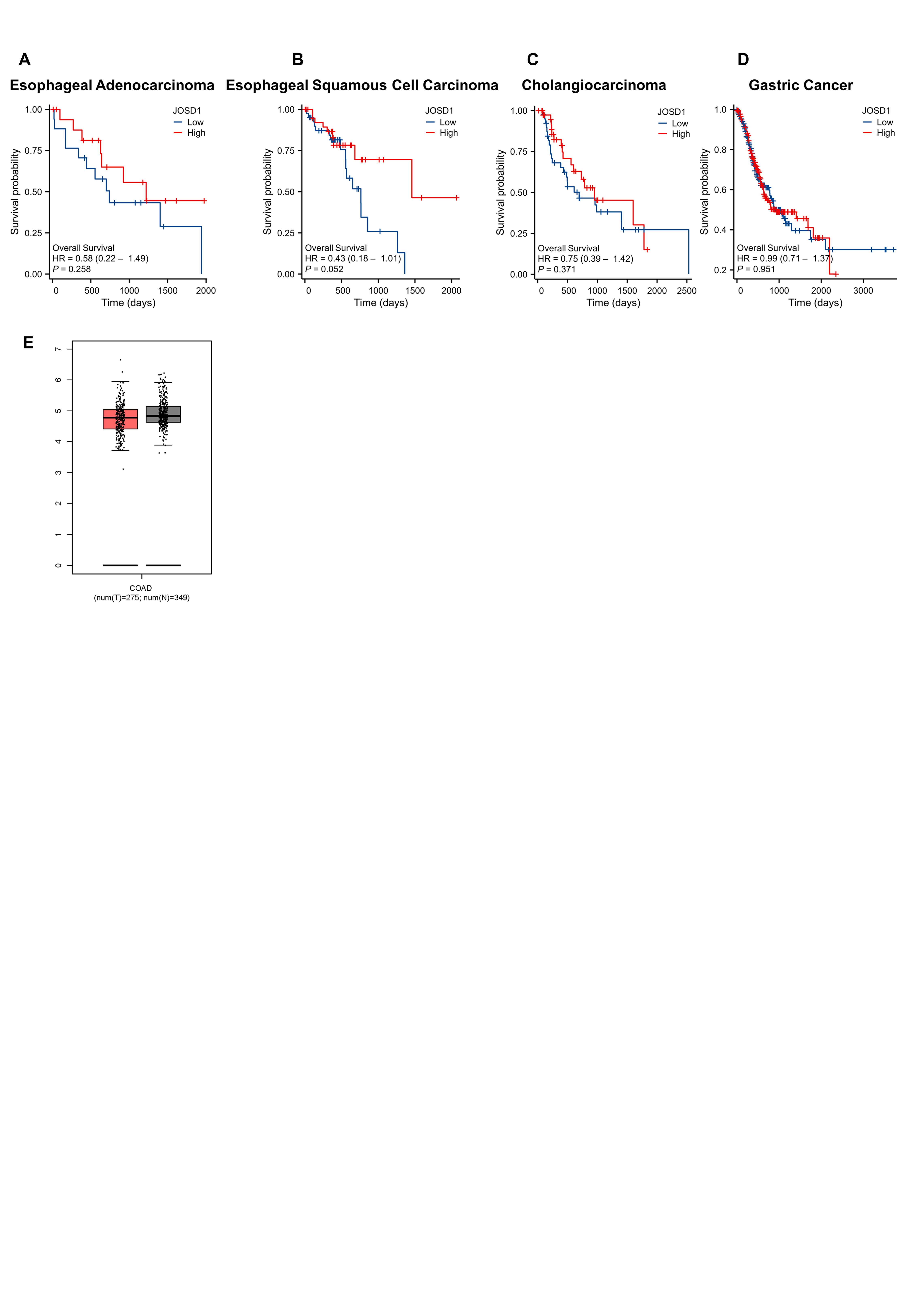

Supplement: Supplementary file 3 — Supplementary Figure 1 [file 41420_2024_2136_MOESM3_ESM.tif]

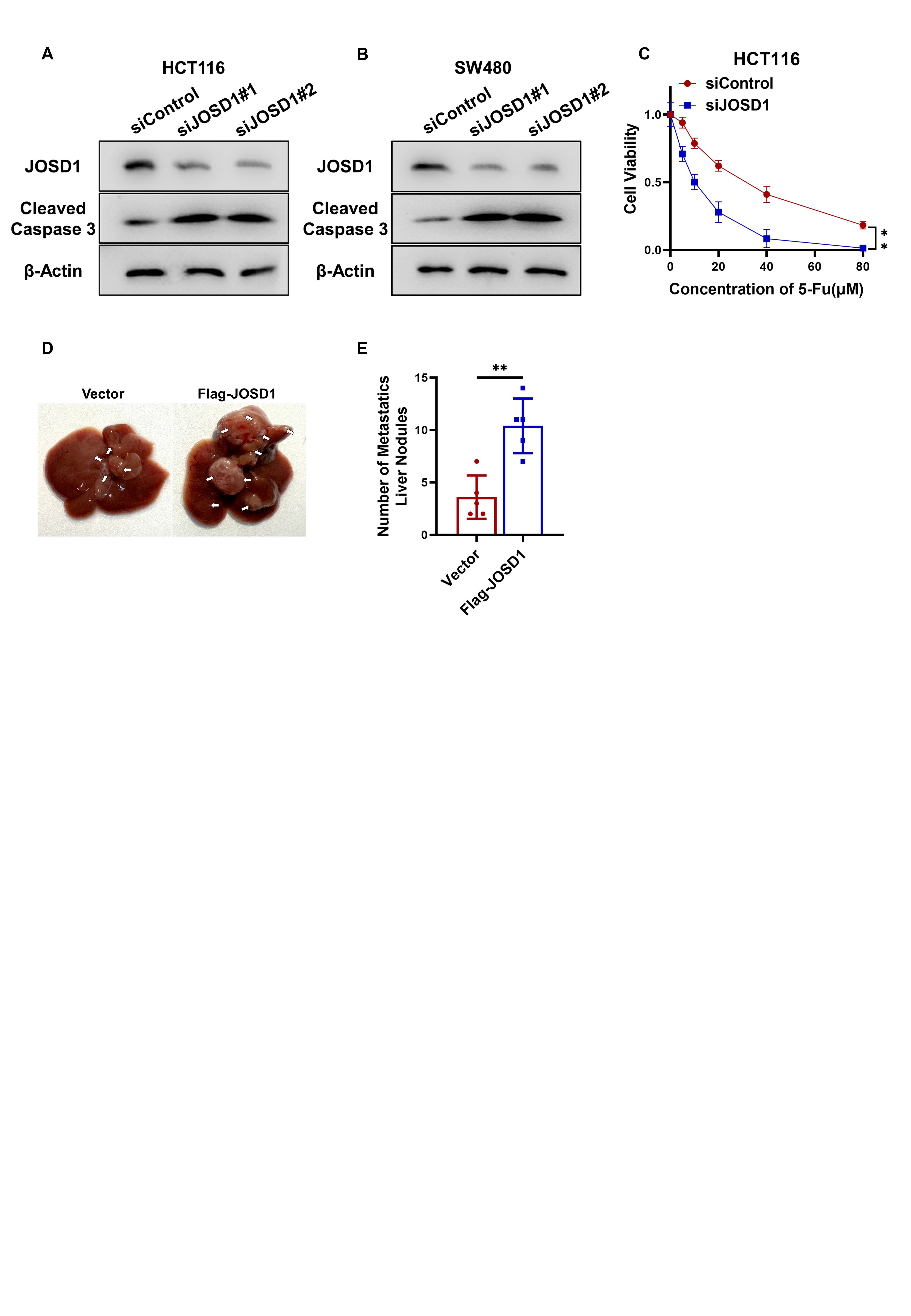

Supplement: Supplementary file 4 — Supplementary Figure 2 [file 41420_2024_2136_MOESM4_ESM.tif]

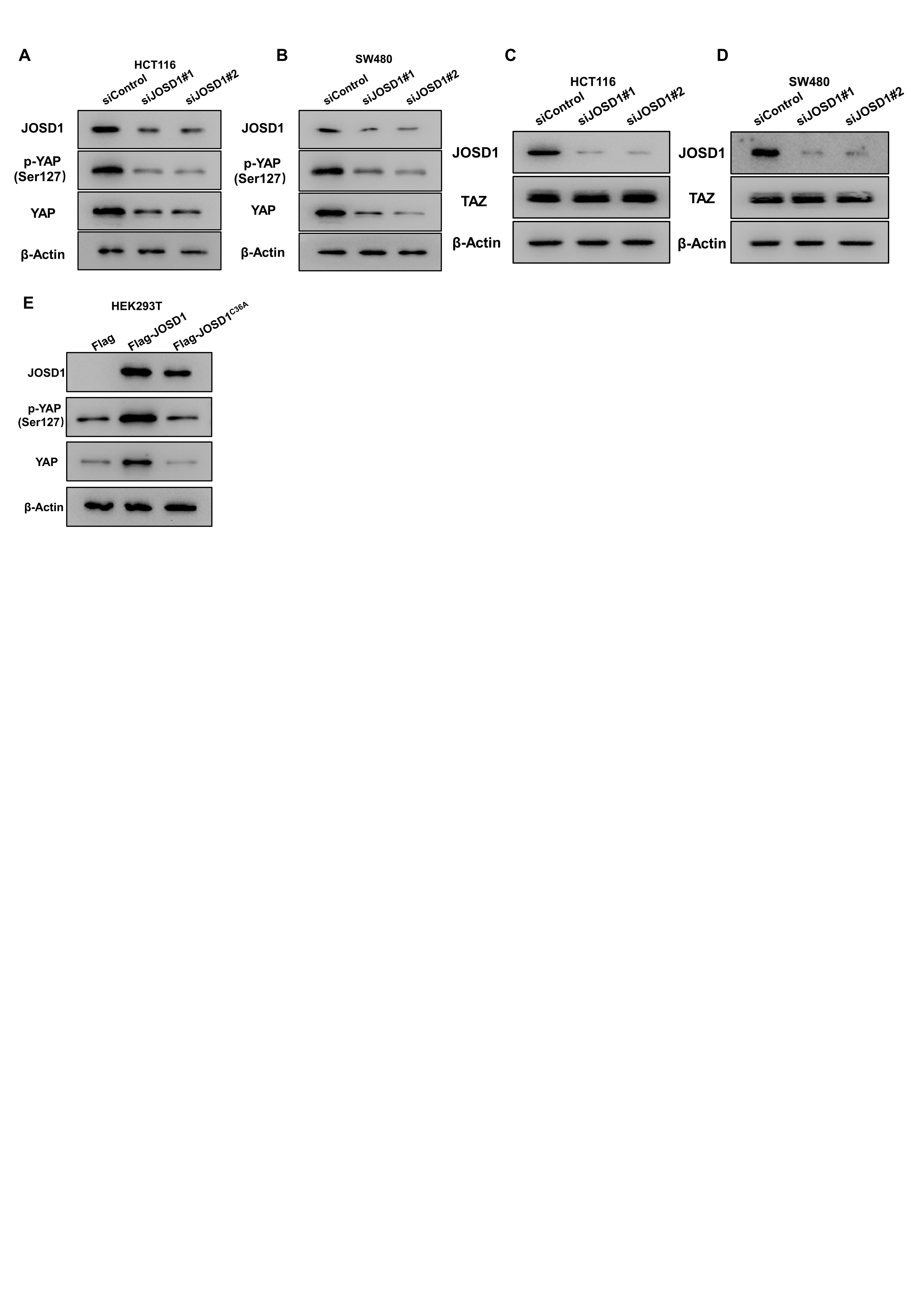

Supplement: Supplementary file 5 — Supplementary Figure 3 [file 41420_2024_2136_MOESM5_ESM.tif]

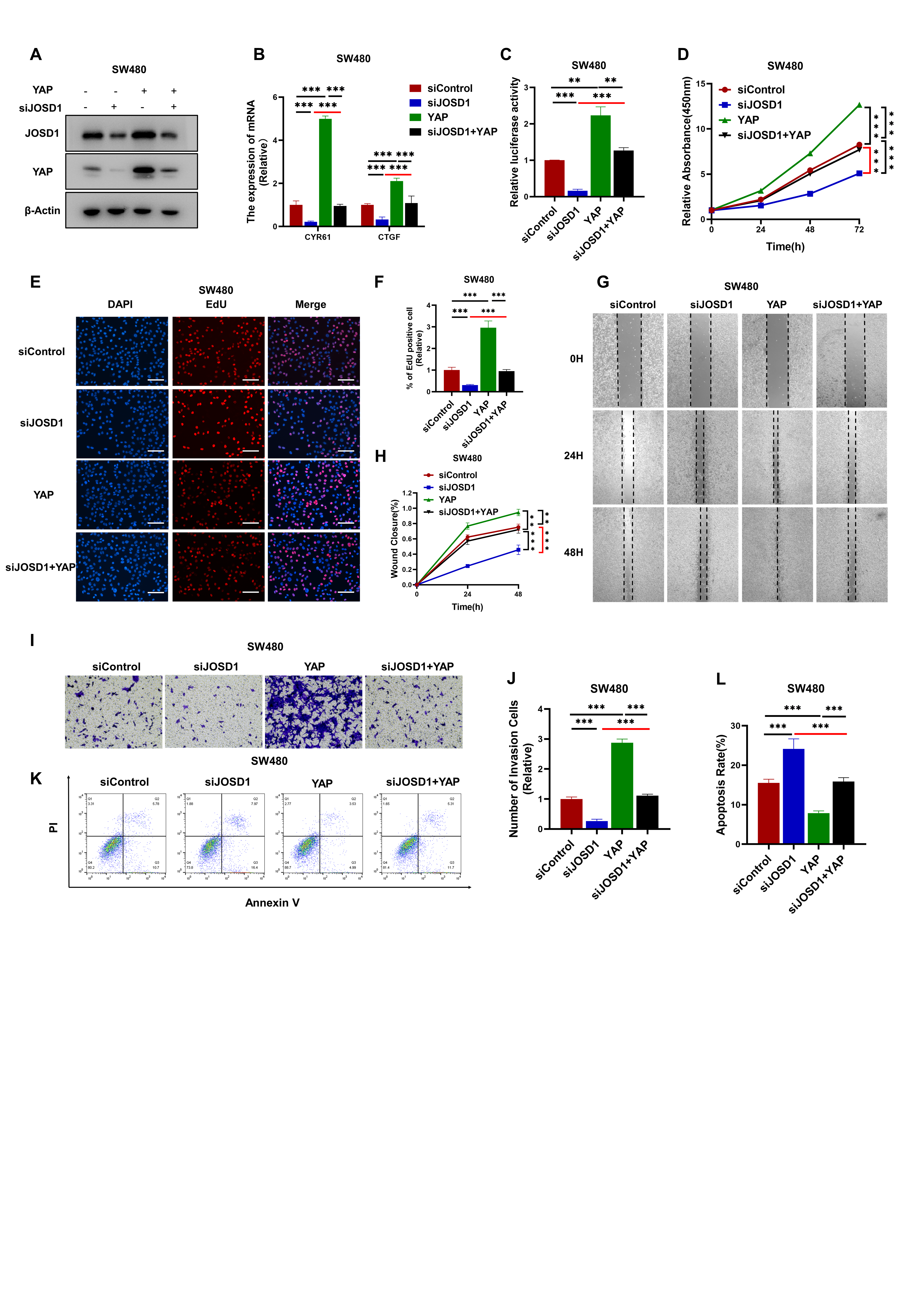

Supplement: Supplementary file 6 — Supplementary Figure 4 [file 41420_2024_2136_MOESM6_ESM.tif]

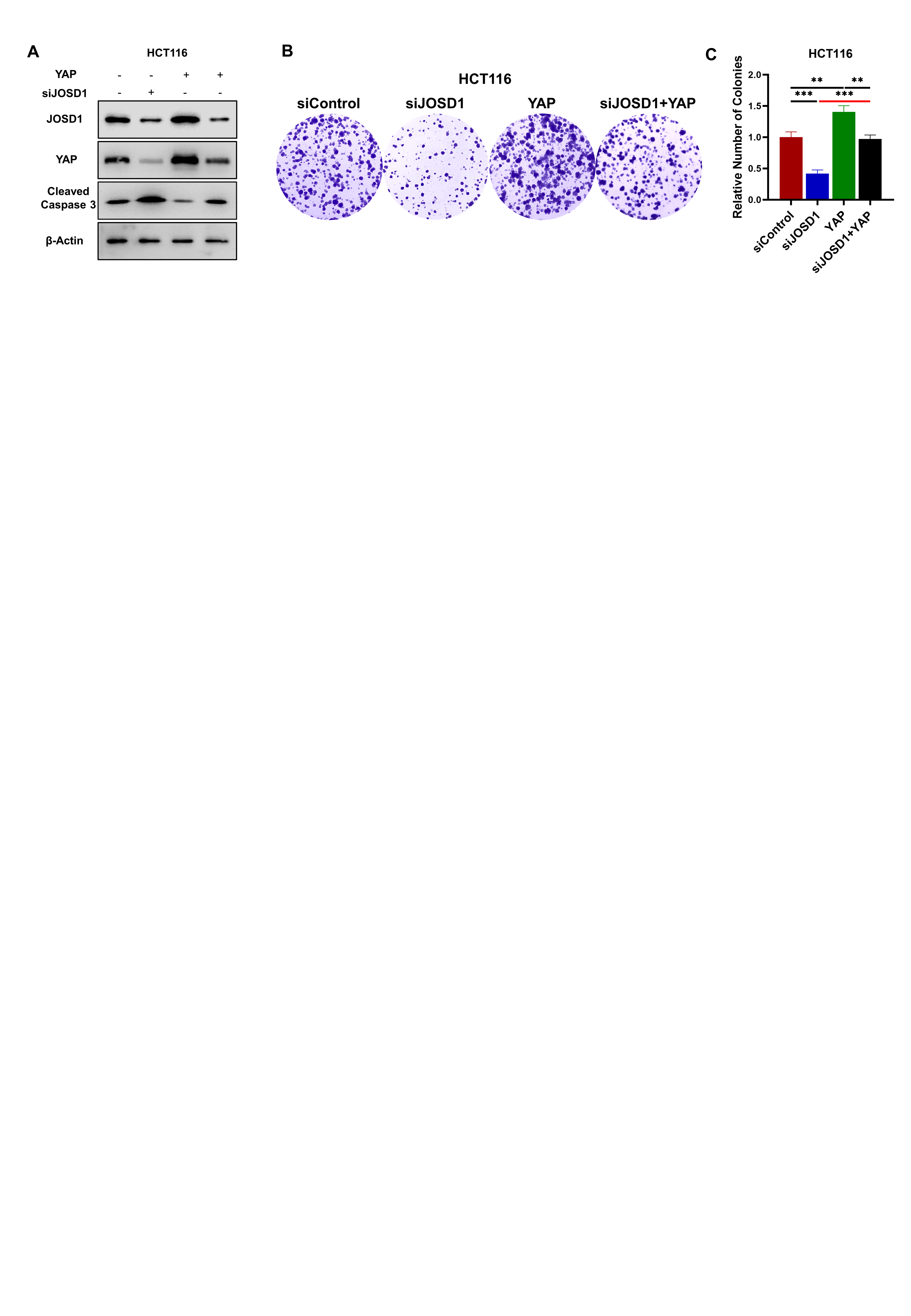

Supplement: Supplementary file 7 — Supplementary Figure 5 [file 41420_2024_2136_MOESM7_ESM.tif]

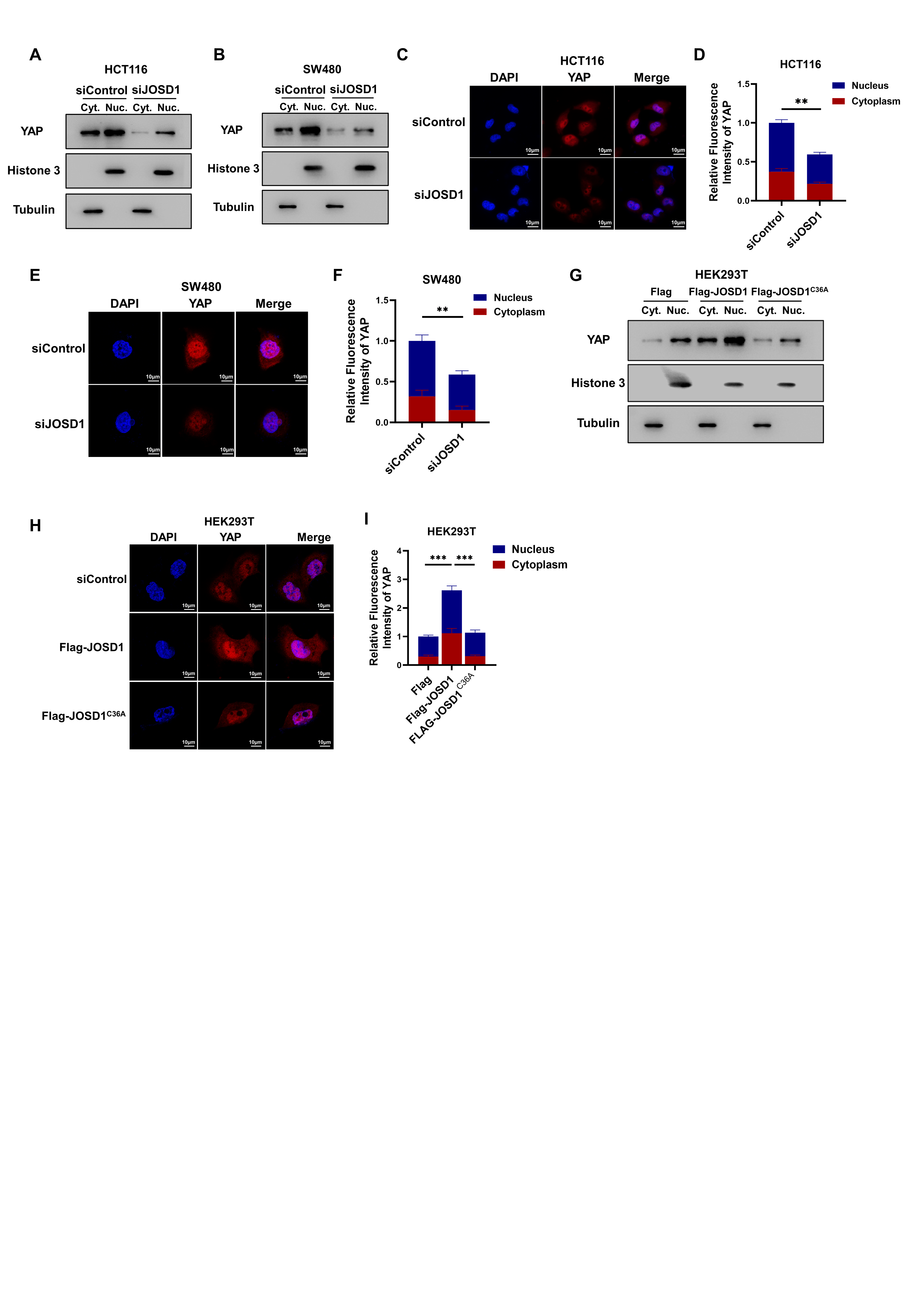

Supplement: Supplementary file 8 — Supplementary Figure 6 [file 41420_2024_2136_MOESM8_ESM.tif]
