# Supplementary material for: Regulation of Hippo/YAP axis in colon cancer progression by the deubiquitinase JOSD1
Source: Cell Death Discov. 2024 Aug 14;10:365. doi: 10.1038/s41420-024-02136-7 (PMC11325045; doi:10.1038/s41420-024-02136-7)
Supplement: Supplementary file 9 — origin data [file 41420_2024_2136_MOESM9_ESM.pdf]

Figure 1A

USP29	0.943
UCHL1	0.866
USP17L2	0.776
USP18	0.736
UBTD1	0.646
USP13	0.575
USP44	0.567
USP51	0.536
USP32	0.46345
JOSD1	0.367

Figure 2A

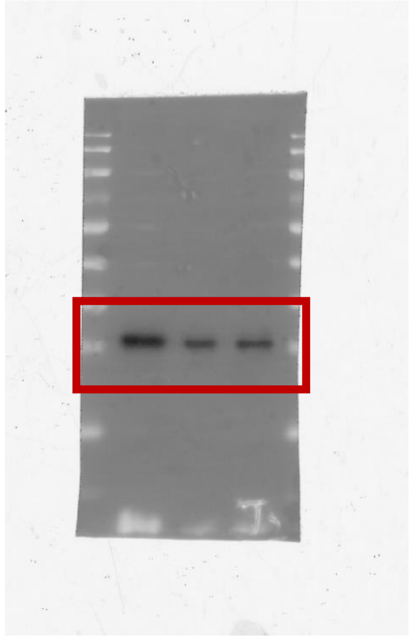

JOSD1

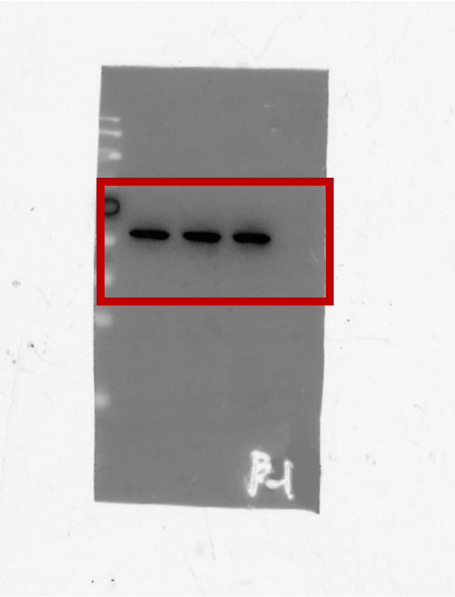

β-actin

Figure 2B

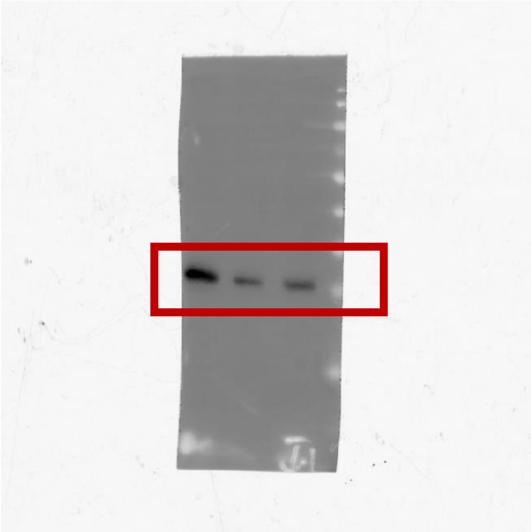

JOSD1

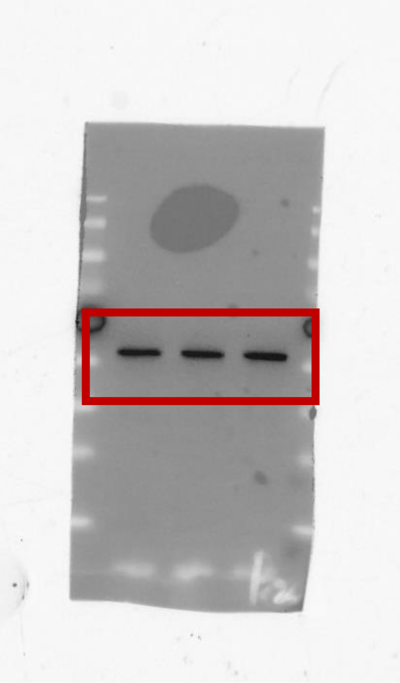

β-actin

Figure 2C

siControl	siJOSD1#1	siJOSD1#2
0.930825	0.550982	0.221481
0.954011	0.449079	0.170904
1.115165	0.366023	0.187439

Figure 2D

siControl	siJOSD1#1	siJOSD1#2
1.11248	0.112579	0.182769
0.99423	0.09233	0.132183
0.89329	0.056911	0.161566

Figure 2E

Time (h)	siControl			siJOSD1#1			siJOSD1#2		
0	1.016686	0.97012	1.013194	1.035962	1.023702	0.9481	1.010373	0.977178	1.012449
24	2.456345	2.467986	2.516492	1.295464	1.326114	1.236208	1.53527	1.595436	1.53112
48	4.136593	4.142414	4.167637	1.873723	1.892113	1.765427	2.072614	2.458506	2.377593
72	6.53279	6.395033	6.505627	4.092767	4.062117	4.017164	4.209544	4.074689	4.180498

Figure 2F

Time(h)	siControl			siJOSD1#1			siJOSD1#2		
0	1.027998	0.98412	0.987882	0.978134	1.030847	0.991019	0.990484	0.992647	1.016869
24	2.513581	2.55328	2.584622	1.14018	1.233893	1.105037	1.541955	1.550606	1.589533
48	4.534058	4.56122	4.607188	2.284264	2.301835	2.313549	2.737889	2.921713	2.884948
72	7.124948	7.139574	7.246135	3.592347	3.664584	3.799297	4.273356	4.437716	4.262543

Figure 2H

siControl	siJOSD1#1	siJOSD1#2
0.928688	0.376411	0.248033
1.106504	0.406185	0.173655
0.964808	0.309264	0.143943

Figure 2J

siControl	siJOSD1#1	siJOSD1#2
0.97745245	0.3152376458	0.28346826
0.96246934	0.2724767459	0.196312759
1.06007821	0.2462809113	0.153363

Figure 2L

siControl	siJOSD1#1	siJOSD1#2
1.03436543	0.2756377075	0.1678825
0.861477	0.29352334787	0.19664734
1.10415757	0.267534287987	0.1647024

Figure 2N

siControl	siJOSD1#1	siJOSD1#2
0.95823746	0.25326	0.24731469429
1.11383574	0.231819	0.2034778
0.9279268	0.241505	0.1741274519

Figure 2P

siControl	siJOSD1#1	siJOSD1#2
3.84	9.84	10.97
3.59	11.03	10.76
4	11.14	10.18

Figure 2R

siControl	siJOSD1#1	siJOSD1#2
9.69	15.19	14.53
9.96	16.63	15.01
9.73	16.48	13.96

Figure 2T

Time(h)	siControl			siJOSD1#1			siJOSD1#2		
0	0	0	0	0	0	0	0	0	0
24	0.77	0.78	0.68	0.45	0.49	0.43	0.42	0.43	0.46
48	0.97	0.94	0.98	0.75	0.7	0.78	0.67	0.69	0.58

Figure 2V

Time(h)	siControl			siJOSD1#1			siJOSD1#2		
0	0	0	0	0	0	0	0	0	0
24	0.70	0.79	0.64	0.62	0.57	0.52	0.32	0.37	0.42
48	0.94	1	0.97	0.79	0.75	0.82	0.59	0.53	0.49

Figure 2X

Time (Day)	shControl					shJOSD1				
0	0	0	0	0	0	0	0	0	0	0
7	67	86.7	77	28	15	16.19	19.28	25	24	27.5
14	195	143	145	47.2	49	27.51	29.14	32.4	32.98	31.72
21	278	384	464.3	204	285	104.02	88.11	94.29	64.23	56.05
28	734.6	977	484	269	474.6	207.5	114.6	103	75.4	69
35	850	1152	500	416	600	294	171.5	196	87.5	75

Figure 2Y

shControl	shJOSD1
0.949598	0.328449
1.286984	0.191595
0.558587	0.218966
0.464744	0.097753
0.670304	0.083788

Figure 2Z

shControl	shJOSD1
189	14
80	21
86	27
163	31
107	11

Figure 3A

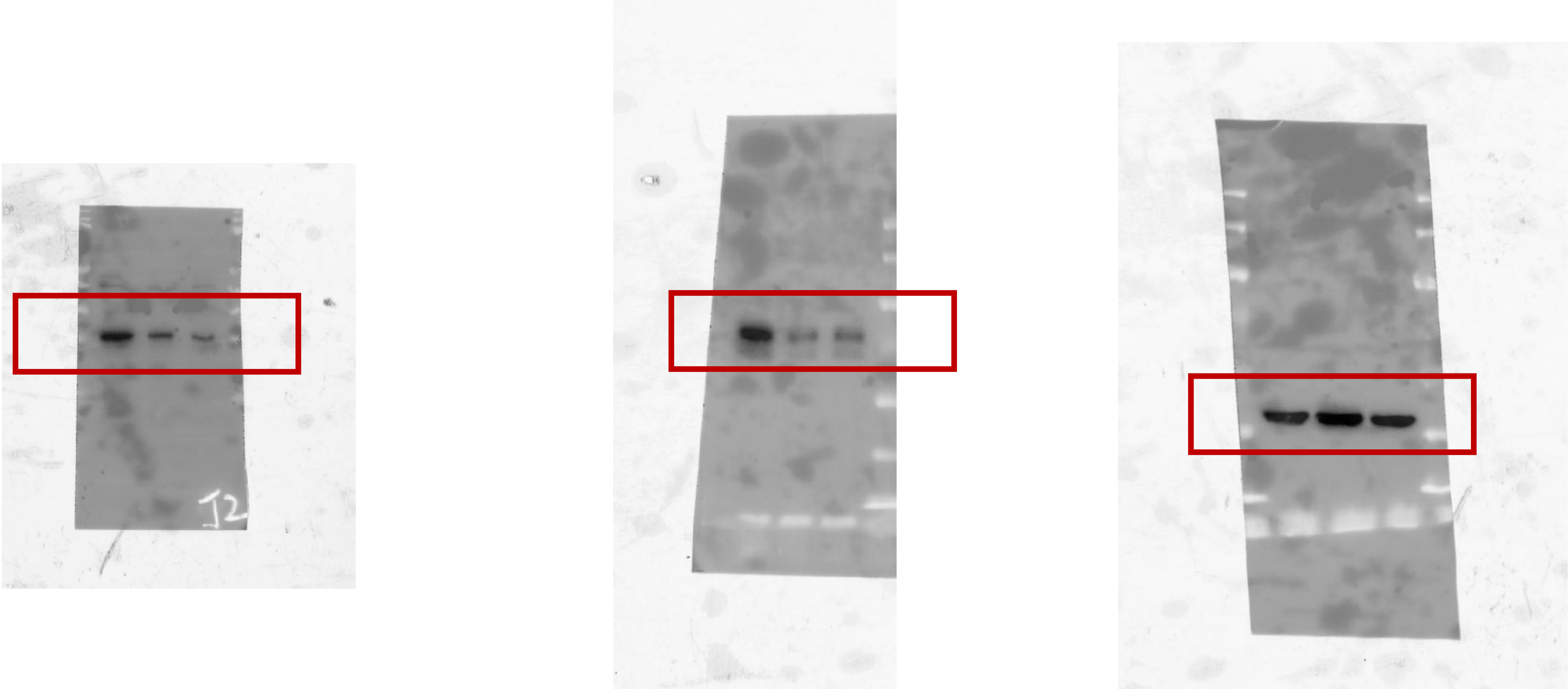

JOSD1 | YAP | β-actin

Figure 3B

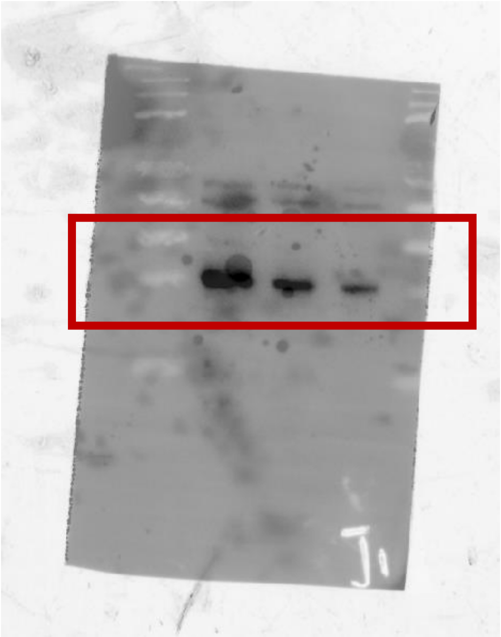

JOSD1

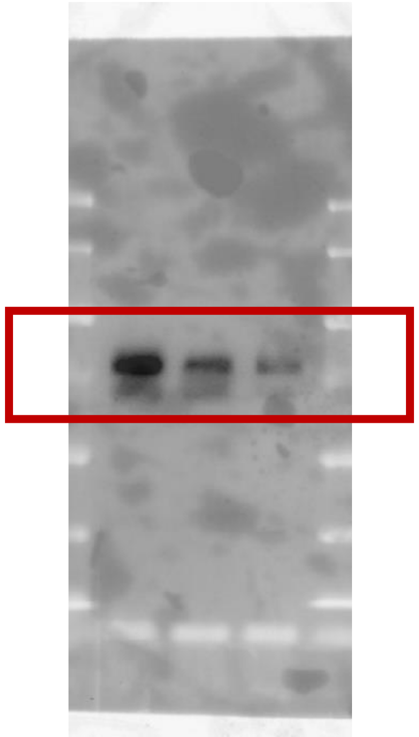

YAP

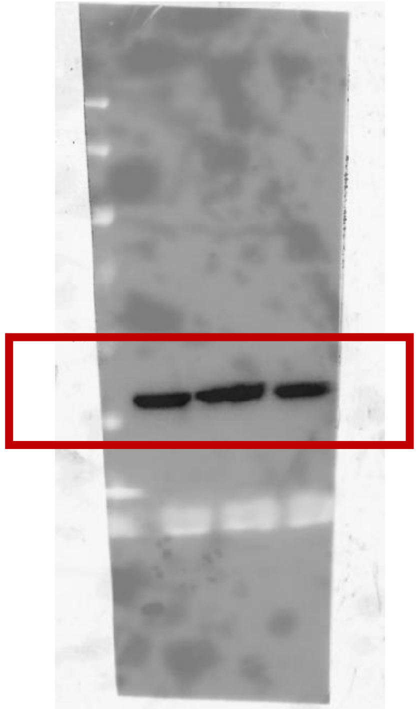

β-actin

Figure 3C

siControl	siJOSD1#1	siJOSD1#2
1.14615	0.952847	0.731617
0.961876	0.96934	0.728385
0.891974	0.707341	0.915305

Figure 3D

siControl	siJOSD1#1	siJOSD1#2
0.938635	0.843534	0.814421
0.946641	0.873132	1.046778
1.114723	1.005973	1.05593

Figure 3E

	siControl			siJOSD1#1			siJOSD1#2		
CYR61	1.21701	0.892092	0.890898	0.066526	0.069858	0.067519	0.205334	0.233555	0.170395
CTGF	1.097476	0.894214	1.008309	0.319648	0.300488	0.352764	0.188685	0.199236	0.180905

Figure 3F

	siControl			siJOSD1#1			siJOSD1#2		
CYR61	0.931889	0.998103	1.070008	0.265576	0.222033	0.171719	0.202352	0.23603	0.297536
CTGF	1.058692	0.90462	1.036688	0.37399	0.417414	0.403903	0.274708	0.280455	0.319313

Figure 3G

siControl	siJOSD1#1	siJOSD1#2
1.094327	0.34631	0.228198
1.018019	0.373704	0.159769
0.887654	0.284533	0.132432

Figure 3H

siControl	siJOSD1#1	siJOSD1#2
1.170716	0.286072	0.187722
0.886151	0.263296	0.177611
0.947134	0.274684	0.140242

Figure 3K

	Flag			Flag-JOSD1			Flag-JOSD1^{C36A}		
CYR61	1.073635	0.925421	1.000944	2.257088	2.383053	2.309433	0.722671	0.812349	0.788852
CTGF	0.952145	1.034061	1.013794	1.810792	2.433573	2.245578	0.847187	0.964107	0.903758

Figure 3L

Flag	Flag-JOSD1	Flag-JOSD1^{C36A}
1.027134	3.096746	0.983316
1.102609	3.174195	0.896298
0.870257	3.19187	0.938527

Figure 3J

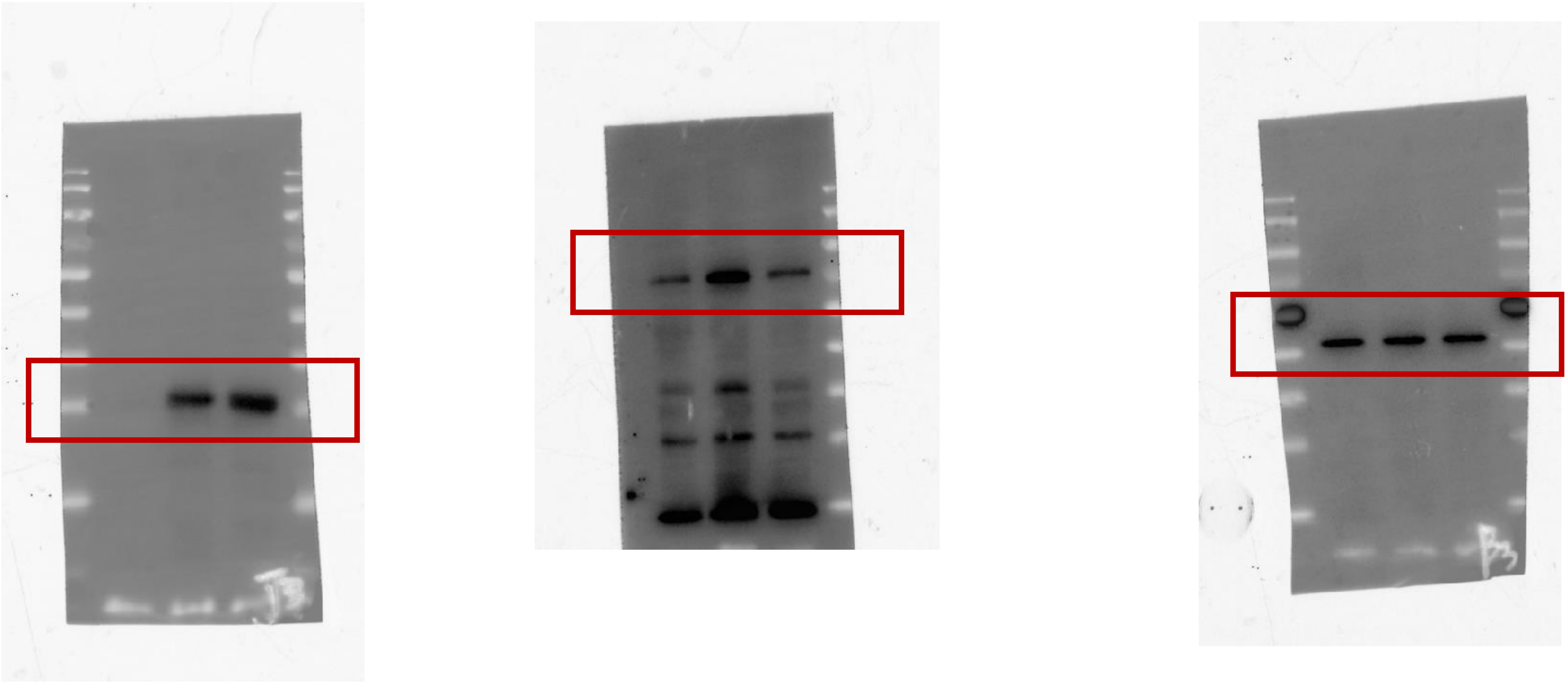

FLAG YAP β-actin

Figure 4A

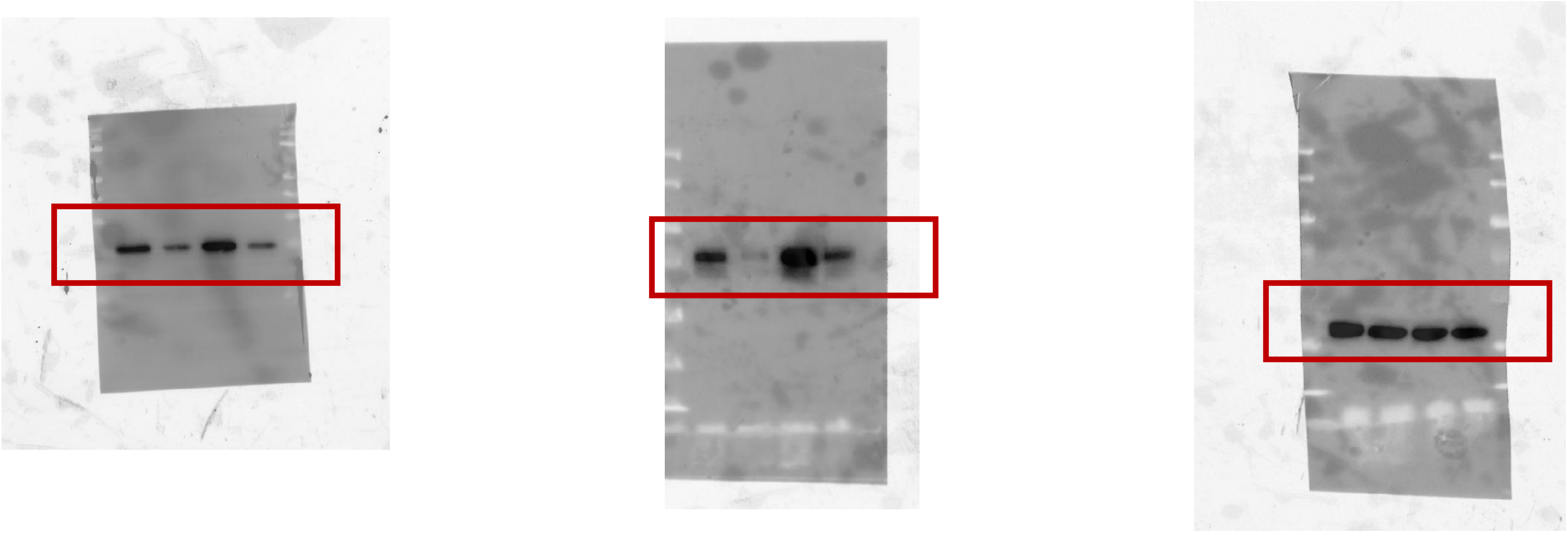

JOSD1 YAP β-actin

Figure 4B

	siControl			siJOSD1#1			YAP			siJOSD1+YAP		
CYR61	1.130645	1.077407	0.791948	0.255725	0.267193	0.299227	3.397339	4.222633	3.686984	1.245708	1.253512	0.999496
CTGF	0.934625	0.984462	1.080913	0.359278	0.336058	0.258366	3.043693	3.383892	4.359205	1.357317	1.169198	1.335052

Figure 4C

siControl	siJOSD1	YAP	siJOAD1+YAP
0.929907	0.382758	2.715274	0.960547
1.104649	0.402498	2.730592	0.83267
0.965444	0.365535	2.680646	1.202045

Figure 4D

Time(h)	siControl			siJOSD1			YAP			siJOSD1+YAP		
0	1.027246	1.045655	0.927099	0.892193	0.981413	1.003717	0.971119	0.978339	1.036101	1.087581	0.897344	0.933238
24	3.177467	3.177467	3.151694	2.501795	2.096195	2.157215	5.620939	5.862816	5.837545	3.037175	3	2.959108
48	6.215613	6.219331	6.159851	3.132807	3.139986	3.251256	9.375451	9.364621	9.534296	5.854197	5.83947	5.828424
72	9.02974	9.36803	9.379182	4.835607	4.853553	4.47308	14.6787	15.01805	14.63538	8.468336	8.173785	8.379971

Figure 4F

siControl	siJOSD1	YAP	siJOSD1+YAP
0.828688	0.368236487	2.7736814	0.959877476
1.206504	0.43746782	2.778487	0.77982746
0.964808	0.384692	2.6823794	1.17874

Figure 4H

Time(h)	siControl			siJOSD1			YAP			siJOSD1+YAP		
0	0	0	0	0	0	0	0	0	0	0	0	0
24	0.40458	0.435878	0.381679	0.144444	0.211111	0.2666667	0.854167	0.583333	0.666667	0.429752	0.479339	0.355372
48	0.68885	0.669389	0.678092	0.333333	0.455556	0.3	0.958333	0.961667	0.984167	0.748099	0.727273	0.72479

Figure 4J

siControl	siJOSD1	YAP	siJOSD1+YAP
1.012658	0.253165	3.594937	0.977215
0.924051	0.341772	3.658228	0.983797
1.063291	0.329114	3.468354	1.009241

Figure 4L

siControl	siJOSD1	YAP	siJOSD1+YAP
7.77	13.78	3.74	8.94
7.83	13.07	3.28	8.07
7.03	12.6	3.95	8.46

Figure 4N

Time (Day)	shControl					shJOSD1					YAP					shJOSD1+YAP				
0	0	0	0	0	0	0	0	0	0	0	0	0	0	0	0	0	0	0	0	0
7	18	32	8	5	6	7	5	8	2	4	73	77	93	100	98	20	7	5	4	6
14	53	155	46	39	184	15	18	24	22	16	185	554	170	263	295	43	34	37	40	42
21	275	287	154	135	299	31	34	40	38	32	586	858	690	730	503	74	163	196	274	95
28	548	479	327	276	404	174	137	59	156	138	806	1257	973	838	790	285	429	274	385	394
35	772	769.6	480	384	567	245	269.5	87.5	245	171.5	1296	1605.5	1183	950	1149.5	352	517	392	416	470

Figure 4O

shControl	shJOSD1	YAP	shJOSD1+YAP
0.862179	0.273708	1.44786	0.393245
0.859807	0.301078	1.79362	0.577216
0.536243	0.097753	1.32162	0.437932
0.428995	0.273708	1.06132	0.464744
0.633438	0.191595	1.28419	0.524653

Figure 4Q

shControl	shJOSD1	YAP	shJOSD1+YAP
116	62	284	132
107	46	288	162
129	30	278	110
84	41	204	118
110	21	326	93

Figure 5B

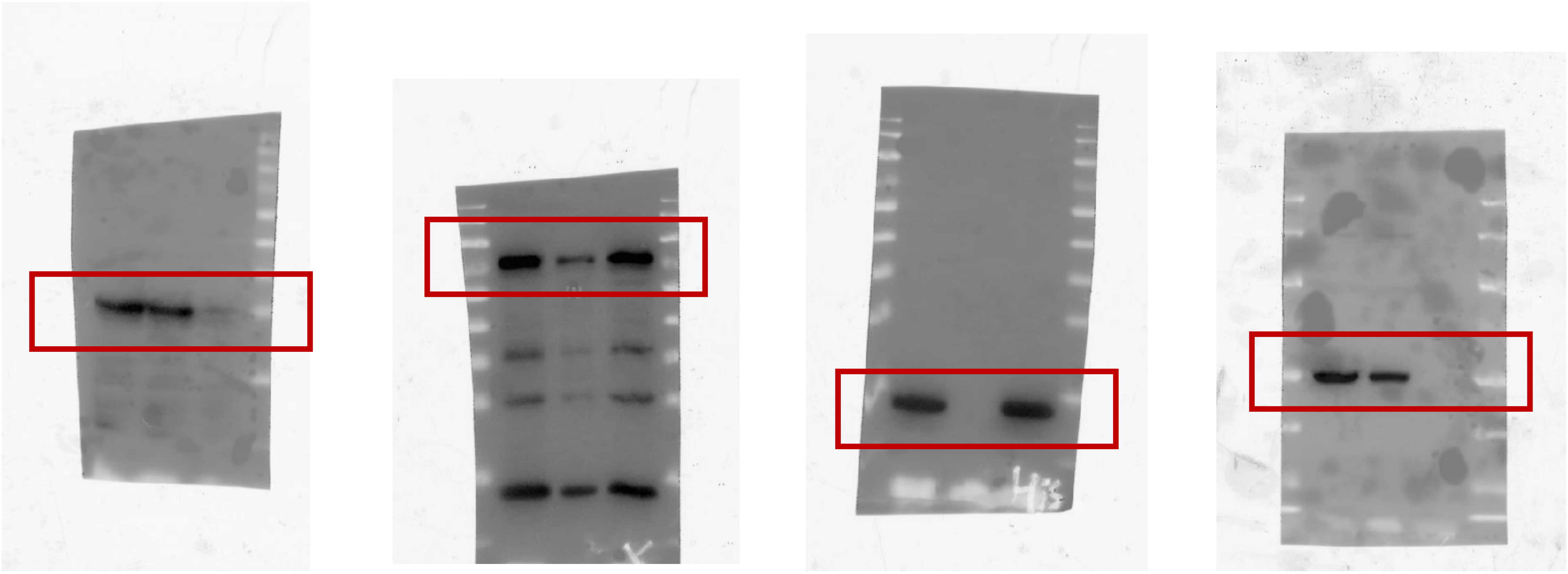

JOSD1 YAP Histone 3 Tubulin

Figure 5C

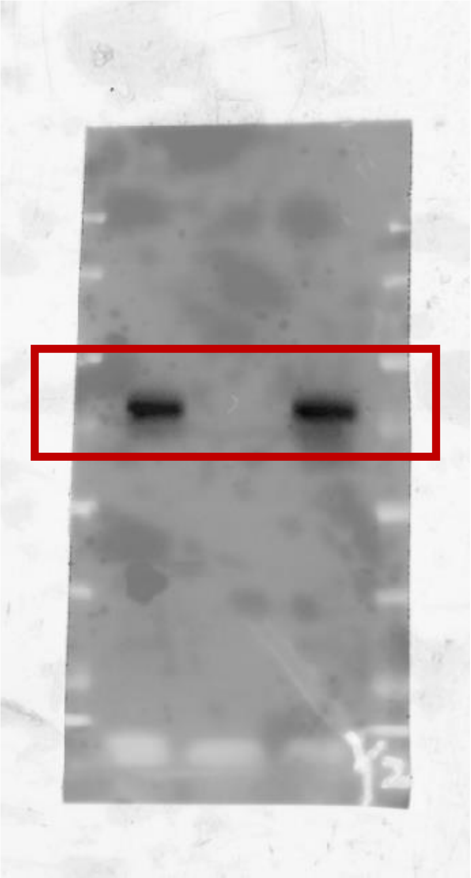

YAP

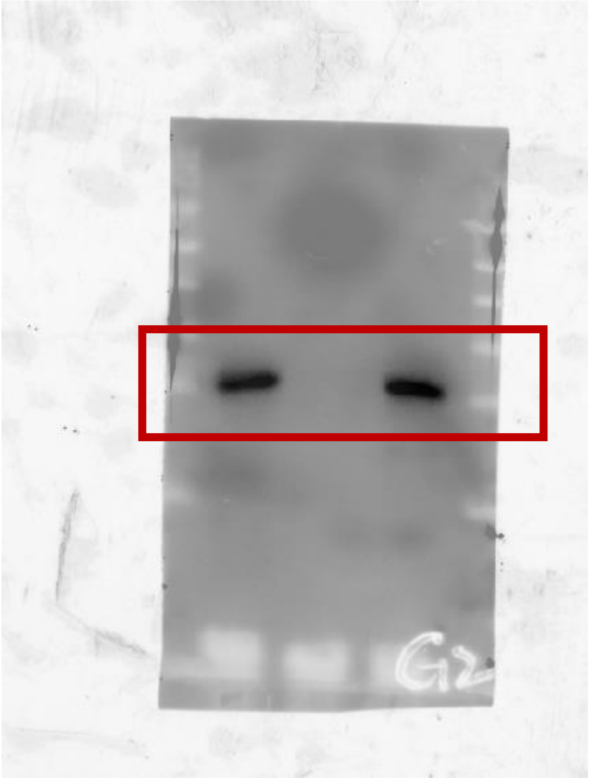

JOSD1

Figure 5D

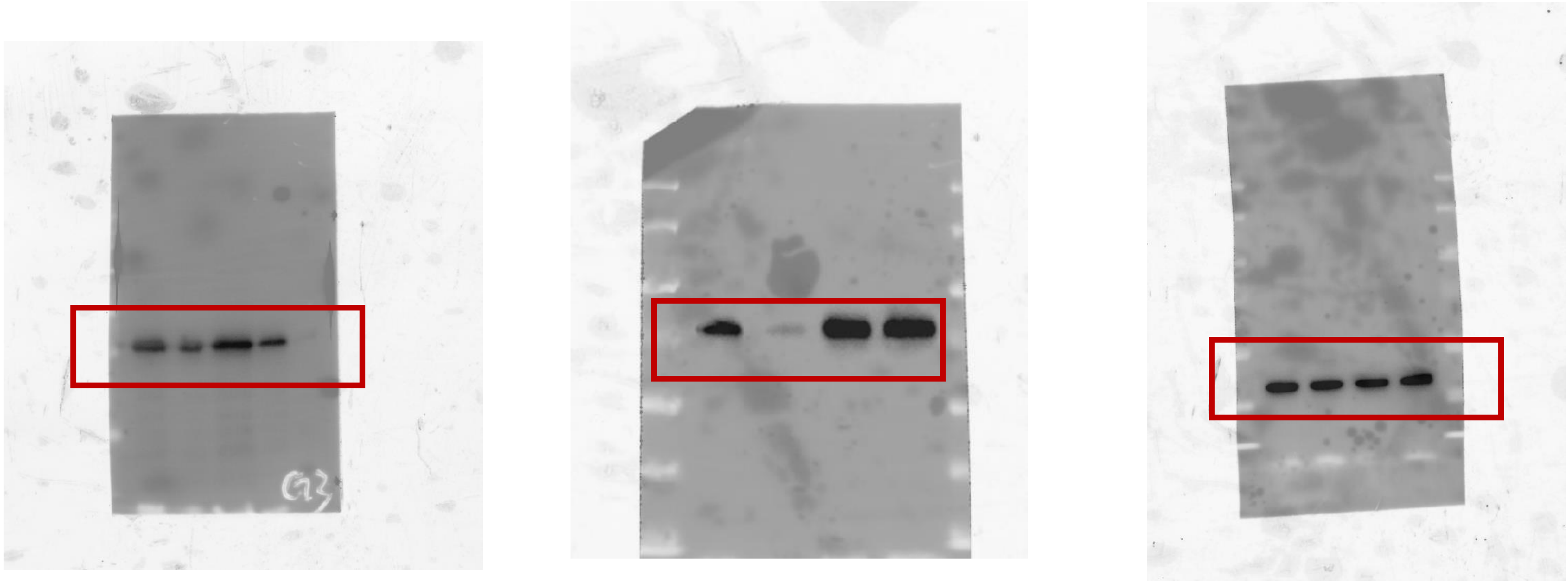

JOSD1 YAP β-actin

Figure 5E

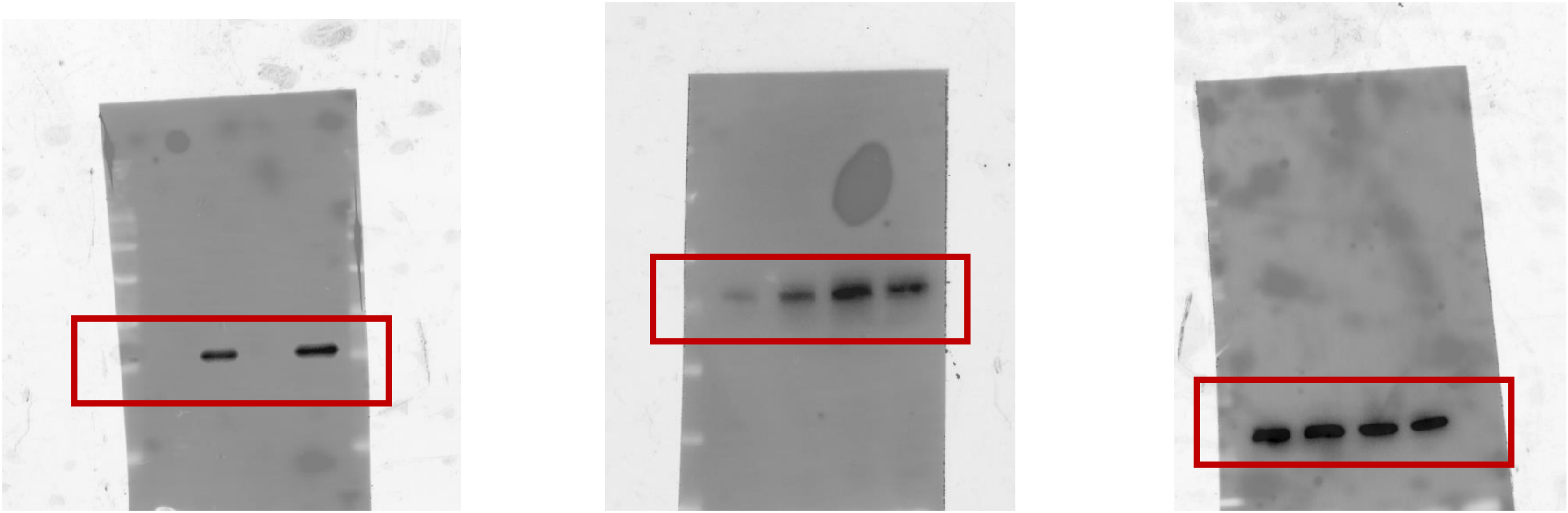

FLAG YAP β-actin

Figure 5F

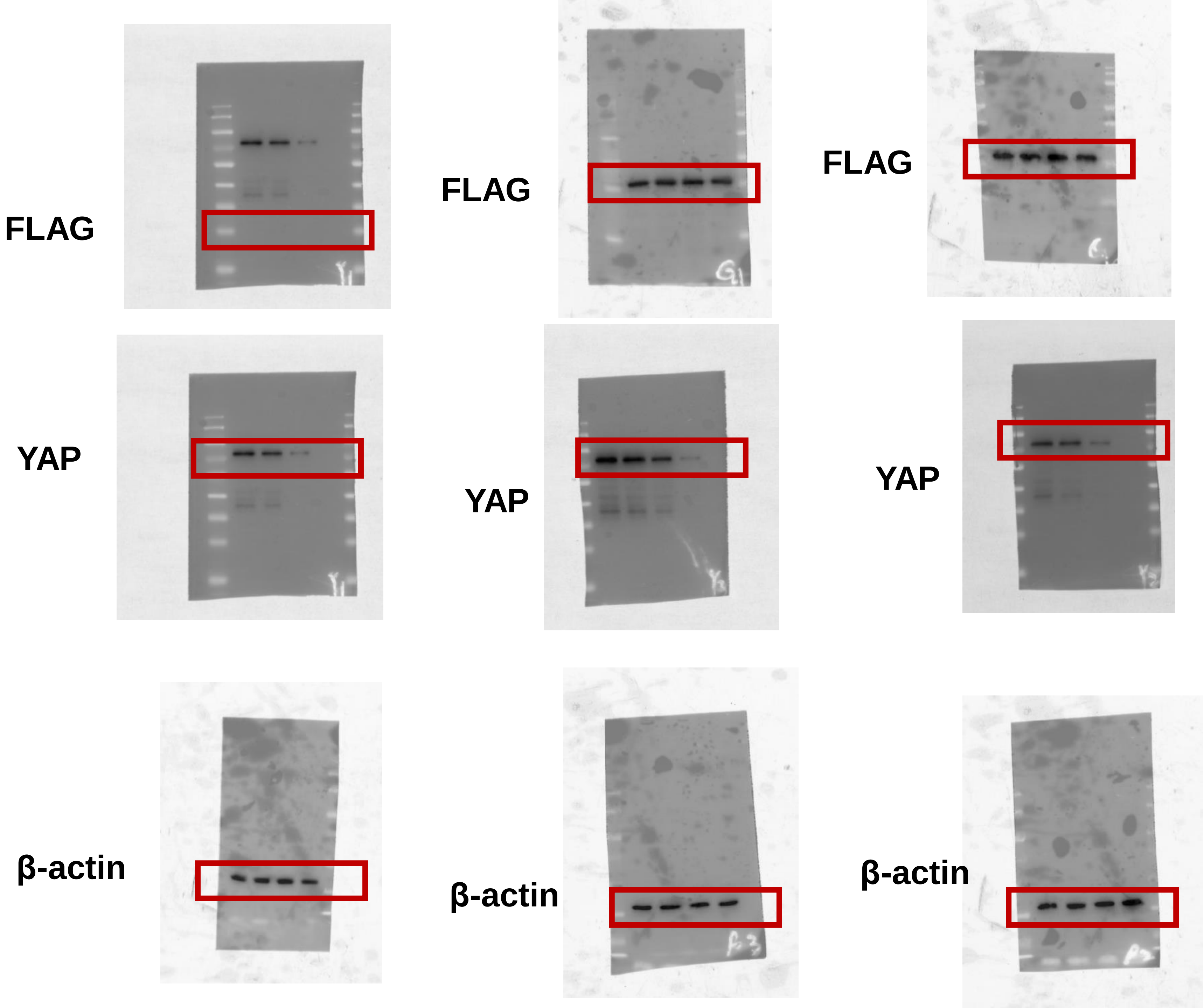

Figure 5G

Time(h)	Flag			Flag-JOSD1			Flag-JOSD1^{C36A}		
0	1.001749	1.025857	0.972394	0.939507318	0.964150507	1.096342176	1.004759	1.012325	0.982917
3	0.866322	0.888336	0.738475	0.992949094	0.97545	0.86856	0.746651	0.6857647	0.85843
6	0.244219	0.1837363	0.224467	0.624339779	0.6566457	0.596436	0.160278	0.135537	0.223643
9	0.014882	0.0186795	0.02147548	0.278729331	0.2486585	0.308578	0.008168	0.0058647	0.00973475

Figure 5I

Time(h)	siControl			siJOSD1		
0	1.002996	0.99937	0.997633	1.337501	0.834436	0.828063
3	0.875504	0.873336	0.872435	0.443412	0.442063	0.439364
6	0.534733	0.534167	0.533023	0.059584	0.059093	0.058073
9	0.137382	0.136877	0.136063	0.003508	0.003046	0.002714

Figure 5H

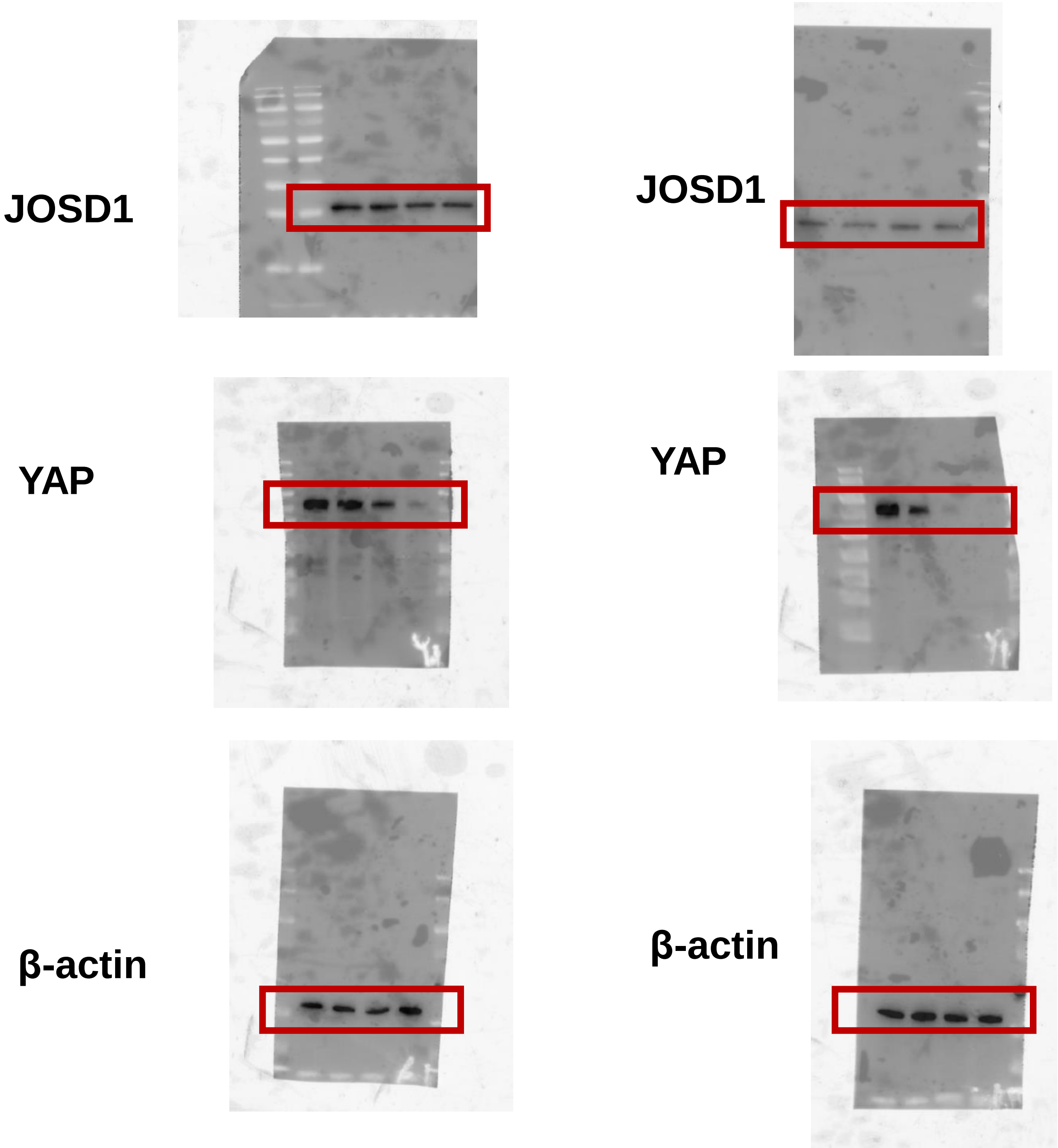

Figure 5L

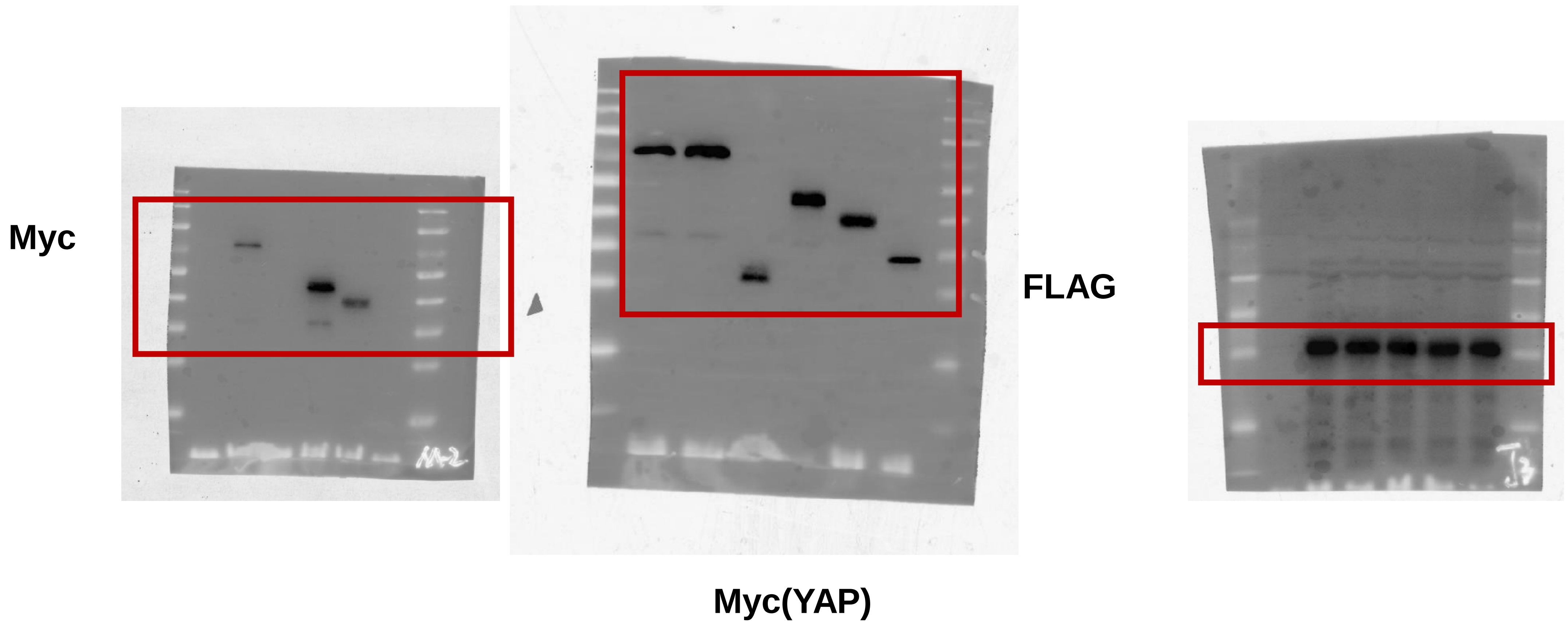

Figure 5M

EGFP

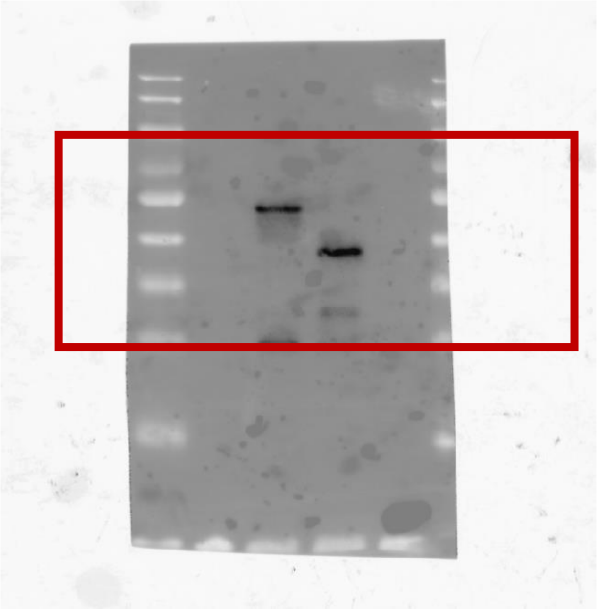

EGFP

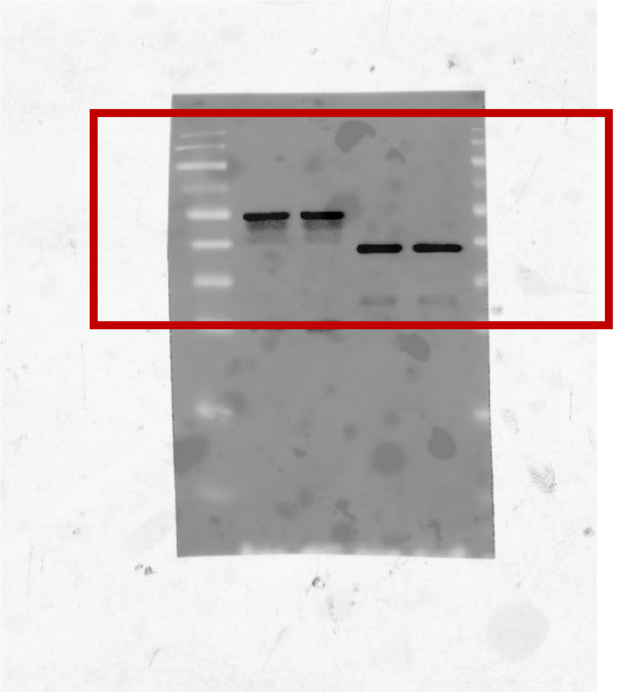

Myc

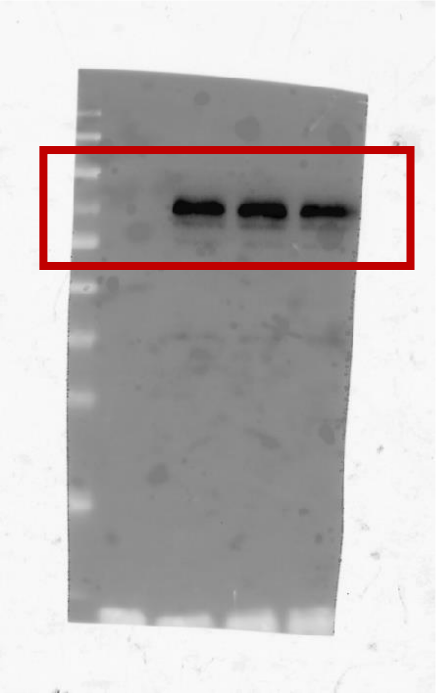

Figure 6A

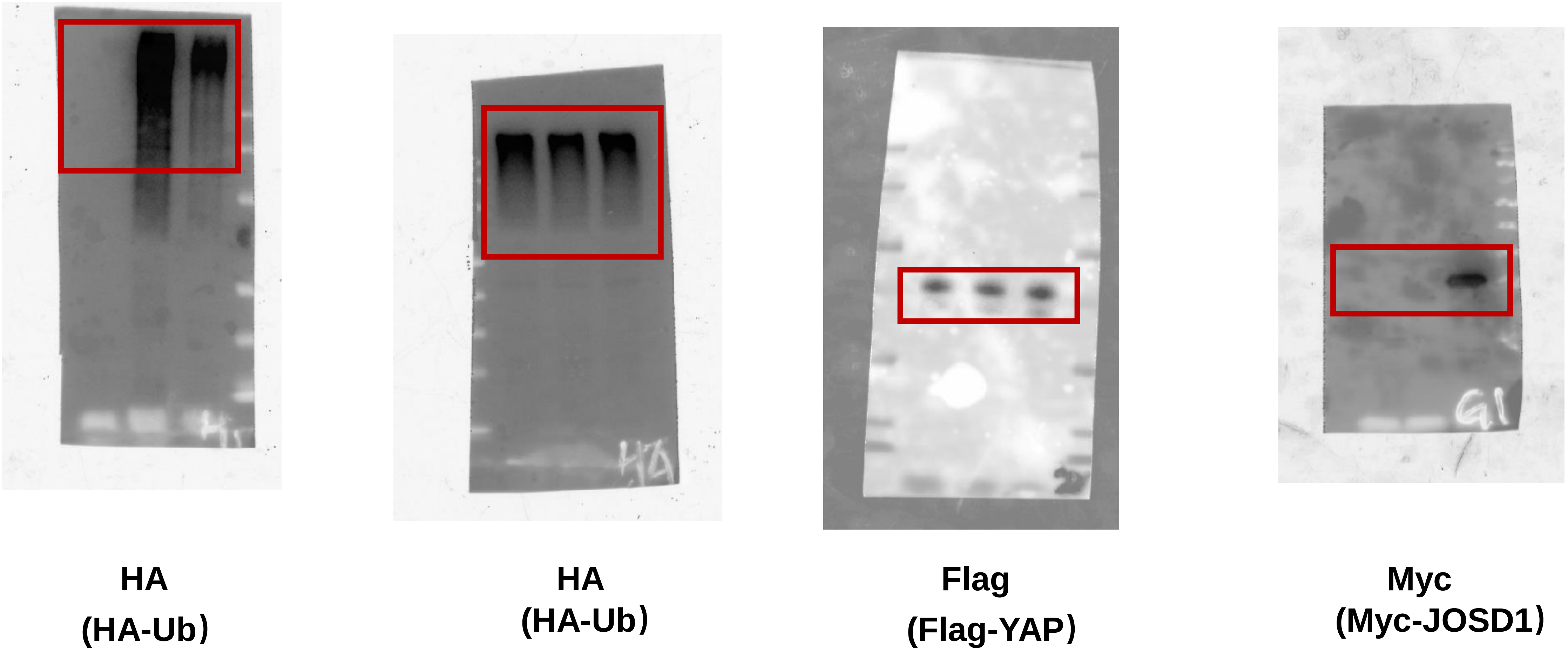

Figure 6B

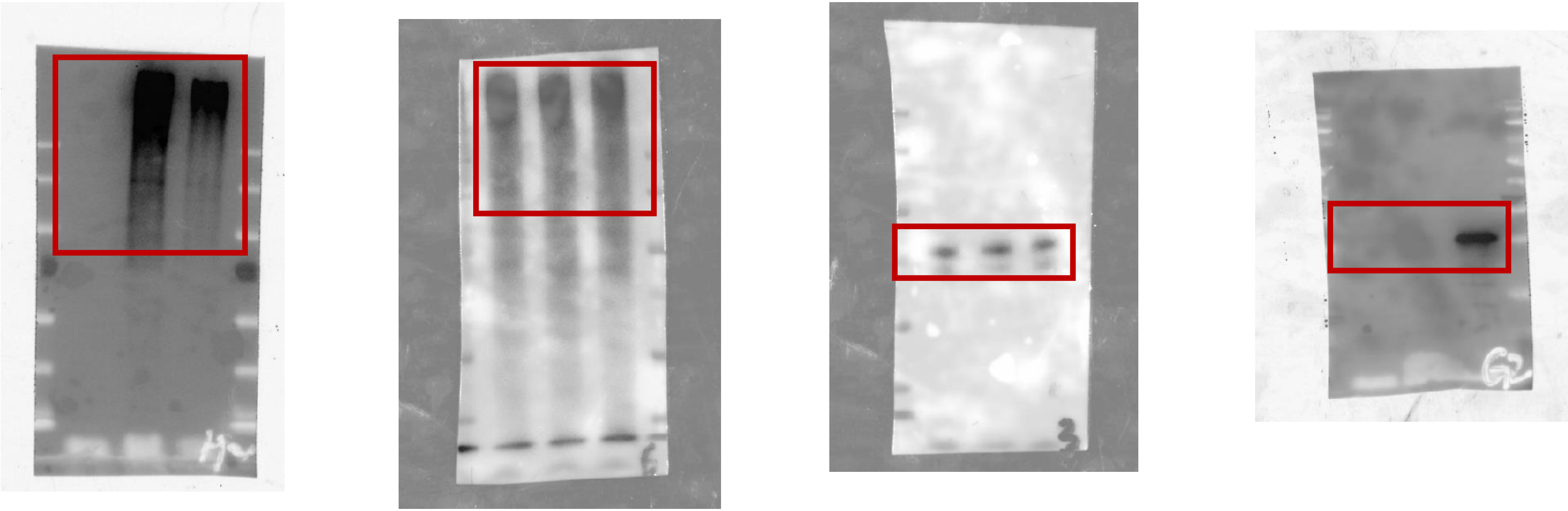

HA (HA-K48-Ub)

HA (HA-K48-Ub)

Flag (Flag-YAP)

Myc (Myc-JOSD1)

Figure 6C

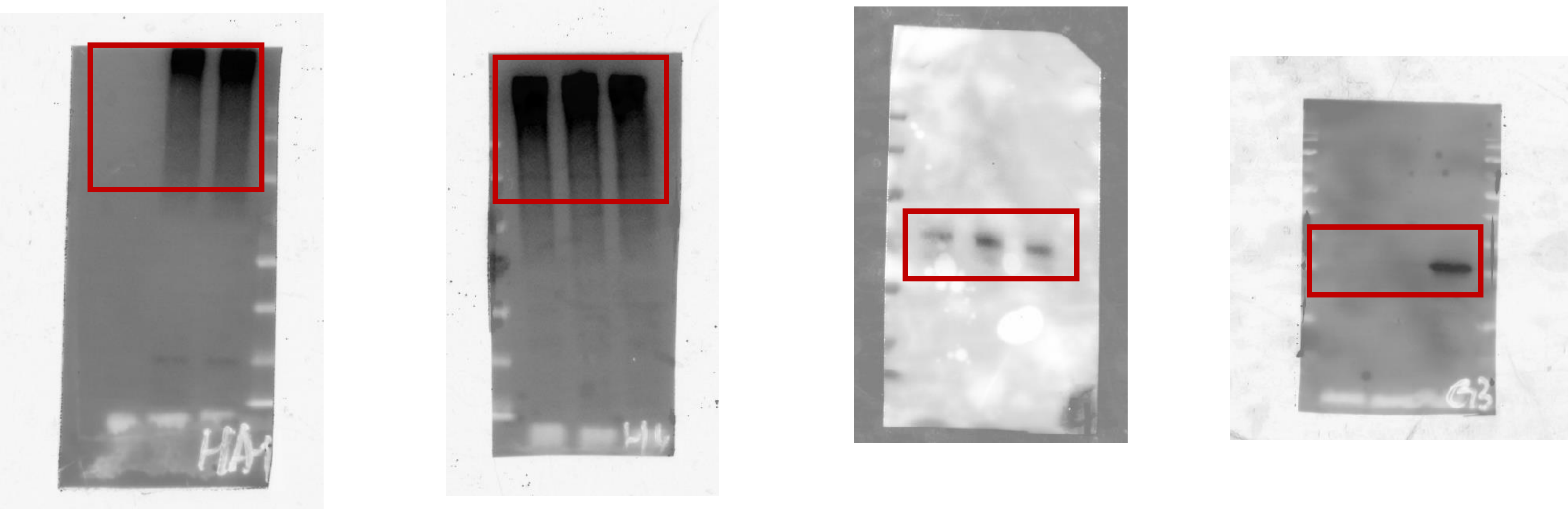

HA (HA-K48R-Ub) | HA (HA-K48R-Ub) | Flag (Flag-YAP) | Myc (Myc-JOSD1)

Figure 6D

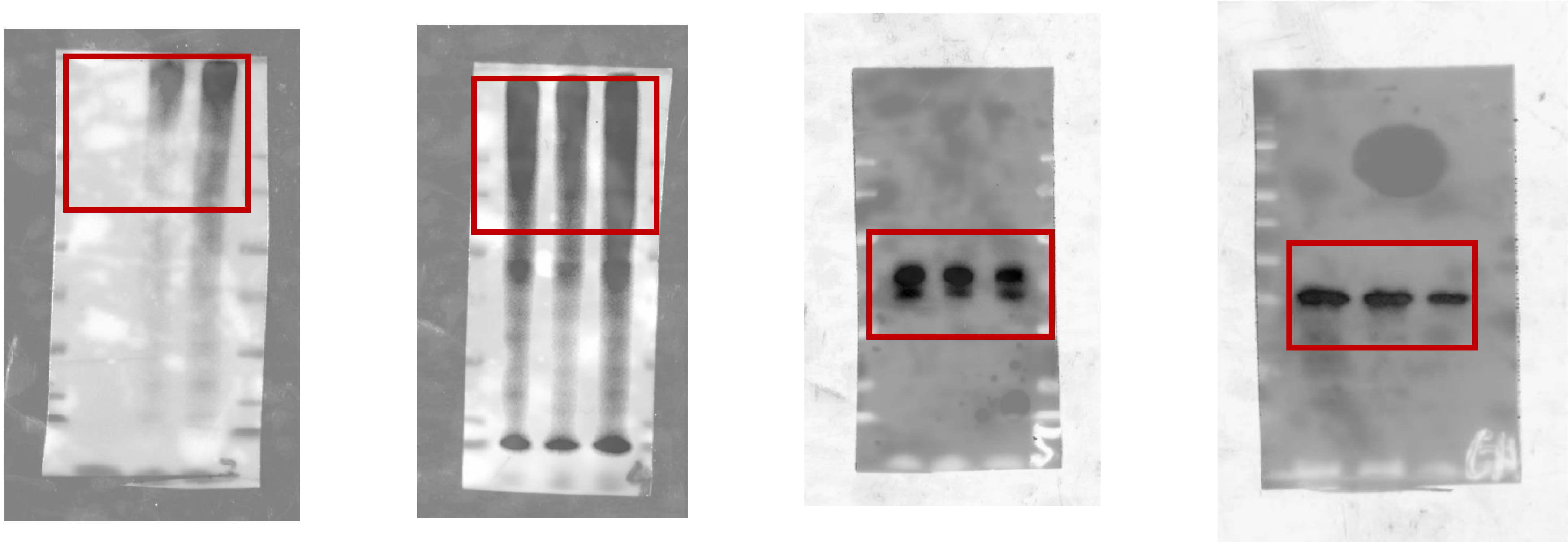

HA (HA-Ub)

HA (HA-Ub)

YAP

JOSD1

Figure 6E

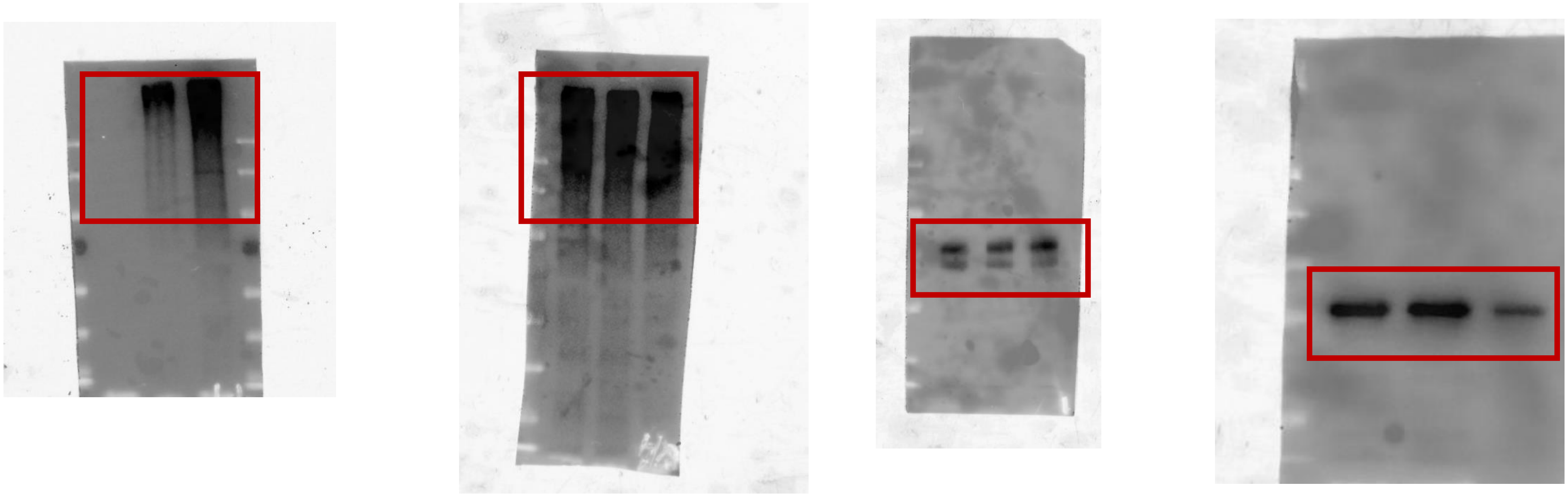

HA (HA-K48-Ub)

HA (HA-K48-Ub)

YAP

JOSD1

Figure 6F

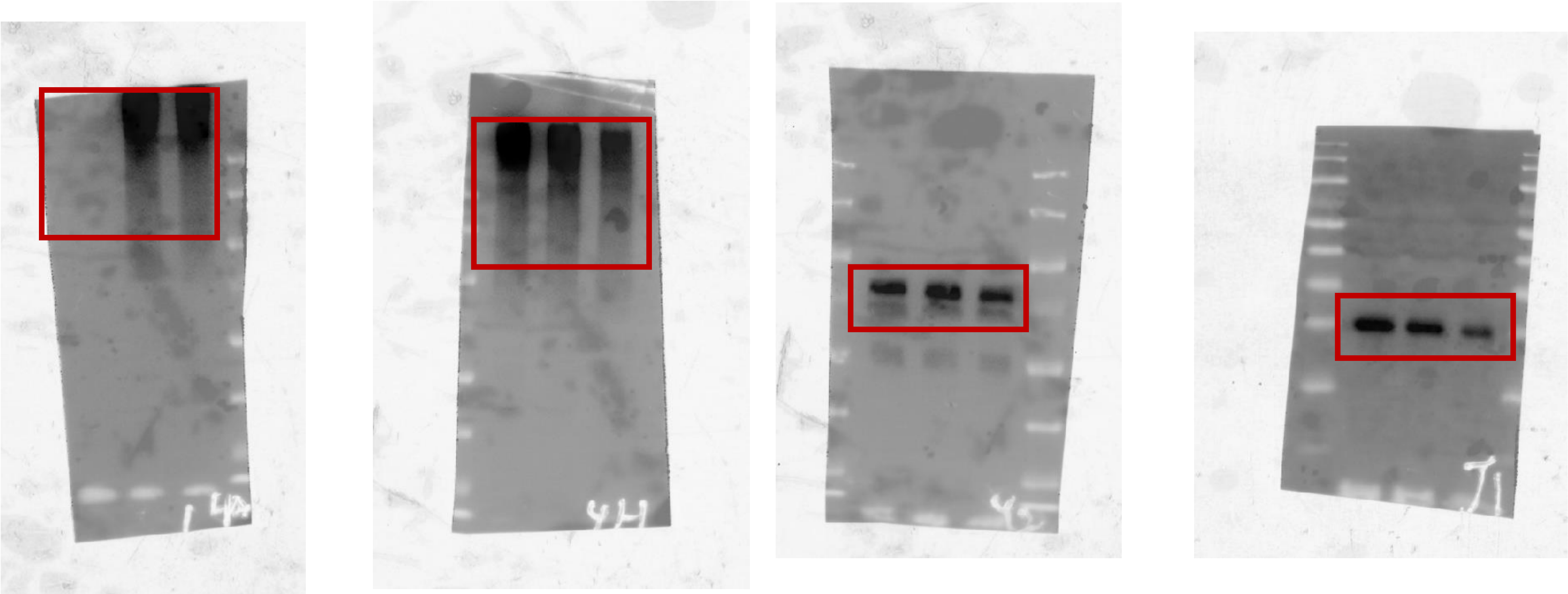

HA (HA-K48R-Ub) | HA (HA-K48R-Ub) | YAP | JOSD1

Figure 6G

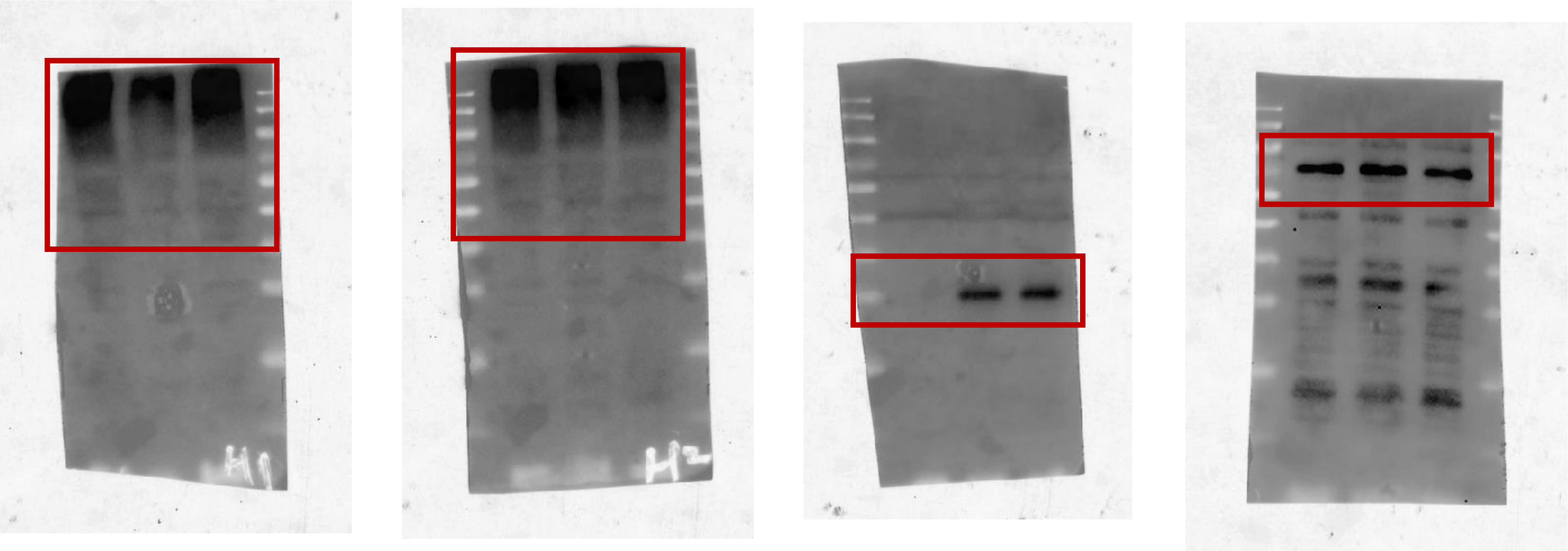

HA
(HA-Ub)

HA
(HA-Ub)

Flag
(Flag-YAP)

Myc
(Myc-JOSD1)

Figure 6H

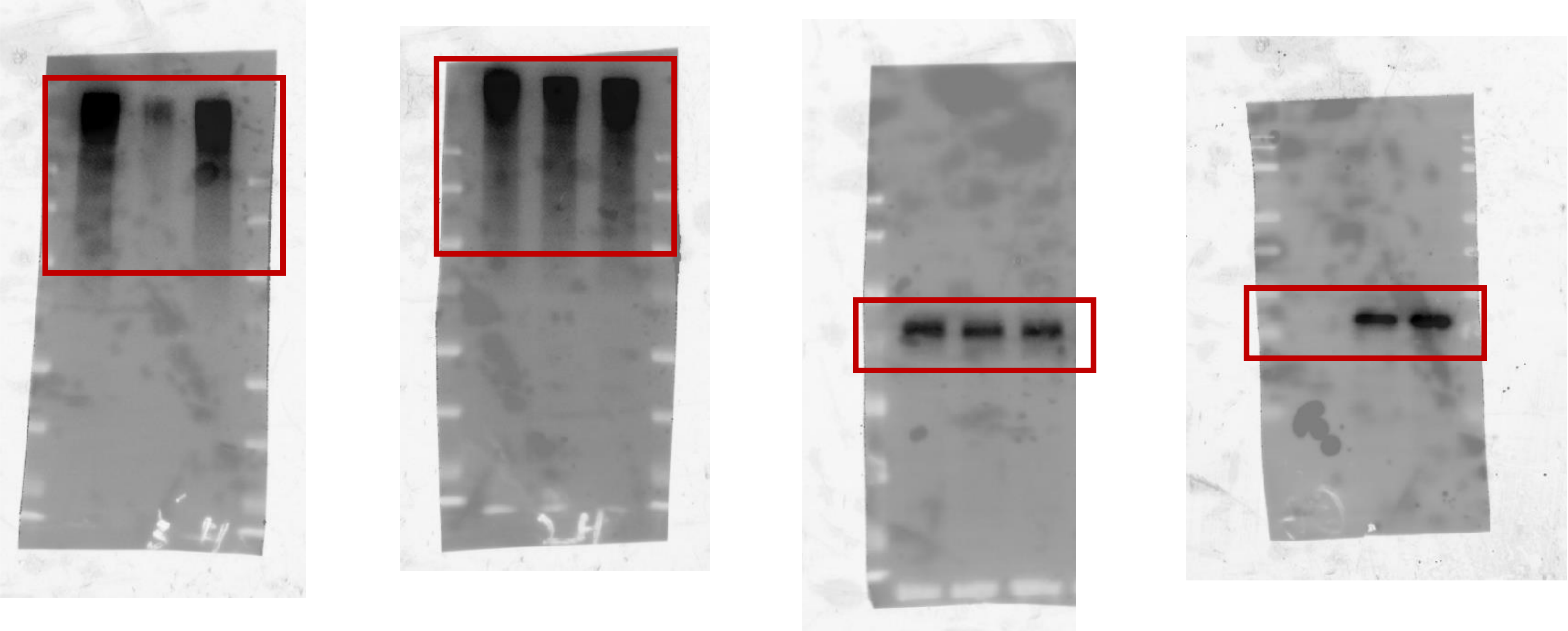

HA (HA-K48-Ub)　　HA (HA-K48-Ub)　　Flag (Flag-YAP)　　Myc (Myc-JOSD1)

Figure 6I

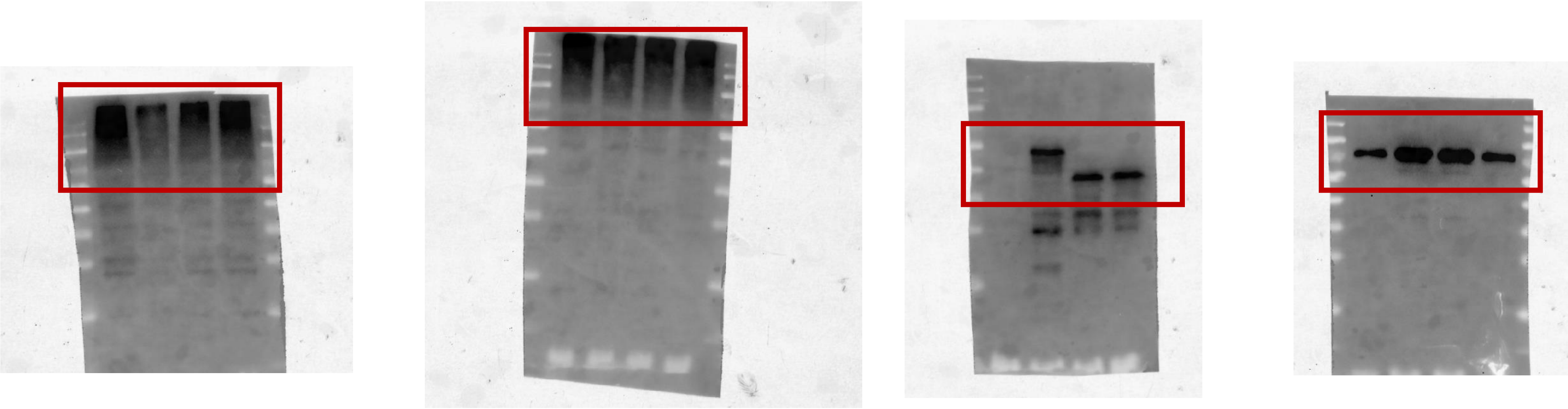

HA (HA-Ub)

HA (HA-Ub)

EGFP (EGFP-JOSD1)

Flag (Flag-YAP)

Figure 6K

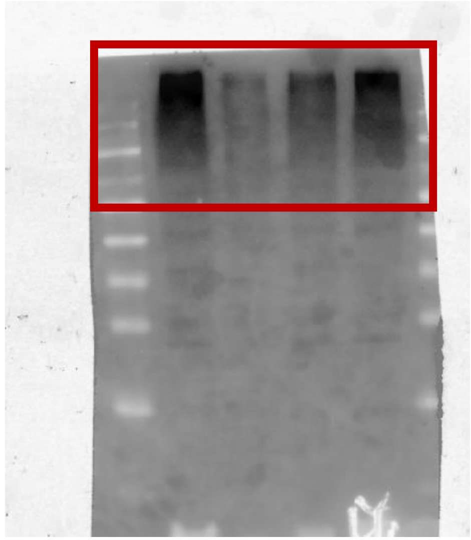

HA
(HA-K48-Ub)

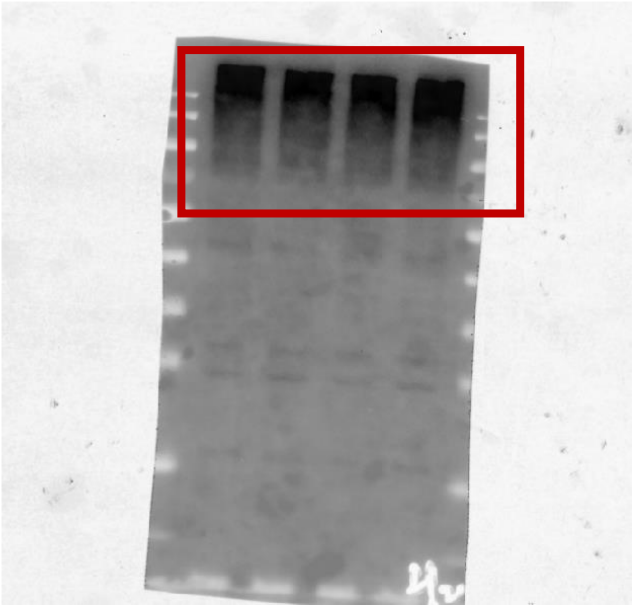

HA
(HA-K48-Ub)

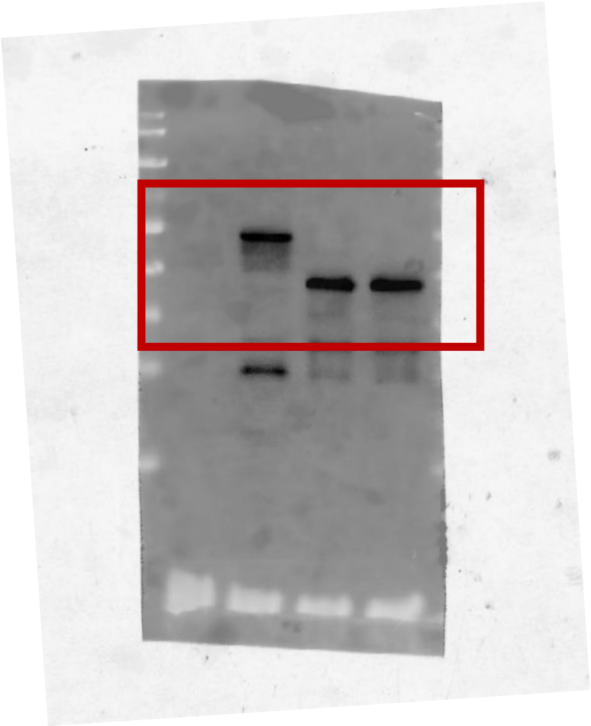

EGFP
(EGFP-JOSD1)

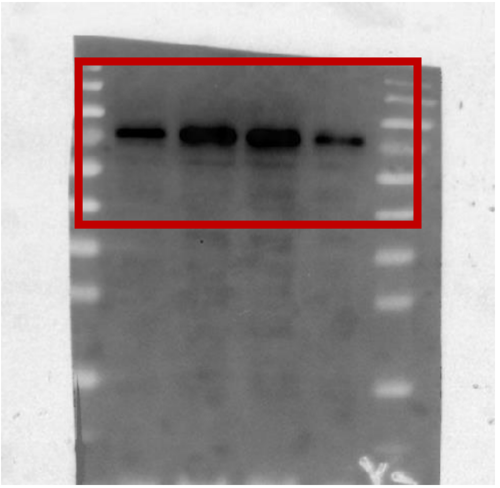

Flag
(Flag-YAP)

Figure 7C

	siControl			siYAP		
IgG	1.006695	0.996831	0.996474	0.999957	1.004199	0.995844
Anti-YAP	6.833497	6.839593	6.827406	2.564648	2.425282	2.493992

Figure 7D

	siControl			siYAP		
IgG	1.009958	0.989887	1.000155	0.995391	1.011522	0.993087
Anti-YAP	9.78112	9.460243	9.149893	3.172778	3.029054	3.100083

Figure 7E

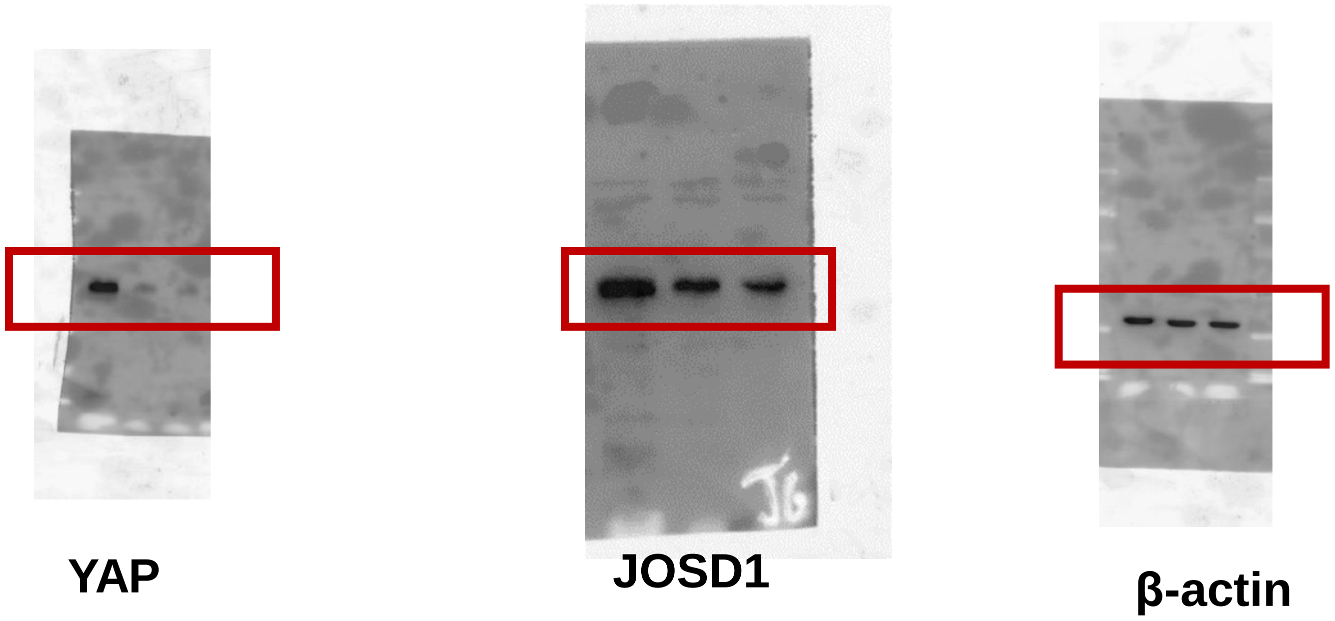

Figure 7F

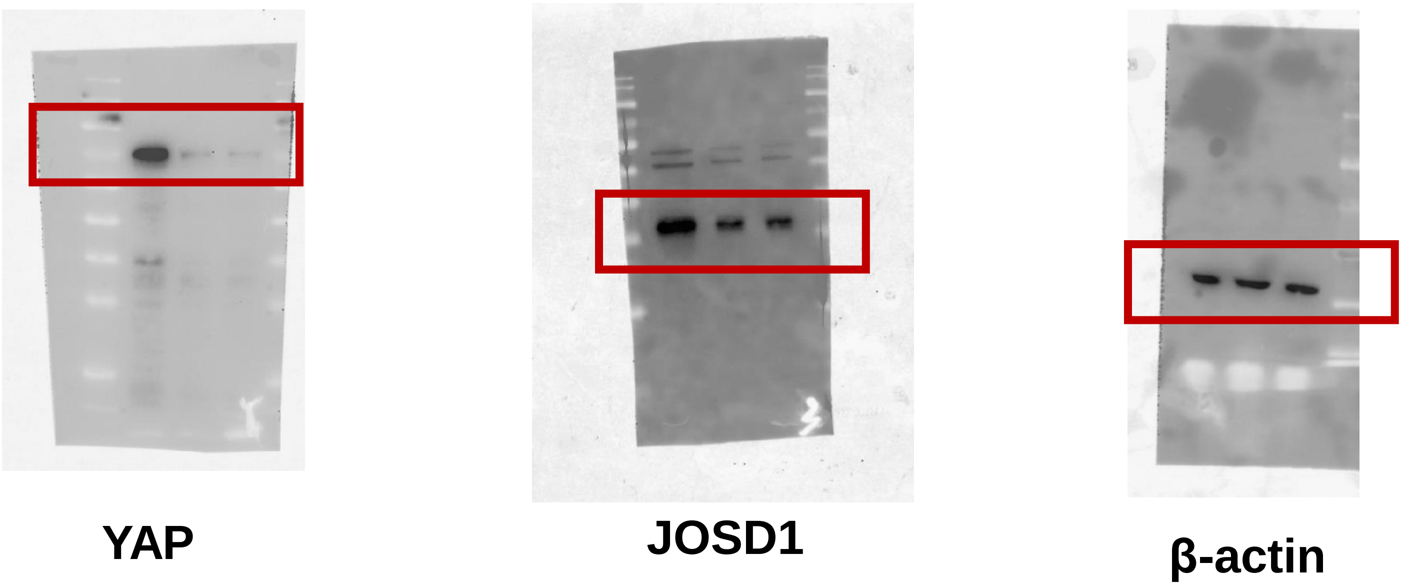

Figure 7G

	siControl			siYAP#1			siYAP#2		
CYR61	0.931855	1.069729	0.998415	0.402846	0.443073	0.487318	0.328354	0.228034	0.273635
JOSD1	0.767859	1.113763	1.118378	0.314537	0.314944	0.283097	0.306688	0.258839	0.286773

Figure 7H

	siControl			siYAP#1			siYAP#2		
CYR61	0.83813	1.089733	1.072136	0.363626	0.386752	0.373415	0.284346	0.328271	0.329177
JOSD1	0.998494	0.980947	1.020559	0.234844	0.232694	0.263514	0.187698	0.182649	0.185991

Figure 7I

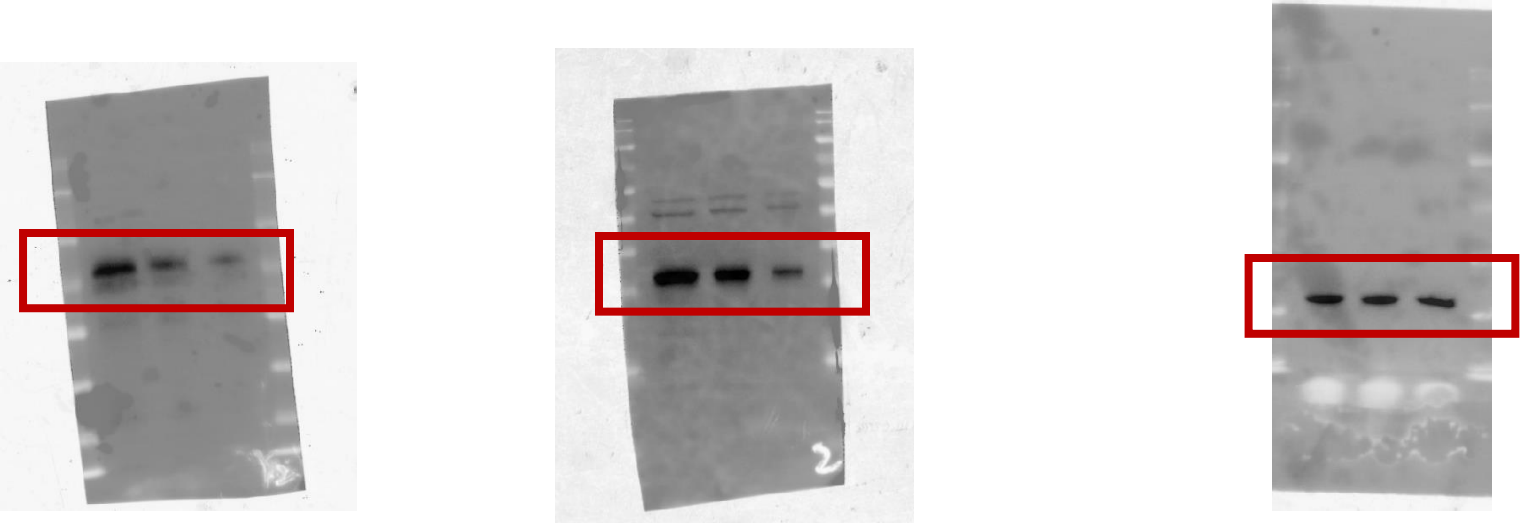

YAP JOSD1 β-actin

Figure 7J

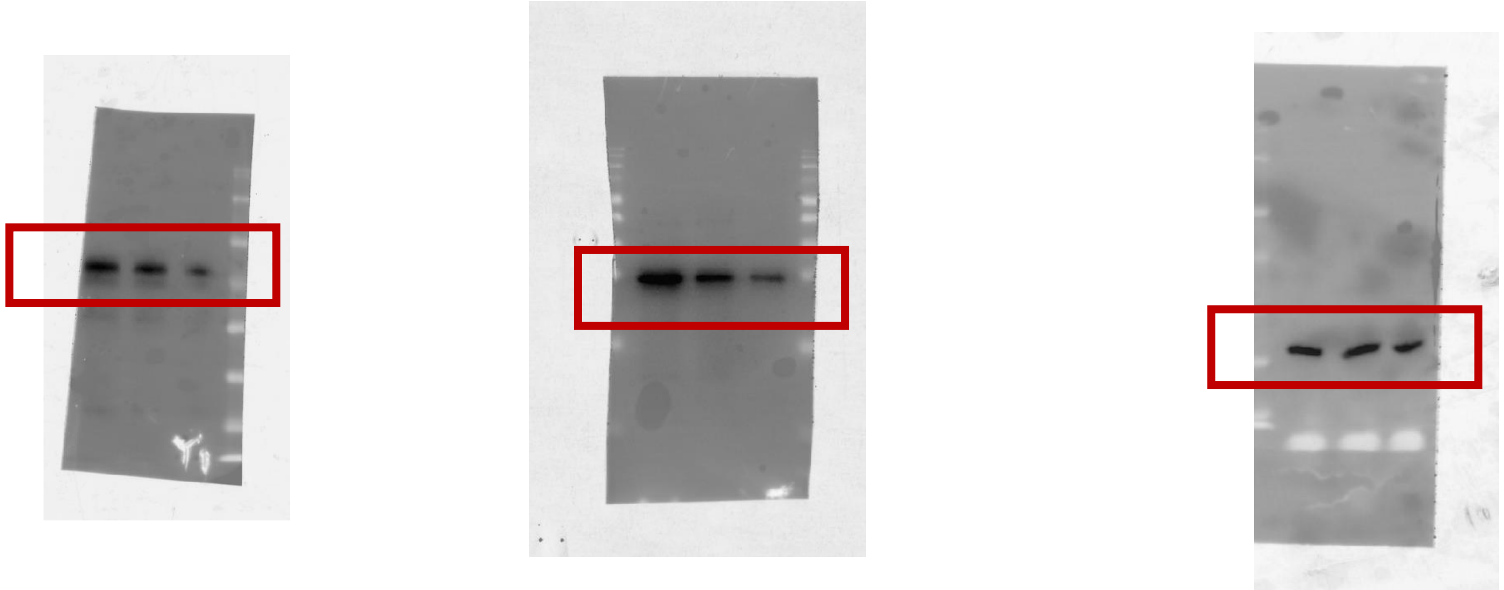

YAP JOSD1 β-actin

Figure 7K

	Vehicle			Verteporfin 2uM			Verteporfin 4uM		
CYR61	0.803029	1.205333	0.991638	0.254311	0.348312	0.241922	0.17831	0.163674	0.169828
JOSD1	0.812056	1.196642	0.991302	0.257125	0.236405	0.320384	0.177412	0.22937	0.186382

Figure 7L

	Vehicle			Verteporfin 2uM			Verteporfin 4uM		
CYR61	0.907031	1.181345	0.911624	0.264468	0.2895	0.274323	0.20309	0.249182	0.246201
JOSD1	0.918064	1.095955	0.985981	0.176897	0.215539	0.227968	0.12827	0.119062	0.112385

Figure 7M

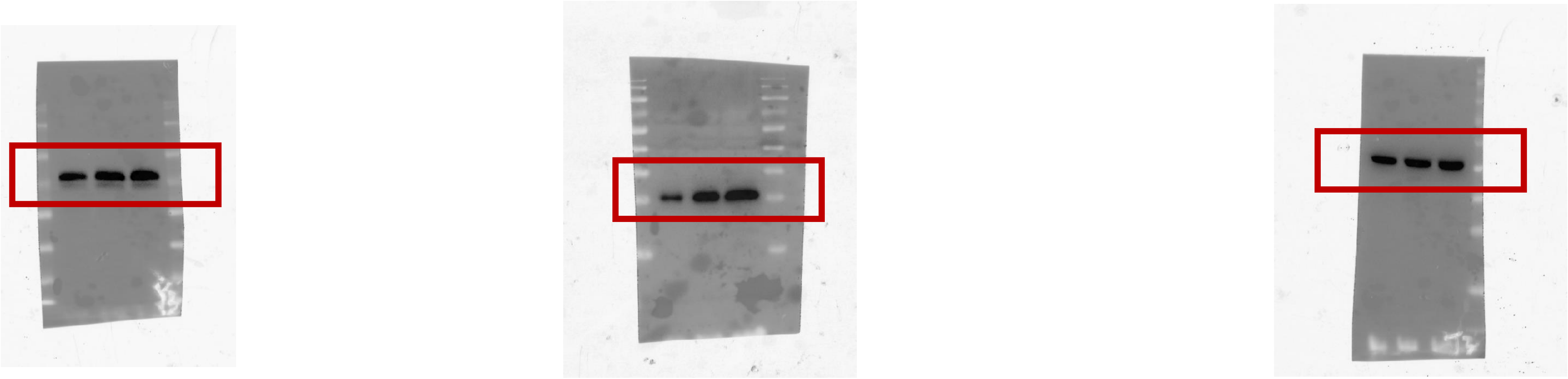

YAP JOSD1 β-actin

Figure 7N

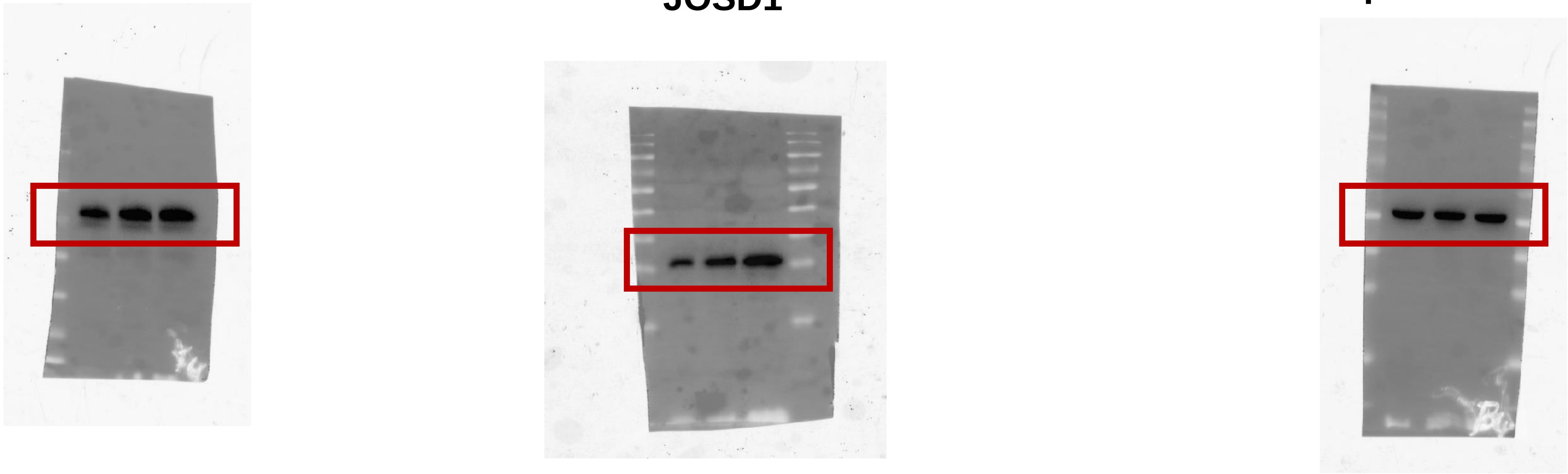

YAP JOSD1 β-actin

Figure 7O

	Vehicle			XMU-MP-1 2uM			XMU-MP-1 4uM		
CYR61	0.877272	0.942066	1.180662	2.402137	1.939268	2.27554	5.608655	6.045515	6.126381
JOSD1	0.952154	0.829608	1.218237	1.771275	1.422685	1.686515	3.186074	2.884276	2.918178

Figure 7P

	Vehicle			XMU-MP-1 2uM			XMU-MP-1 4uM		
CYR61	1.034624	1.002291	0.963084	2.4538	2.216081	1.75114	2.70167	3.387117	2.933048
JOSD1	1.091957	0.96962	0.938423	1.551782	1.692602	1.348577	1.983112	2.453584	2.037836

Figure 7R

Vehicle	Verteporfin
0.900144	0.295337
0.844335	0.260412
1.255521	0.349166

Figure 7T

Vehicle	Verteporfin
0.916047	0.303519
1.218756	0.233434
0.865197	0.169226

Supplementary Figure 2A

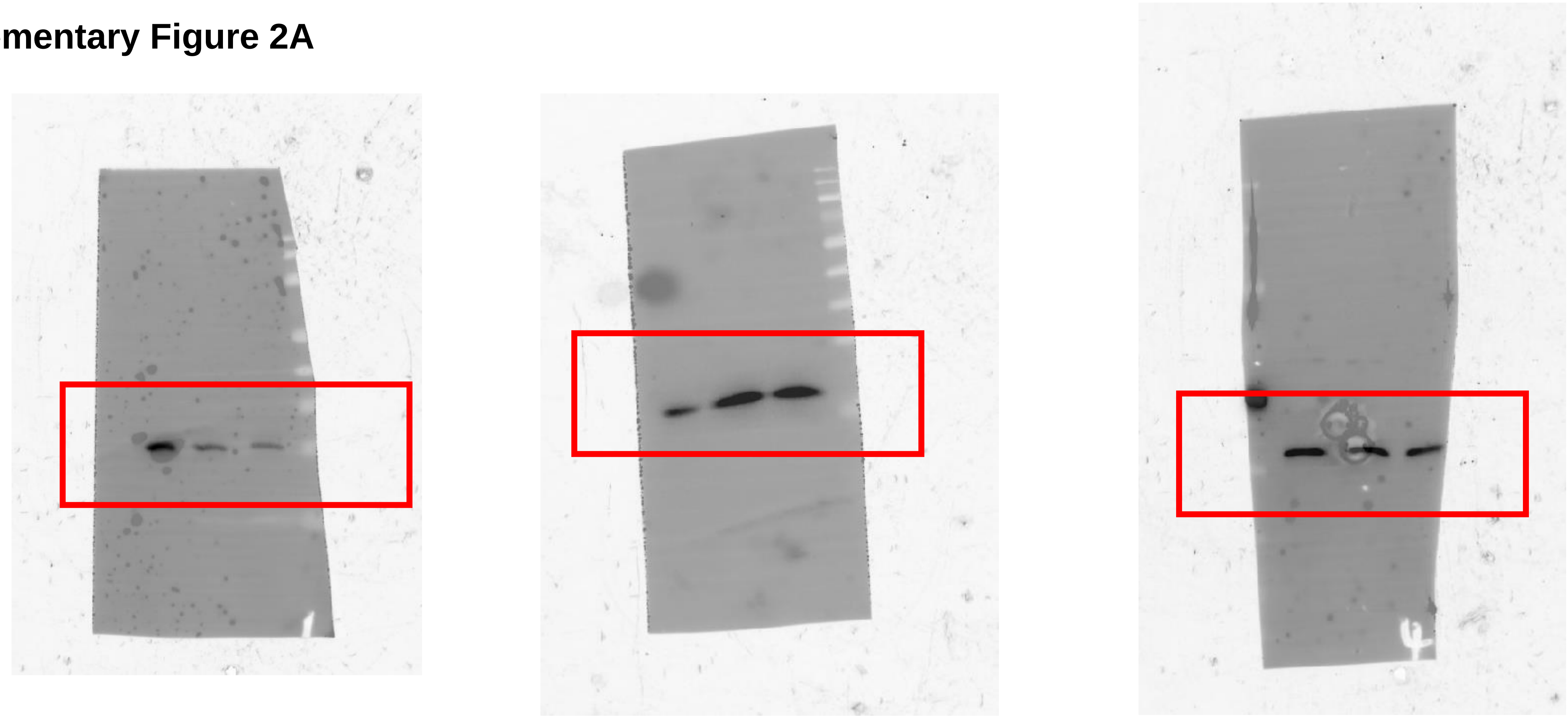

Supplementary Figure 2B

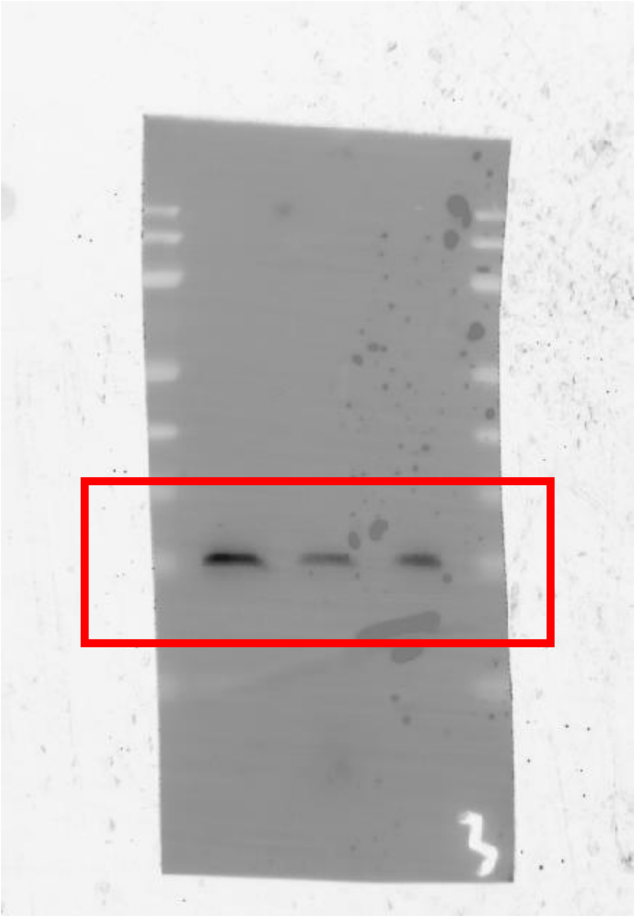

JOSD1

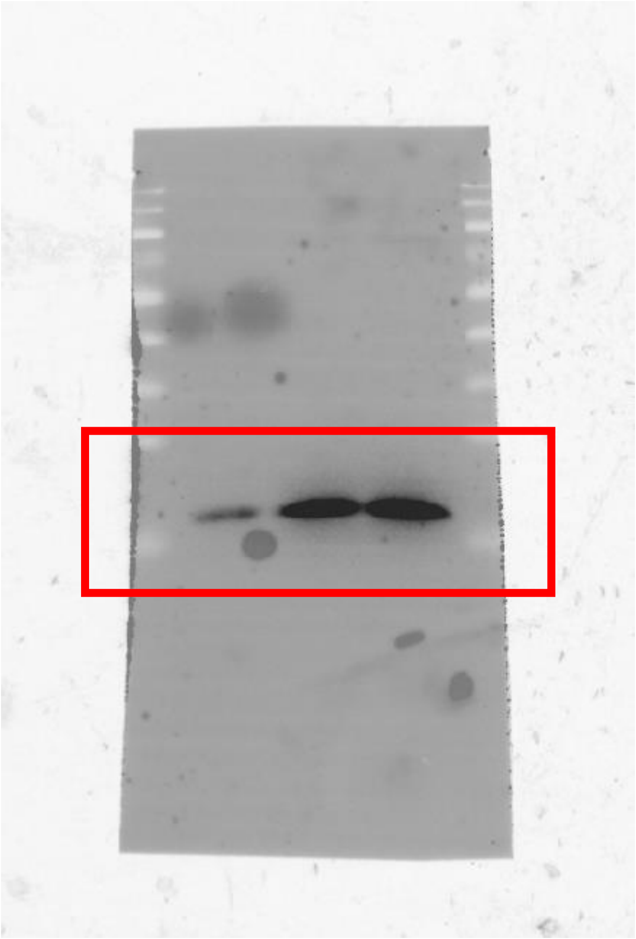

Cleaved Caspase 3

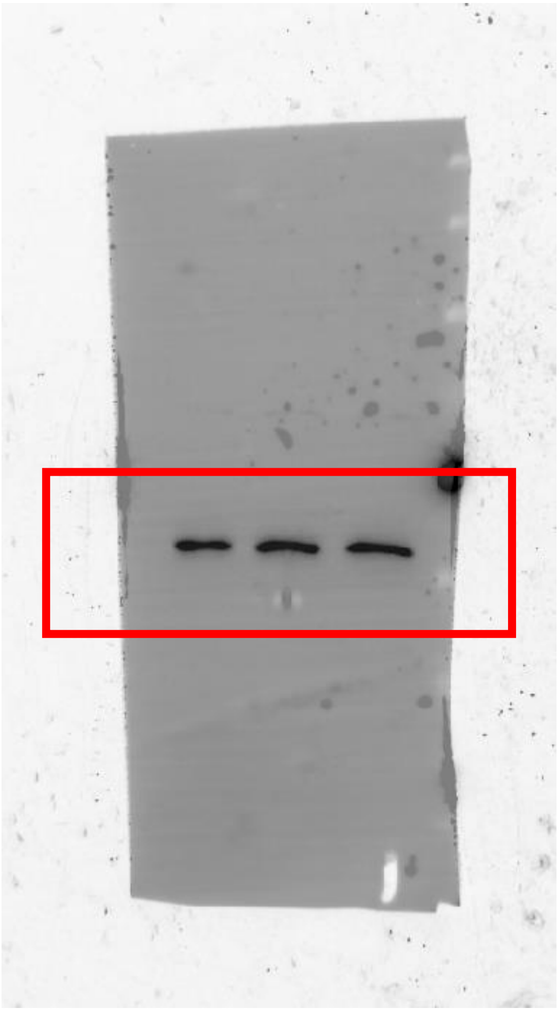

β-actin

Supplementary Figure 2C

	siControl			siJOSD1		
0	1	0.985498	1.014502	1.087524	0.912476	1
5	0.919865	0.915341	0.986365	0.772089	0.788279	0.766765
10	0.832975	0.837499	0.828451	0.645176	0.638114	0.620076
20	0.604966	0.591951	0.666322	0.461327	0.411235	0.466108
40	0.427621	0.395641	0.400971	0.211413	0.226516	0.212282
80	0.113746	0.173615	0.160848	0.002354	0.003984	0.035386

Supplementary Figure 2E

Vector	Flag-JOSD1
3	9
2	14
4	7
2	11
7	11

Supplementary Figure 3A

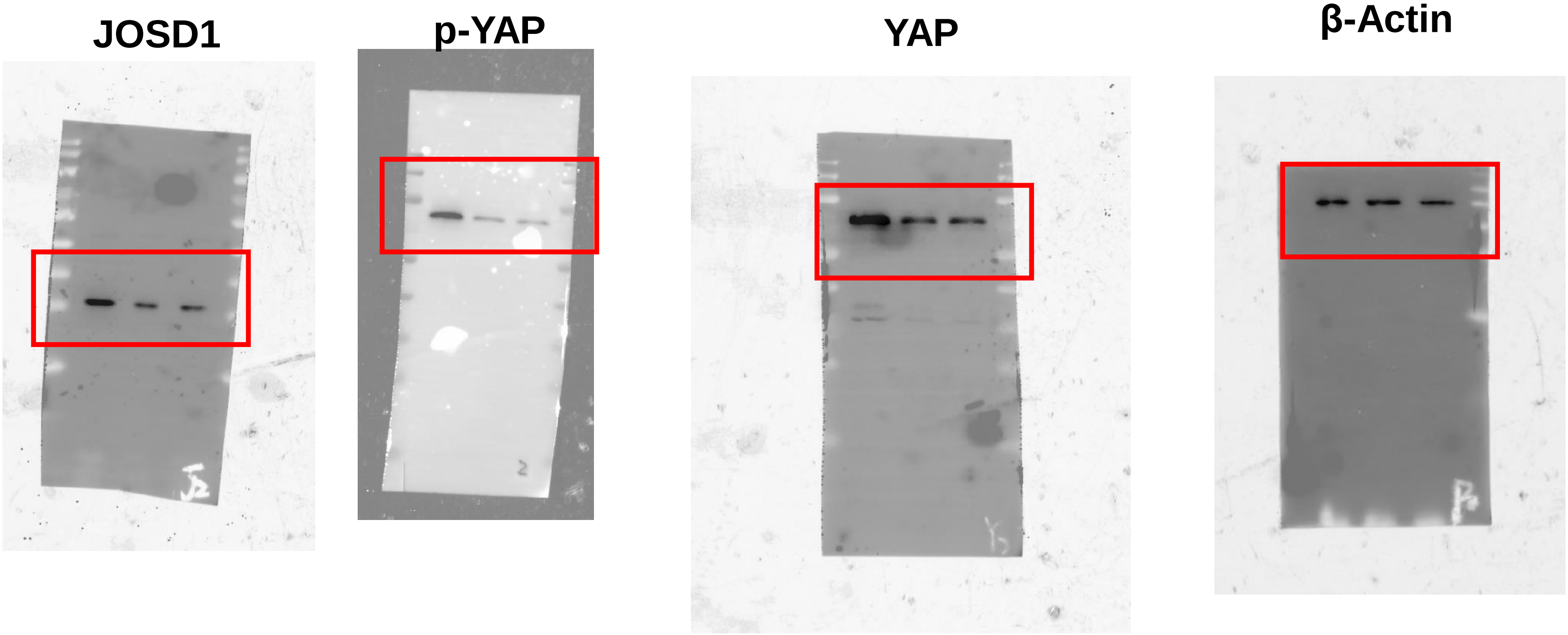

Supplementary Figure 3B

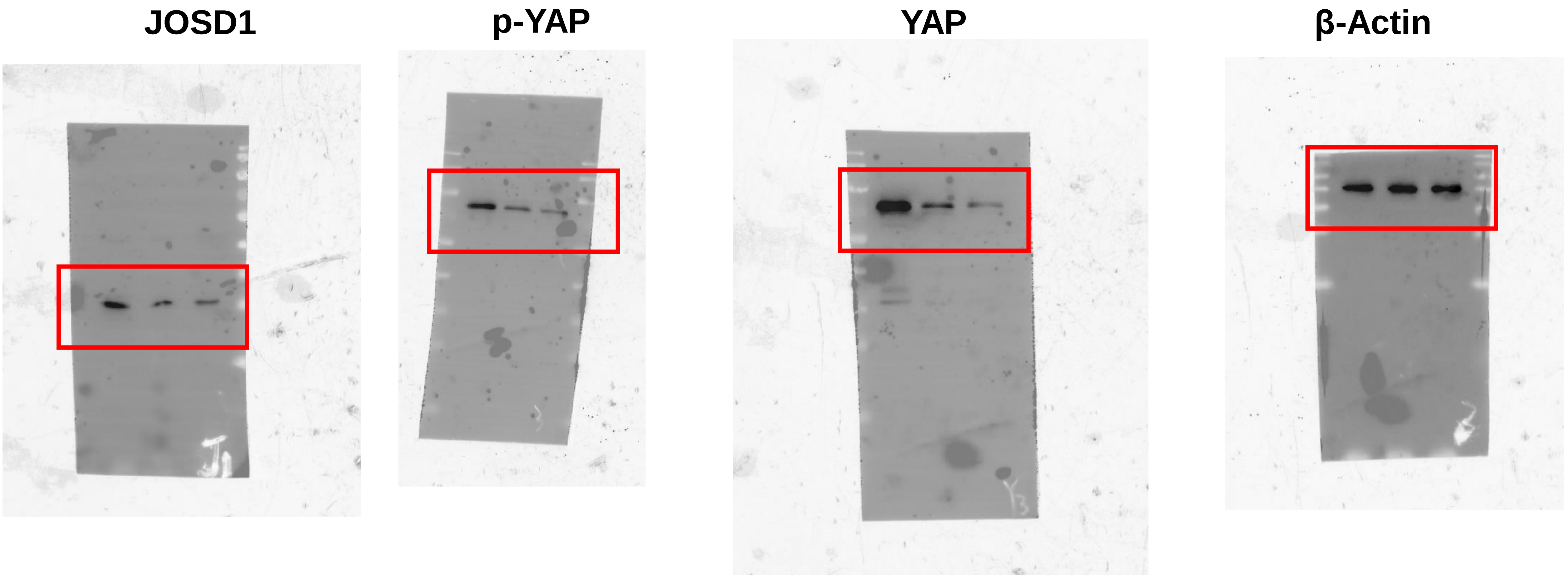

Supplementary Figure 3C

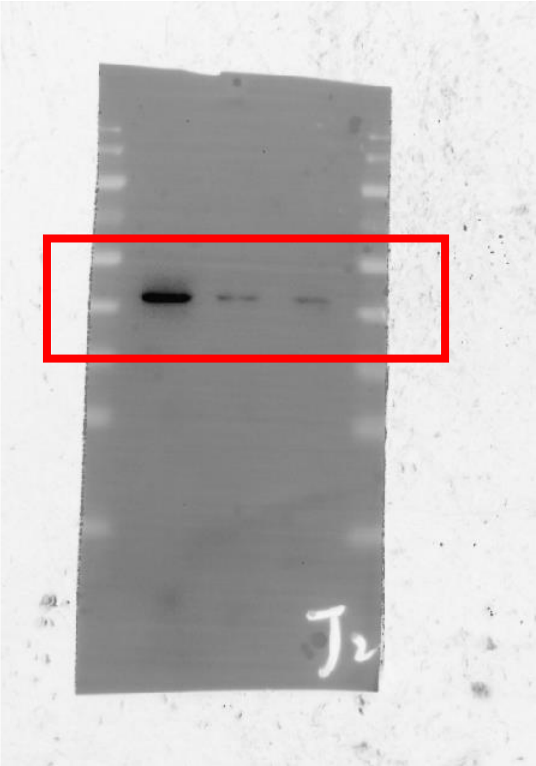

JOSD1

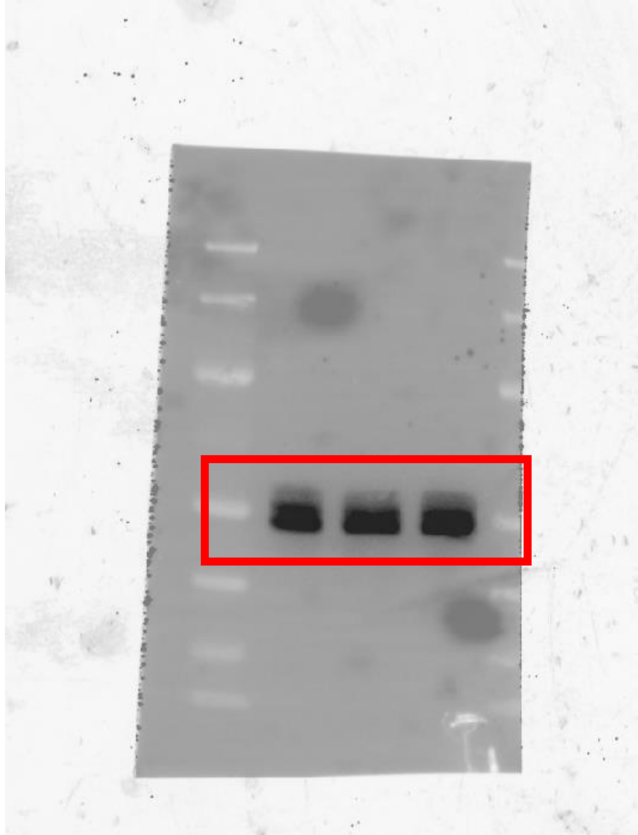

TAZ

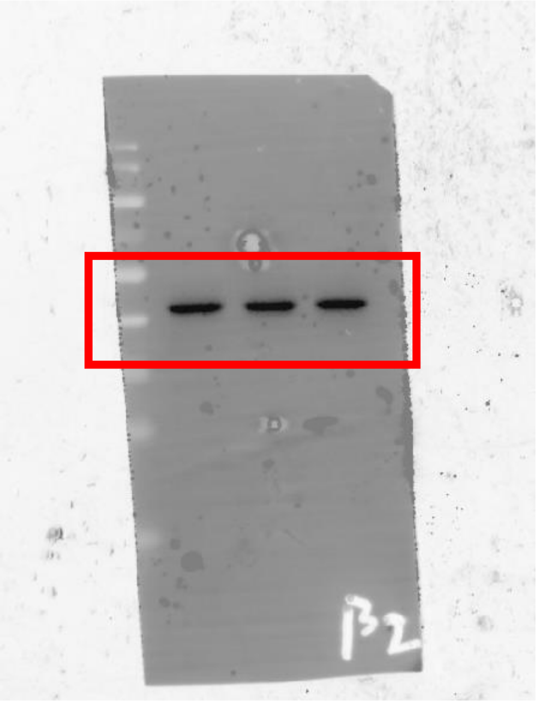

β-actin

Supplementary Figure 3D

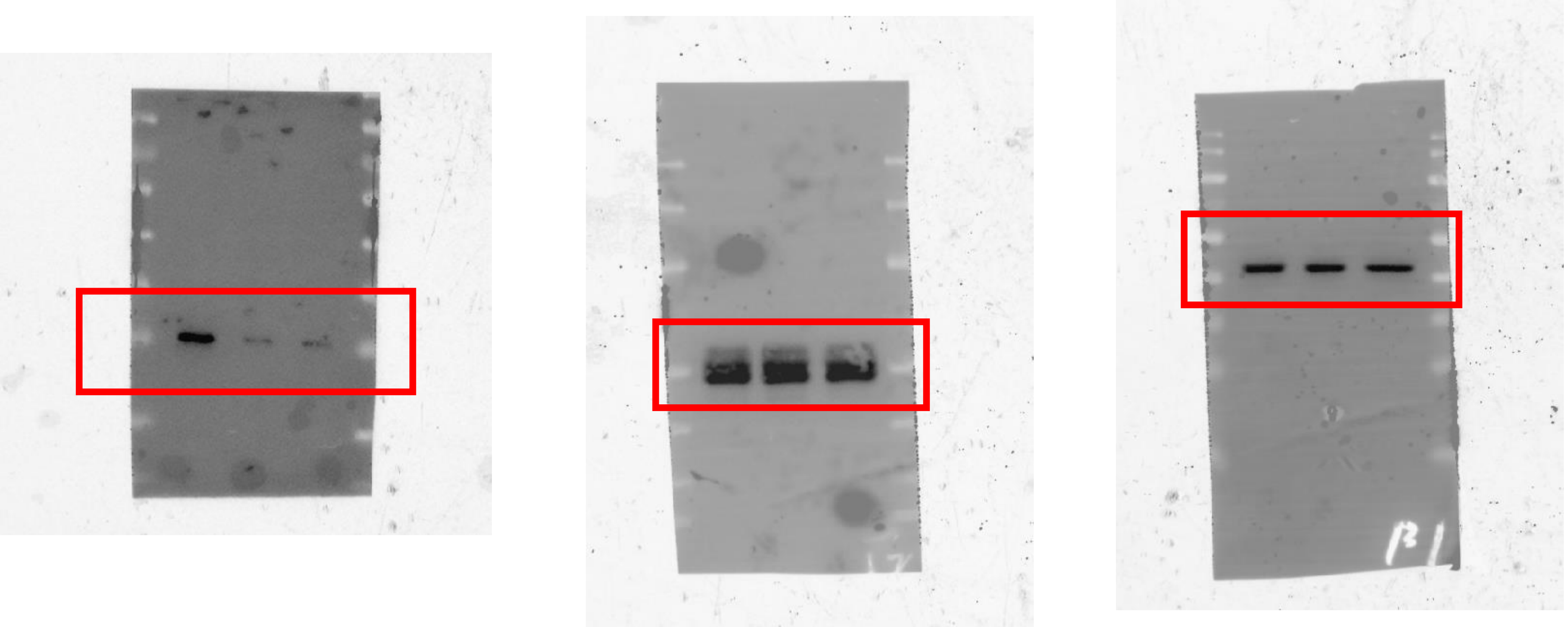

JOSD1 TAZ β-Actin

Supplementary Figure 3E

JOSD1 **p-YAP** **YAP** **β-Actin**

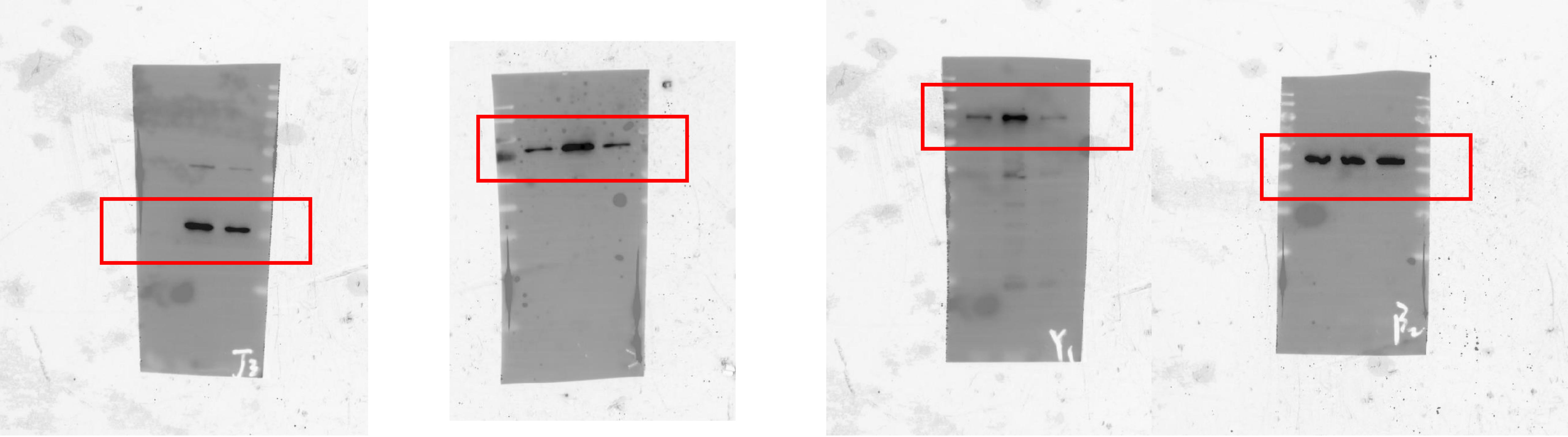

Supplementary figure 4A

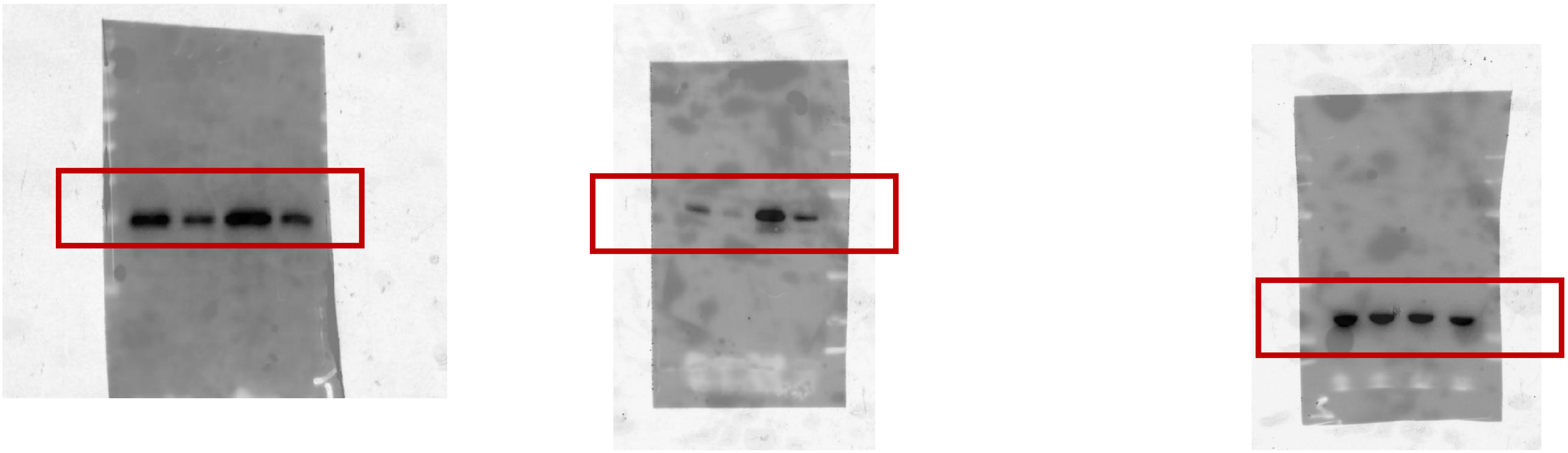

JOSD1 **YAP** **β-actin**

Supplementary figure 4B

	siControl			siJOSD1			YAP			siJOSD1+YAP		
CYR61	0.909477	1.217965	0.872559	0.245464	0.181803	0.233392	5.048291	5.086269	4.845234	0.926228	0.894055	1.040121
CTGF	0.952035	0.982723	1.065243	0.421281	0.207132	0.358269	2.036788	2.260217	2.009381	1.094331	1.409623	0.747931

Supplementary figure 4C

siControl	siJOSD1	YAP	siJOSD1+YAP
0.999983	0.208746	1.961864	1.279897
0.992827	0.145899	2.397747	1.187256
1.00719	0.135397	2.339624	1.339288

Supplementary figure 4D

Time(h)	siControl			siJOSD1			YAP			siJOSD1+YAP		
0	1.011407	0.980989	1.007604	0.961228	0.961228	1.013732	1.068376	0.920746	1.056721	0.974771	1.001529	1.03211
24	2.395437	2.079848	2.091255	1.550889	1.437803	1.587237	3.411033	3.030303	3.022533	1.995413	2.090979	2.133028
48	5.361217	5.43346	5.498099	2.940226	2.742326	2.778675	7.327117	7.331002	7.226107	4.980887	5.08792	5.057339
72	8.391635	8.095057	8.30038	4.955574	5.084814	5.214055	12.73893	12.64569	12.59907	7.86315	7.584098	7.652905

Supplementary figure 4F

siControl	siJOSD1	YAP	siJOSD1+YAP
0.899579	0.30872447	3.18737682	1.017847
0.954679	0.3367868	3.0876348	0.967362
1.145742	0.278237487	2.5982749	0.870108

Supplementary figure 4H

Time(h)	siControl			siJOSD1			YAP			siJOSD1+YAP		
0	0	0	0	0	0	0	0	0	0	0	0	0
24	0.590909	0.62987	0.649351	0.227737	0.264964	0.244453	0.753425	0.732877	0.815068	0.571429	0.526316	0.616541
48	0.707792	0.766234	0.785714	0.405839	0.440146	0.527737	0.958904	0.979452	0.89726	0.684211	0.774436	0.706767

Supplementary figure 4J

siControl	siJOSD1	YAP	siJOSD1+YAP
0.92545	0.262211	2.745501	1.056555
1.056555	0.33162	2.899743	1.133676
1.017995	0.215938	2.984576	1.1491

Supplementary figure 4L

siControl	siJOSD1	YAP	siJOSD1+YAP
16.48	24.37	8.53	17.01
15.47	21.53	7.46	15.19
14.6	26.57	7.64	15.36

Supplementary figure 5A

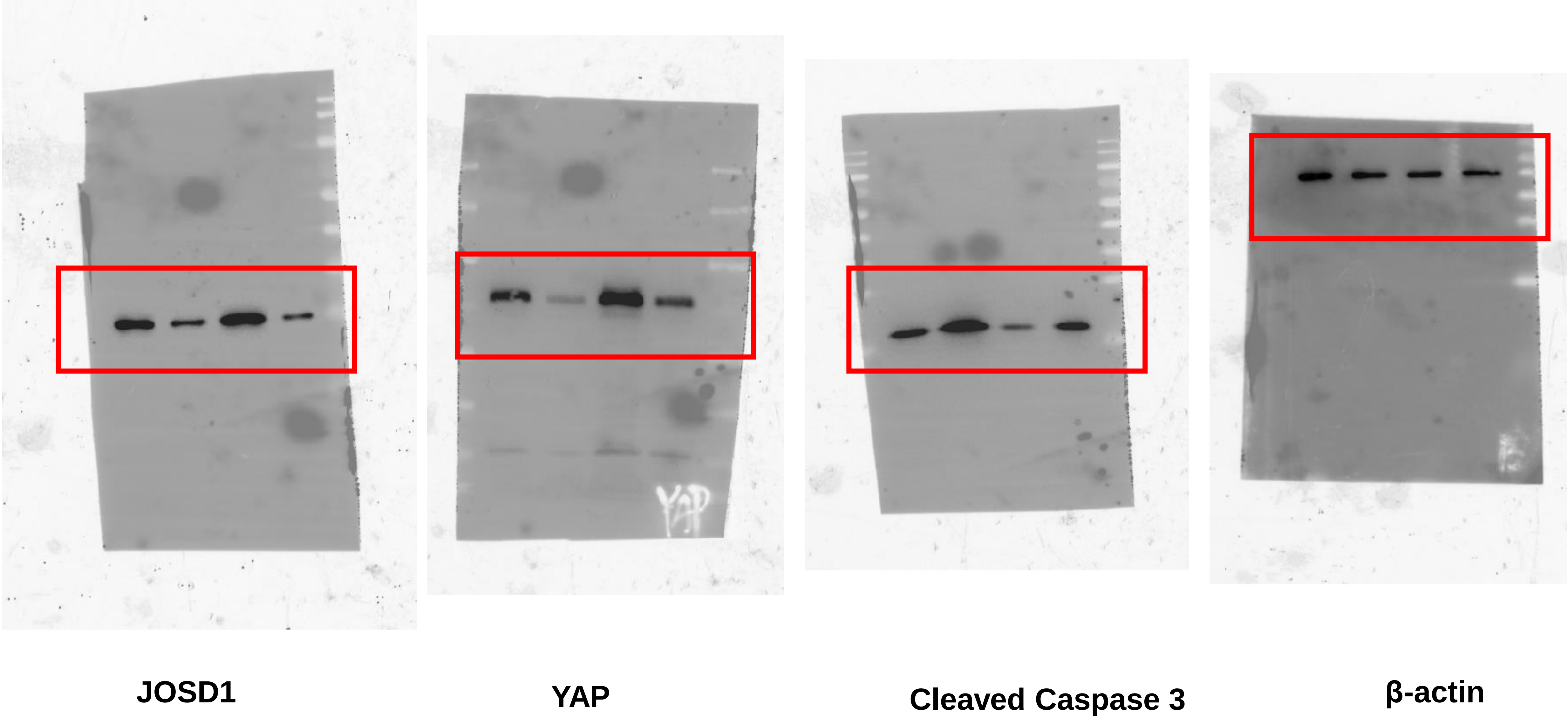

Supplementary figure 5C

siControl	siJOSD1	YAP	siJOSD1+YAP
1.030973	0.429204	1.420354	1.026549
1.066372	0.353982	1.495575	0.902655
0.902655	0.469027	1.300885	0.986726

Supplementary Figure 6A

YAP

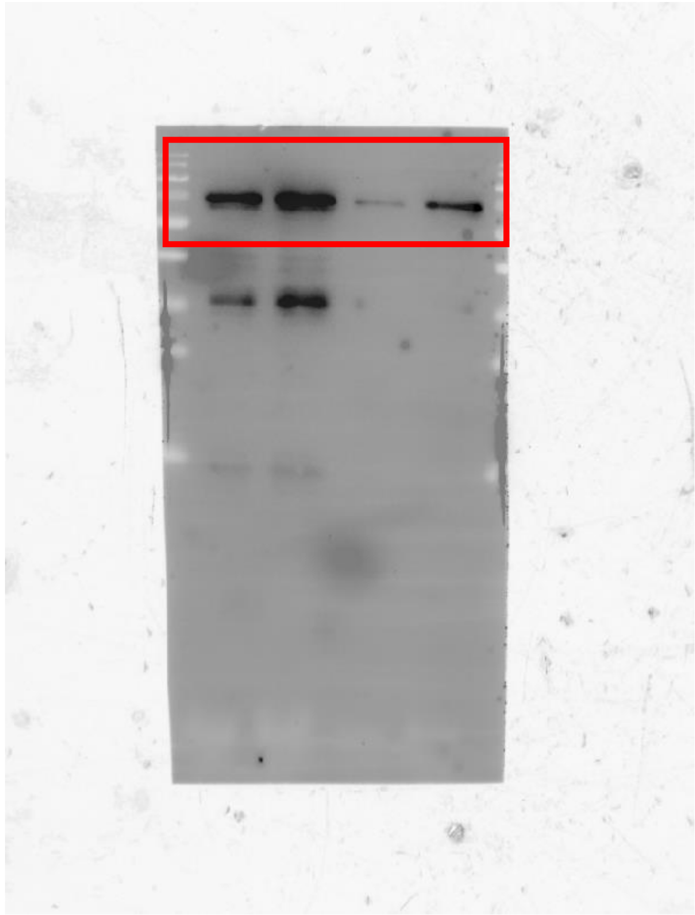

Tubulin

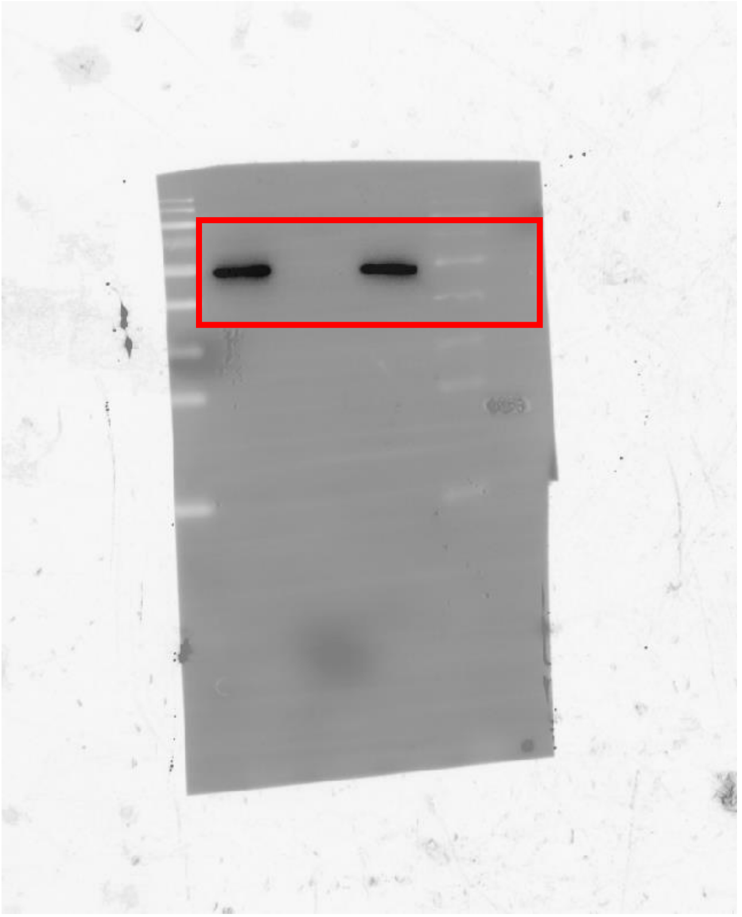

Histone 3

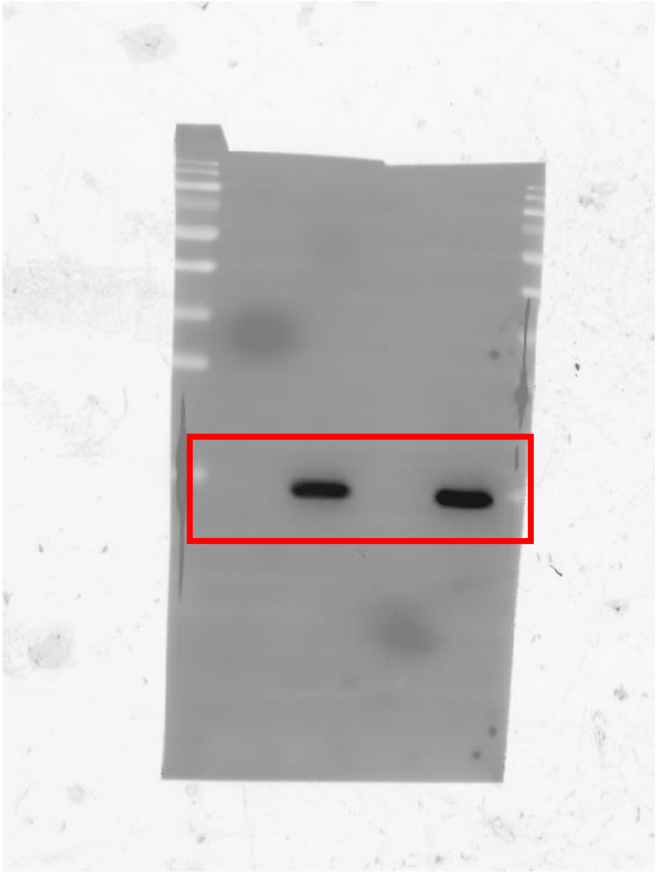

Supplementary Figure 6B

YAP

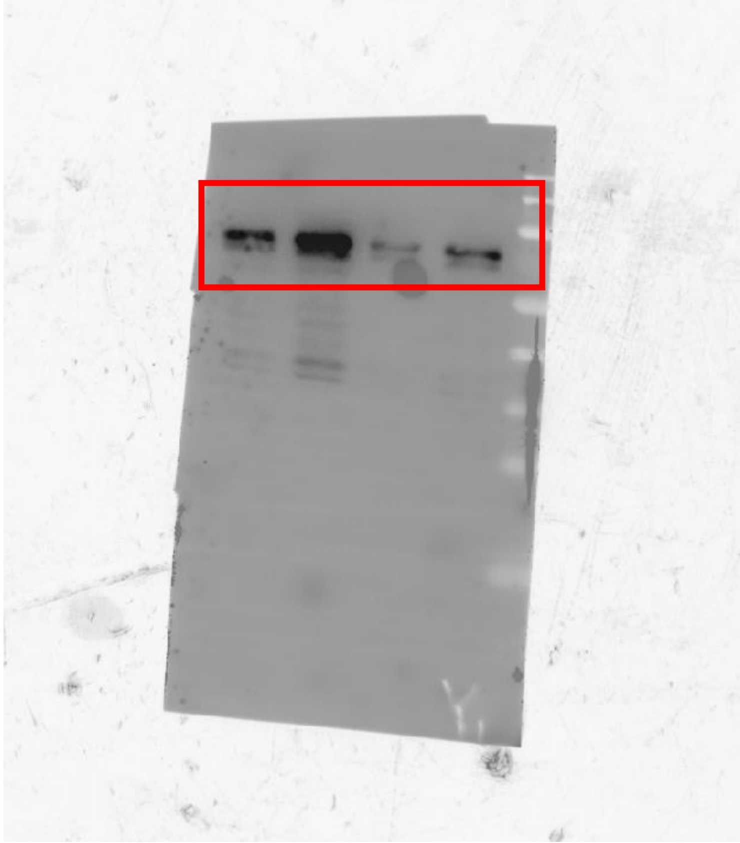

Tubulin

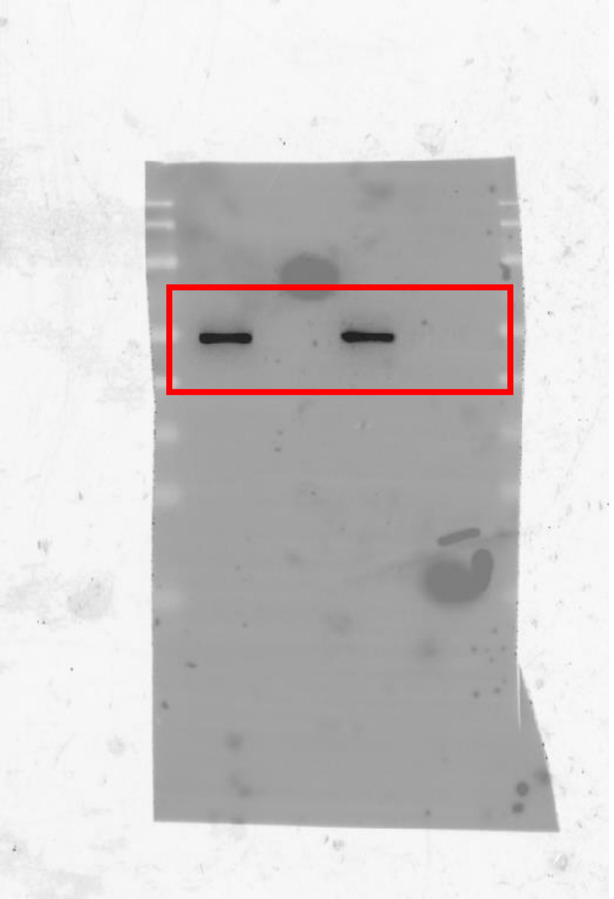

Histone 3

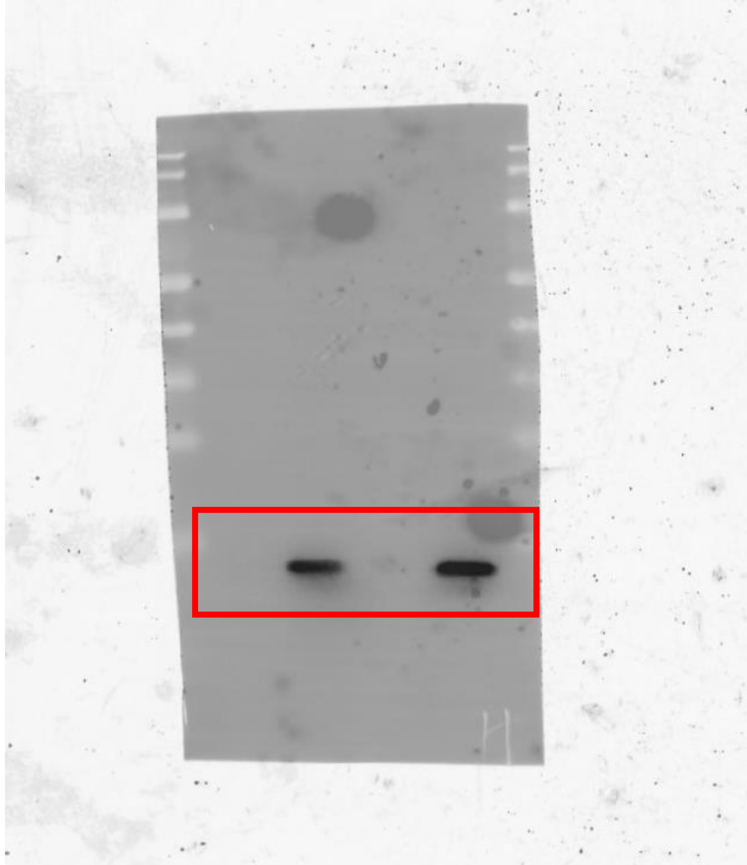

Supplementary Figure 6D

	Cytoplasm			Nucleus		
siControl	0.325662	0.402576	0.390722	0.674338	0.597424	0.609278
siJOSD1	0.19341	0.234885	0.225056	0.409035	0.355911	0.364625

Supplementary Figure 6F

	Cytoplasm			Nucleus		
siControl	0.238654	0.342775	0.381703	0.761346	0.657225	0.618297
siJOSD1	0.144761	0.204206	0.108741	0.452162	0.383461	0.471339

Supplementary Figure 6G

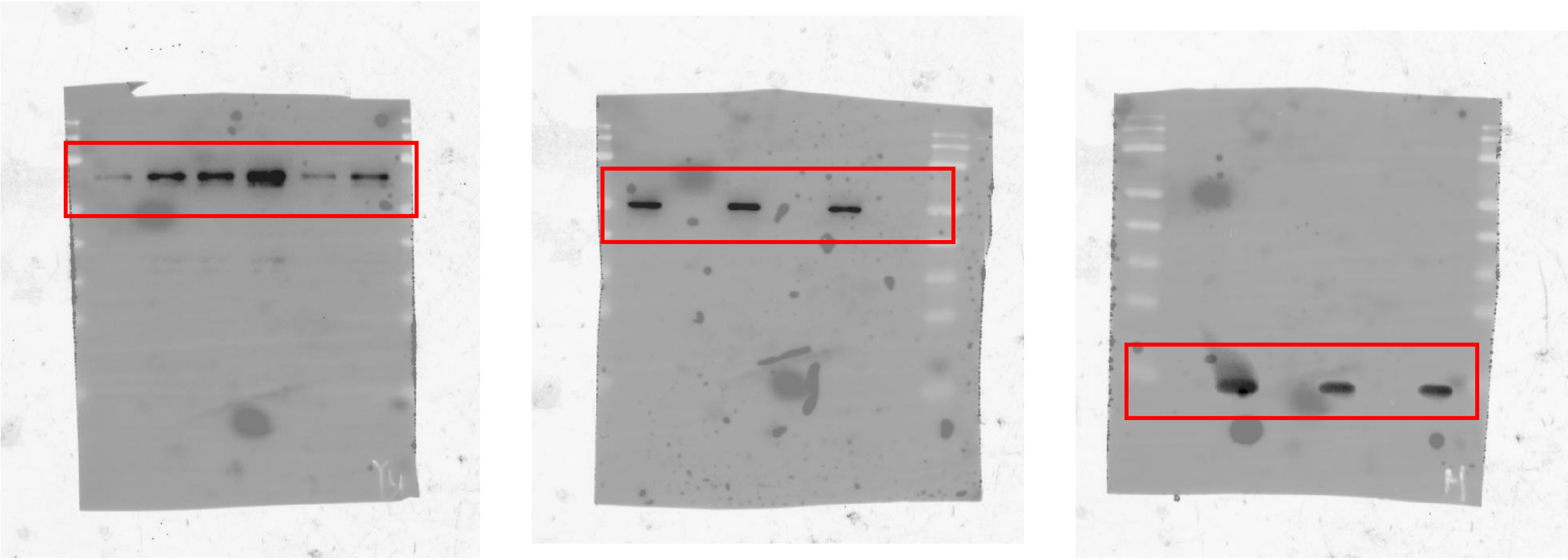

Supplementary Figure 6I

	Cytoplasm			Nucleus		
Flag	0.240987	0.322065	0.336948	0.759013	0.677935	0.663052
Flag-JOSD1	0.960797	1.078805	1.300437	1.680994	1.412759	1.40832
FLAG-JOSD1^{C36A}	0.270205	0.356114	0.312924	0.932599	0.770636	0.774348
